# Pyrolysis-Oil Methoxyphenol Extraction: From Biobased Solvent Screening to Thermodynamic Modeling

**DOI:** 10.1021/acs.energyfuels.6c02836

**Published:** 2026-08-14

**Authors:** Sebastián Ormazábal-Latorre, Esteban Cea-Klapp, Maximilian Fleckenstein, Nicolás F. Gajardo-Parra, Ady Giordano, Andrea Sánchez-Monedero, Emilio J. González, María González-Miquel, Roberto I. Canales

**Affiliations:** † Departamento de Ingeniería Química y Bioprocesos, Pontificia Universidad Católica de Chile, Santiago 7820436, Chile; ‡ Facultad de Química y de Farmacia, Pontificia Universidad Católica de Chile, Santiago 7820436, Chile; § Departamento de Ingeniería Química Industrial y del Medio Ambiente, ETSI Industriales, Universidad Politécnica de Madrid, C/José Gutiérrez Abascal 2, Madrid 28006, Spain

## Abstract

The valorization of pyrolysis bio-oil into fuels and renewable chemicals relies on separation steps that recover its most valuable constituents, and liquid–liquid extraction with biobased solvents offers a sustainable route to isolate lignin-derived methoxyphenols. This study combines in silico solvent screening, experimental liquid–liquid equilibrium measurements, and thermodynamic modeling to recover guaiacol and 2,6-dimethoxyphenol from a seven-component synthetic bio-oil. Quantum-chemical predictions with COSMO-RS narrowed more than 1700 candidates to 156 solvents immiscible with water at 313.15 K, which a five-step sustainability-and-availability cascade and a multicriteria decision-analysis ranking reduced to a shortlist of 40. Dimethyl carbonate, cyclopentyl methyl ether, and 2-methyltetrahydrofuran were retained for experimental validation, with 2-butanol as a thermodynamic negative reference. Dimethyl carbonate matched or exceeded the conventional extractants *n*-hexane and toluene in predicted capacity while keeping a favorable safety, health, and environmental profile. Ternary phase diagrams confirmed wide miscibility gaps in both synthetic and real bio-oil, the latter showing pyrolytic-lignin precipitation that constrains the operating window. Across 20 ternary systems, the PC-SAFT equation of state was the most accurate (RMSD of 0.0310), ahead of the purely predictive COSMO-RS (0.0478) and the Modified UNIFAC 2.0 model (0.0749). In multicomponent extractions, guaiacol reached a distribution coefficient as high as 50.73 while d-fructose stayed in the water-rich phase, and the fitted PC-SAFT parameters transferred to the seven-component mixture (RMSD of 0.0267), supporting a combined screening-and-modeling strategy for sustainable bio-oil fractionation.

## Graphical abstract

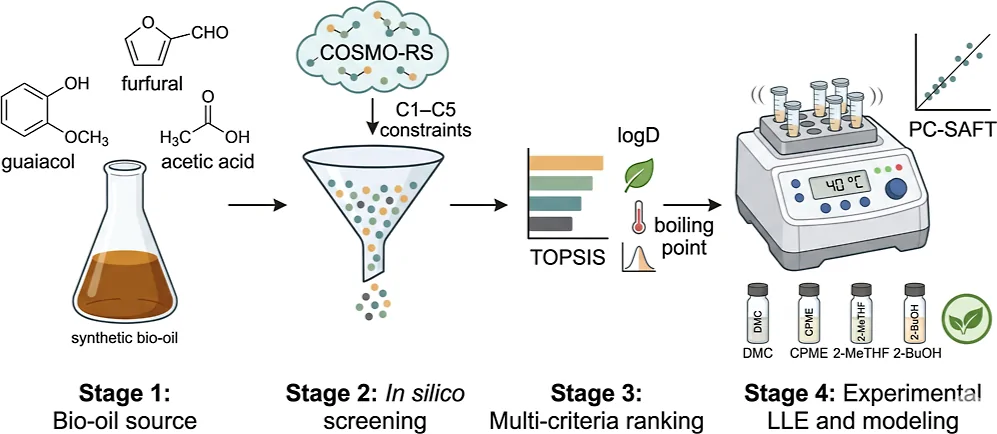

## 1 Introduction

Bio-oil produced through fast pyrolysis of lignocellulosic biomass is a viscous liquid composed of water and numerous oxygenated organic compounds.^1,2^ As a renewable energy carrier, it has attracted significant attention for its potential to displace fossil fuels in power generation, heating, and chemical industries.^3^ Compared with raw biomass, bio-oil offers higher energy density, better transportability, and greater versatility for chemical applications.^4^ Its complex composition, including acids, ketones, aldehydes, furans, and phenolic compounds, results from the thermal breakdown of biomass components.^1^ These properties make it suitable not only as a partial fuel substitute but also as a platform for the production of renewable chemicals.

Despite its promising features, the direct use of crude bio-oil as a fuel is limited by several unfavorable physicochemical properties. Its high water content (up to 30 wt %), low pH, elevated oxygen content, and viscosity are incompatible with fuel standards and contribute to poor ignition, flame instability, and corrosiveness.^1,3^ Furthermore, bio-oil is chemically unstable due to the presence of reactive oxygenates, such as aldehydes, ketones, and phenols, which tend to polymerize during storage or heating, leading to increased viscosity, phase separation, and coke formation.^2^ To improve its fuel properties, upgrading methods such as catalytic hydrodeoxygenation (HDO) have been employed to reduce the oxygen content and increase energy density.^5^ One of the main problems is that catalyst deactivation remains a persistent barrier, driven by coke deposition, sintering, and poisoning. This is particularly problematic in the presence of lignin-derived phenolics such as guaiacol and 2,6-dimethoxyphenol (2,6-DMP) because they are susceptible to repolymerization and the formation of carbonaceous residues.^6–8^ However, the same methoxyphenolic compounds are valuable precursors for renewable chemicals used in resins, pharmaceuticals, and flavors.^9–11^ As a result, there is growing interest in shifting the focus from fuel use to the targeted recovery and valorization of phenolic compounds. Fractionation techniques such as distillation and membrane separation offer promising strategies to isolate and stabilize these compounds before further processing.^12,13^

The most valuable of these constituents are lignin-derived methoxyphenols such as guaiacol, 2,6-DMP, eugenol, and vanillin.^14,15^ However, isolating these methoxyphenols from the complex bio-oil matrix remains technically challenging. Among available separation methods, liquid–liquid extraction has proven especially attractive for its scalability, simplicity, and adaptability to both synthetic and natural bio-oil systems.^12^ This technique transfers target compounds into an immiscible solvent, but its effectiveness depends critically on solvent selection. Traditional solvents such as dichloromethane, chloroform, and *n*-hexane offer good extraction performance but raise safety, health, and environmental concerns due to their toxicity and fossil origin.^16^ In response, sustainability-focused frameworks such as the 12 Principles of Green Chemistry^17^ and the 10R strategies for circular engineering^18^ have driven interest toward biobased solvents derived from renewable sources such as agricultural or forest residues.^16,19^ While these biobased alternatives show promising characteristics, their extraction efficiency is highly sensitive to the solvent’s molecular structure, making exhaustive experimental testing unfeasible. Consequently, computational solvent screening methods such as the Conductor-like Screening Model for Real Solvents (COSMO-RS) have emerged as powerful predictive tools for evaluating solute–solvent affinities and prioritizing environmentally friendly candidates for experimental validation in methoxyphenol recovery.^20–22^

Reliable thermodynamic models are needed to design and simulate bio-oil fractionation processes, but their use is limited by the variable composition of bio-oil and by the scarcity of experimental phase-behavior data for its individual compounds.^1,23,24^ Synthetic bio-oil (SynBO) mixtures, prepared from a few representative compounds, reduce this limitation by allowing controlled measurements and more manageable modeling.^25^ A central question for these surrogates is how to treat the pyrolytic lignin (PL) fraction, which is abundant in real bio-oil but remains thermodynamically poorly characterized.^24,26^ Omitting PL has been reported to influence vapor–liquid equilibrium predictions,^27,28^ whereas its effect on liquid–liquid equilibrium (LLE) is compound-dependent, and the partition coefficients of individual species can be predicted accurately without an explicit PL surrogate.^29,30^ Well-defined synthetic mixtures, therefore, provide the partition data and the model validation that process design requires. On this basis, three models at two levels of prediction are available. The first is the Perturbed-Chain Statistical Associating Fluid Theory (PC-SAFT) equation of state,^31,32^ which describes the associating and polar compounds of bio-oil but needs experimental data to fit its binary interaction parameters.^21,33,34^ The other two are fully predictive: COSMO-RS and the group-contribution Modified UNIFAC 2.0 model (UNIFAC: Universal Functional-group Activity Coefficients).^35,36^

This study investigates the extraction of representative methoxyphenols (guaiacol and 2,6-DMP) from a seven-component SynBO using biobased solvents identified through a systematic in silico screening. The screening strategy integrates thermodynamic predictions from COSMO-RS with sustainability criteria from the CHEM21 guide, formalized through a multicriteria decision analysis (TOPSIS) with explicit weight documentation and Monte Carlo robustness testing, to select environmentally responsible extraction solvents. A primary objective is to obtain experimental LLE data to evaluate the performance of the three thermodynamic models described above. Furthermore, the ternary phase behavior of real bio-oil is experimentally assessed to validate the representativeness of the synthetic surrogate and to identify operational constraints, such as PL precipitation, which may affect the industrial implementation. The resulting thermodynamic parameters and validated models will serve as predictive tools for simulating extraction performance and guiding solvent selection in the separation process design. While the solvents dimethyl carbonate (DMC), cyclopentyl methyl ether (CPME), and 2-methyltetrahydrofuran (2-MeTHF) were initially identified in our prior screening,^37^ the present work extends that preliminary assessment in three key directions: (i) a formal multicriteria ranking with robustness analysis replaces the earlier ad hoc selection; (ii) modified UNIFAC 2.0 ^36^ is evaluated for the first time in LLE systems with more than three components; and (iii) the transferability of fitted PC-SAFT binary interaction parameters is systematically validated for the seven-component SynBO mixture, whose representativeness for real bio-oil is confirmed through ternary phase-diagram comparison. By providing experimental insights, comprehensive model comparison, and validation against real bio-oil systems, this study advances the targeted recovery of lignin-derived phenolics and supports the integration of bio-oil fractionation into sustainable chemical manufacturing.

## 2 Experimental Details

### 2.1 Materials

All the chemicals used in this work are shown in Table 1, with their respective registry number, supplier, and reported mass purities. All of them were used without an additional purification step. The bio-oil used in this study was purchased from BTG (the Netherlands) and stored at 278.15 K (5 °C). Before each use, the bio-oil was thoroughly homogenized by manual shaking.

**Table 1:** Chemicals Used in This Work^a^

Compound	Alias	CAS	Supplier	Purity [wt %]
Water	n/a	7732-18-5	Sigma-Aldrich	HPLC grade^1^
2,6-Dimethoxyphenol	2,6-DMP	91-10-1	Sigma-Aldrich	≥99
Guaiacol	n/a	90-05-1	Sigma-Aldrich	≥99
Furfural	n/a	98-01-1	Sigma-Aldrich	≥99
Acetic acid	n/a	64-19-7	Sigma-Aldrich	≥99
Acetol	n/a	116-09-6	Sigma-Aldrich	≥90
D-Fructose	n/a	57-48-7	Sigma-Aldrich	≥99
Dimethyl carbonate	DMC	616-38-6	Sigma-Aldrich	≥99
Cyclopentyl methyl ether	CPME	5614-37-9	Sigma-Aldrich	≥99.90
2-Methyltetrahydrofuran	2-MeTHF	96-47-9	Sigma-Aldrich	≥90
2-Butanol	2-BuOH	78-92-2	Sigma-Aldrich	≥99

a Reported by the supplier.

### 2.2 Synthetic Bio-Oil Preparation

The SynBO was prepared by mixing representative compounds from the main chemical families of bio-oil in proportions adapted from the literature:^26^ 30 wt % of water, 8 wt % of 2,6-DMP, 12 wt % of guaiacol, 10 wt % of furfural, 10 wt % of acetic acid, 16 wt % of acetol, and 14 wt % of d-fructose. d-Fructose was included to represent the water-soluble carbohydrate fraction of fast-pyrolysis bio-oil, which comprises levoglucosan and other anhydrosugars together with monosaccharides such as glucose and xylose, each present at low individual levels but together forming a carbohydrate fraction comparable to the 14 wt % adopted here.^26,38^ Levoglucosan is the most characteristic of these anhydrosugars; d-fructose, which is itself reported among the sugars of fast-pyrolysis bio-oil,^38^ was adopted as a strongly hydrophilic representative of this lumped fraction owing to its high commercial availability. Chemicals were weighed on an analytical balance (Sartorius Practum 224–1S, Germany) with an accuracy of 0.1 mg. Then, this mixture was agitated in a vortex mixer for 1 min and sonicated by using an ultrasound bath (BIOBASE Ultrasonic Cleaner UC-100A, China) at 313.15 K for 10 min. Finally, the resulting stable dark-yellow mixture was stored in a glass bottle at 278.15 K in the absence of light.

### 2.3 Ternary Phase Diagrams for Biphasic Region Identification

Ternary phase diagrams were constructed to identify the regions where phase separation occurs upon the addition of solvent or water to SynBO and bio-oil, following the procedure described by Han et al. (2020).^25^ SynBO (or bio-oil), solvent, and water were gravimetrically mixed in various proportions to map the feasible composition region. Experimental values are shown in Tables S11–S14. The water content of bio-oil and all solvents employed was measured via the Karl Fischer method prior to diagram construction. Each system was vigorously agitated using a vortex shaker for 5 min. Subsequently, the centrifuge tubes were placed in a digital dry bath (LBX Instruments HBD120, ±0.5 K) and allowed to equilibrate for at least 12 h at a constant temperature of 313.15 K. After equilibration, each system was visually inspected to verify its homogeneous or heterogeneous condition. In the case of bio-oil, the presence of a solid or semisolid phase was carefully examined, as pyrolytic lignin tends to precipitate upon the addition of water or other solvents.^39,40^ Images reported by Han et al.^25^ were used as a reference to identify lignin precipitation. Each feed composition was then categorized as a one-phase system, a two-phase system without PL precipitation, or a two-phase system with PL precipitation. This procedure was repeated for all water/SynBO/solvent and water/bio-oil/solvent mixtures. The ternary diagrams were constructed on a water-free basis for SynBO (or bio-oil) and solvent, while the water axis represents the total water content of the system, comprising water provided by SynBO (or bio-oil), solvents, and any externally added water.

### 2.4 LLE Experiments

LLE experiments were conducted using centrifuge tubes with known feed compositions for each compound. These experiments were performed for ternary (water + solute + solvent) and multicomponent (water + SynBO + solvent) systems at a temperature of 313.15 K and atmospheric pressure. This temperature was kept moderate to avoid promoting the thermal polymerization of the reactive bio-oil oxygenates, a process reported to intensify on heating.^2^ Because these conditions are mild, with a weak carboxylic acid, a moderate temperature, and no added catalyst, the solvents are expected to retain their integrity over the contact time: CPME and 2-MeTHF are recognized for their stability in such media, the latter being protected by its low miscibility with water,^41,42^ and dialkyl carbonates such as DMC undergo only slow hydrolysis under weak-acid conditions at moderate temperature.^43,44^ All compounds were weighed using an analytical balance (Sartorius Practum mod. 224–1S) with an accuracy of 0.1 mg. Then, tubes were manually rotated 180° around a hundred times about their transverse axis for 5 min and placed into a controlled temperature orbital shaker (Eppendorf ThermoMixer C). The mixtures were agitated by orbital motion for 4 h and allowed to settle for at least an additional 14 h. Throughout the entire process, the temperature was kept constant. After the biphasic system was clearly identified, 300 μL of solvent-rich phase and 100 μL of water-rich phase were withdrawn, weighed, and diluted with a known mass of methanol prior to analysis. All samples were prepared in triplicate.

The solvents and all solutes present in SynBO, except d-fructose, were quantified by gas chromatography (Shimadzu Nexis mod. GC-2030) equipped with a flame ionization detector, a split injector, and a DB-WAX UI capillary column (30 m × 0.250 mm × 0.25 μm). Samples of 1 μL were injected at 523.15 K with nitrogen as the carrier gas (13.4 cm^3^·min^–1^, split ratio 10:1). The oven program was: 310.15 K for 3 min, ramp at 10 K·min^–1^ to 523.15 K, and hold for 5 min.

d-fructose was quantified using the Fehling method.^45^ Equal volumes of Fehling A and B reagents were mixed and preheated at 353.15 K for 10 min. For aqueous samples, 200 μL of Fehling mixture was added to 200 μL of undiluted sample, held at 353.15 K for 5 min, then centrifuged for 3 min at room temperature (Allsheng Mini Centrifuge mod. Mini-10 K) to separate the reddish precipitate (Cu_2_O). The supernatant’s absorbance was measured at 670 nm using a microplate reader (Allsheng mod. FlexA-200). Blanks were prepared with water, following the same procedure. For organic samples, 200 μL was evaporated under nitrogen at 323.15 K for 30 min, reconstituted with 200 μL distilled water, and homogenized before Fehling analysis. In the few cases where a residual solvent-rich layer persisted after this reconstitution step, only the aqueous layer was sampled for the colorimetric reading; because d-fructose partitions almost entirely into the water-rich phase (*D* < 0.06), its residual amount in the solvent-rich phase, and hence its effect on the organic-phase composition, is negligible. Furfural and acetol also react with Fehling reagents; their contributions were corrected using independent calibration curves.

## 3 Computational Details

### 3.1 Thermodynamic Models

#### 3.1.1 COSMO-RS

The Conductor-like Screening Model for Real Solvents (COSMO-RS) is a continuum solvation model based on quantum chemical methods with a statistical thermodynamic basis that predicts the properties of pure fluids and liquid mixtures in thermodynamic equilibrium.^46–48^ This method employs a virtual conductor with infinite dielectric constant (ε → *∞*) as the reference state for molecules in the liquid phase.^49^ In this framework, each molecule is embedded in a perfect conductor that completely screens all electrostatic interactions at the molecular surface. The quantum chemical COSMO calculation yields the screening charge density σ on the molecular surface, which arises from the conductor’s response to the solute’s electron density and nuclear charges. This conductor reference state provides a clean decoupling of intermolecular interactions, as molecules virtually “swimming” in a conductor do not interact electrically with each other.^47^

The σ-profile, denoted as *p*_*X*_(σ), represents the probability distribution of the screening charge density σ over the molecular surface of compound *X*.^20,50^ Mathematically, it corresponds to a histogram of surface segments classified according to their screening charge density

1 $$p_{X}\left(\sigma \right)=\frac{A_{X}\left(\sigma \right)}{A_{X}}$$

where *A*_*X*_(σ) is the surface area with charge density σ and *A*_*X*_ is the total molecular surface area. The σ-profile serves as a molecular fingerprint that encodes all relevant information about the polarity distribution of a molecule. Three characteristic regions are typically identified: a hydrogen bond donor region (σ < – 0.0082 e Å^–2^), a nonpolar region (−0.0082 < σ < + 0.0082 e Å^–2^), and a hydrogen bond acceptor region (σ > + 0.0082 e Å^–2^).

The chemical potential of a solute *X* in solvent *S* (μ_*X*_^*S*^) is then calculated by integrating the σ-potential over the solute’s σ-profile^51^

2 $$\mu_{X}^{S}=\int p_{X}\left(\sigma \right)\mu_{S}\left(\sigma \right)d\sigma +\mu_{comb,X}^{S}$$

where μ_comb,*X*_^*S*^ is the combinatorial contribution, which accounts for molecular size and shape differences between solute and solvent molecules.

The LLE compositions are determined iteratively, subject to mass conservation constraints. The procedure is initialized with estimated composition values at a predefined temperature, which are used to calculate the chemical potentials in each phase. Updated mole fractions are then generated using the mole fraction-based distribution coefficient, *D*_*i*_^x^

3 $$D_{i}^{x}=\exp\left(\frac{\mu_{i}^{I}-\mu_{i}^{II}}{RT}\right)$$

4 $$x_{i}^{D}=\frac{D_{i}^{x}}{1+D_{i}^{x}}$$

where μ_*i*_^I^ and μ_*i*_^II^ are the chemical potentials of component *i* in the water-rich (I) and solvent-rich (II) phases, respectively, and *x*_*i*_^*D*^ is the calculated mole fraction of *i* in the water-rich phase. This process iterates until the difference in composition between consecutive steps falls below a convergence threshold, typically 1 × 10^–5^.

#### 3.1.2 PC-SAFT

PC-SAFT (perturbed-chain SAFT) is an equation of state introduced by Gross and Sadowski in 2001 as an alternative to the original SAFT formulation by Chapman et al.^31,32,52^ In particular, it links macroscopic properties and phase behavior to molecular size, shape, intermolecular forces, and association effects. PC-SAFT has demonstrated strong predictive performance across diverse systems such as long-chain molecules,^53^ ionic liquids,^54^ low-molecular-weight compounds,^55^ and even bio-oil components.^21,34^ Consequently, it is widely used to model thermodynamic properties, especially phase equilibria. PC-SAFT adopts a hard-chain reference fluid in which molecules are modeled as chains of equal-sized, covalently bound segments. This representation is coupled to a dispersion term that explicitly captures attractive interactions between chains and accounts for molecular nonsphericity.^56^ Each contribution enters the residual molar Helmholtz energy, *a* ^res^. This residual Helmholtz energy is defined as the difference between the real-fluid Helmholtz energy and that of an ideal gas at identical conditions, and it is expressed as a sum of independent perturbative terms relative to the reference system

5 $$a^{res}=a-a^{ideal}=a^{hc}+a^{disp}+a^{assoc}$$

where *a* ^hc^ represents the hard chain repulsion of the reference system, *a* ^disp^ accounts for the dispersive interactions between chain segments, including the nonspherical shape of the molecules, Finally, *a* ^assoc^ reflects the special self-association interactions^57,58^ that are used in the original PC-SAFT model.^31^ A more rigorous definition of each of these contributions for mixtures can be found in the original PC-SAFT articles.^31,32,52^ The pure component parameters were regressed from vapor pressure and liquid density data or directly retrieved from the literature. Within this framework, each component is characterized by three pure-component parameters, namely, the number of segments per chain *m*_*i*_^seg^, the segment diameter σ_*i*_, and the segment dispersion energy ε_*i*_/*k*_*B*_; associating components additionally require the association volume κ^*AB*^, the association energy ε^*AB*^/*k*_*B*_, and the number of positive/negative association sites *N*_assoc_. Table 2 shows all these PC-SAFT parameters used in this work, together with the molar mass *M*_w_ of each component.

**Table 2:** PC-SAFT Pure-component Parameters Used in This work. *N*_assoc_ Denotes the Number of Positive/Negative Association Sites for Each Component^a^

Compound	***M***_**w**_	*m*_*i*_^seg^	**σ**_***i***_	**ε**_***i***_ **/** *k*_***B***_	***N***_**assoc**_	**κ** ^***AB***^	**ε** ^***AB***^ **/** *k*_***B***_	Source
	**[g·mol** ^**–1**^ **]**		**[Å]**	**[K]**			**[K]**	
Water	18.02	1.2047	σ(*T*)^a^	353.945	1:1	0.04509	2425.67	^59^
2,6-DMP	154.16	4.6300	3.4334	254.493	1:3	0.07291	2133.51	This work
Guaiacol	124.14	3.0024	3.7700	338.277	1:2	0.00950	1472.73	^34^
Furfural	96.08	3.0711	3.3558	320.070	1:1	0.01000	0.00	^60^
Acetic acid	60.05	1.3403	3.8582	211.590	1:1	0.07555	3044.40	^32^
Acetol	74.04	1.4590	3.9020	181.074	1:2	0.03035	3457.12	This work
D-fructose	180.16	7.3870	2.8490	237.190	5:5	0.10000	5000.00	^55^
DMC	90.08	3.2181	3.2581	256.230	1:1	0.01000	0.00	^61^
CPME	100.16	3.0632	3.6919	266.879	1:1	0.01000	0.00	This work
2-MeTHF	86.13	2.6150	3.7029	273.923	1:1	0.01000	0.00	This work

a Temperature-dependent segment diameter for water: σ­(*T*/*K*) = 2.7927 + 10.11 exp­(−0.01775 *T*) − 1.417 exp­(−0.01146 *T*).

For mixtures, the Berthelot–Lorentz mixing rules are used for the interactions of mixed solutions between two components *i* and *j* (for example, water and solvent), which are described as follows:

6 $$\sigma_{ij}=\frac{1}{2}\left(\sigma_{i}+\sigma_{j}\right)$$

7 $$\varepsilon_{ij}=\sqrt{\varepsilon_{i}\varepsilon_{j}}\left(1-k_{ij}\right)$$

where *k*_*ij*_ is a binary interaction parameter used to correct deviations from the geometric mixing rule for the dispersion energy. Table 3 summarizes all binary interaction parameters used in this work and the corresponding experimental properties used as targets for their fitting, primarily LLE data.

**Table 3:** PC-SAFT Binary Interaction Parameters, *k*_*ij*_ = *k*_*ij*,*a*_+*k*_*ij*,*T*_(*T*/K-298.15), Used in This Work. Binary Pairs Not Listed Were Set to *k*_*ij*_ = 0^a^ ^,^ ^b^

Pair	*k*_*ij*,*a*_	*k*_*ij*,*T*_	Fitting data	*T* **-range/K**
Water +2,6-DMP	0.0133	0	Ternary LLE	313
Water + Guaiacol	0.0391	1.474 × 10^–4^	Binary LLE	298–323
Water + Furfural	0.0022	9.134 × 10^–5^	Binary LLE	298–350
Water + Acetic acid	–0.0551	0	Ternary LLE	313
Water + Acetol	–0.0955	0	Ternary LLE	313
Water + D-fructose	–0.0555	1.400 × 10^–4^	^a^	263–298
Water + DMC	–0.0354	–1.122 × 10^–4^	Binary LLE	283–334
Water + CPME	–0.0358	1.292 × 10^–3^	Binary LLE	286–343
Water +2-MeTHF	–0.0602	5.269 × 10^–4^	Binary LLE	273–343
Acetic acid + Guaiacol	0.0855	0	Ternary LLE	313
Acetic acid + Furfural	–0.0210	0	Ternary LLE	313
Acetic acid + DMC	0.0342	0	Ternary LLE	313
Acetic acid + CPME	0.0318	0	Ternary LLE	313
Acetic acid +2-MeTHF	0.0230	0	Ternary LLE	313
Acetol + Furfural	–0.0469	0	Ternary LLE	313
Acetol + DMC	0.0051	0	Ternary LLE	313
Acetol +2-MeTHF	0.0093	0	Ternary LLE	313

a Fitted during D-fructose parametrization using aqueous density, solubility, and osmotic coefficient.^55^.

b All LLE-based parameters were fitted by minimizing the residuals of the experimental mass fractions of each component in each coexisting phase.

The pure-component parameters follow the standard PC-SAFT framework and are transferable in the usual way, whereas the binary interaction parameters regressed in this work are specific to the fitted pairs and to their temperature dependence: they were obtained at atmospheric pressure over the temperature ranges listed in Table 3 and are therefore recommended for use within the investigated composition, temperature (about ±20 K of the fitted range), and pressure window.

#### 3.1.3 Modified UNIFAC 2.0

Liquid-phase activity coefficients for ternary and multicomponent systems were predicted using Modified UNIFAC 2.0,^36^ a group-contribution method that integrates matrix completion methods into the Dortmund-modified UNIFAC framework.^62^

In this version, the UNIFAC pair-interaction parameters Ψ_mn_ between main groups *m* and *n* follow:

8 $$\Psi_{mn}\left(T/K\right)=\exp\left(-\frac{a_{mn}+b_{mn}T}{T}\right)$$

where *a*_mn_ and *b*_mn_ are the pair interaction parameters. Distinct from previous versions, these parameters are derived from learned group-specific feature vectors, allowing for the generation of complete parameter matrices that cover all possible group interactions while reducing the overall mean absolute deviation compared with sequential parametrization. The parameter values were obtained directly from the Supporting Information in the original publication. Subgroup parameters *R*_k_ and *Q*_k_ remain unchanged from Dortmund-modified UNIFAC.

Molecular structures were optimally fragmented into their corresponding groups using Ugropy,^63^ yielding the groups listed in Table 4. Calculations were performed with the implementation from the Thermo^64^ library for the Python programming language. Phase stability and LLE calculations were implemented separately using the tangent plane distance criterion and the isothermal flash, following the methods outlined by Michelsen and Møllerup.^65^

**Table 4:** Dortmund-UNIFAC Group Decomposition of Compounds Used in This Work

Component	Subgroup assignments
Water	H_2_O (1)
CPME	CH_3_ (1), CHO (1), CY-CH_2_ (4)
DMC	(CH_3_)_2_CB (1)
2-MeTHF	CH_3_ (1), CY-CH_2_ (2), CY-CH (1), CY-CH_2_O (1)
D-fructose	CH_2_ (1), OH(P) (1), CY-CH (3), CY-C (1), OH(S) (4), CY-CH_2_O (1)
Furfural	FURFURAL (1)
Guaiacol	ACH (4), AC (1), ACOH (1), CH_3_O (1)
2,6-DMP	ACH (3), AC (2), ACOH (1), CH_3_O (2)
Acetic acid	CH_3_ (1), COOH (1)
Acetol	CH_3_ (1), OH(P) (1), CH_2_CO (1)

### 3.2 Solvent Selection

The computational screening procedure follows the methodology established in our previous work,^37^ starting with a database of more than 1700 solvents. This database comprises only compounds that are liquid at room temperature; species that are solid under ambient conditions were excluded a priori, so that every candidate is a viable extraction solvent, and no unphysical solid state is propagated into the subsequent COSMO-RS calculations. All of the initial calculations were performed using the BP_TZVP_22.ctd parametrization and the COSMObase 2022 database implemented in BIOVIA COSMOtherm 2022 software.

Initially, COSMO-RS was employed to evaluate the miscibility gap between candidate solvents and water at 313.15 K using the “Liquid extraction” module. This approach was selected to simulate the experimental liquid–liquid extraction conditions, where SynBO distributes between water-rich (aq) and solvent-rich (OS) phases.^25^ During this step, the potential solvent loss to the water-rich phase was quantified using eq 9

9 $$solvent\;loss\left(wt\%\right)=\frac{m_{OS}^{aq}}{m_{total}^{aq}}\cdot 100$$

Subsequently, the distribution coefficient at infinite dilution $\left(\log D_{i}^{x,\infty }\right)$ for each “*i*” SynBO component was calculated for immiscible systems at 313.15 K using eq 10

10 $$\log D_{i}^{x,\infty }=\log\left[\exp\left(\frac{-\Delta \mu_{i}^{x,\infty }}{RT}\right)\right]$$

where Δμ_*i*_^x,∞^ = μ_*i*_^x,∞,OS^–μ_*i*_^x,∞,aq^, and log denotes the base-10 (decimal) logarithm. Only solvents yielding $\log D_{i}^{x,\infty }$ > 0 for the key targets (guaiacol and 2,6-DMP) were retained. The combination of the COSMO-RS phase-split criterion at 313.15 K (miscibility gap with water) and the requirement of positive distribution coefficients for the two methoxyphenol targets reduced the initial database of more than 1700 solvents to a curated pool of 156 candidates that are predicted to be immiscible with aqueous phases under the conditions relevant to liquid–liquid extraction. This curated 156-solvent pool, listed in Tables S2–S4 of the Supporting Information, entered a hard-constraint cascade (Section 3.2.1) based on solvent loss, commercial availability, and the CHEM21 SHE profile, whereas the normal boiling point was retained as a downstream-feasibility descriptor evaluated within the multicriteria ranking (*c*_BP_) rather than as a hard filter. The thermodynamic parameters of the surviving solvents were then refined with the BP_TZVPD-FINE_22.ctd parametrization, reserved for this final step because of its high computational cost.^37^

#### 3.2.1 Multicriteria Solvent Ranking

The 156-solvent pool identified in the previous step as immiscible with aqueous phases was further refined through a cascade of five hard constraints. Each entry of this pool was cross-validated for identity through CAS number, canonical name, and SMILES; COSMO-RS descriptors were computed at 313.15 K using the BP-TZVPD-FINE parametrization;^66^ Hansen parameters (δ_*D*_, δ_*P*_, δ_*H*_) were obtained from Hansen^67^ where available and completed by the group-contribution method of Stefanis and Panayiotou^68^ otherwise; and CHEM21 safety, health, and environment (S/H/E) scores plus REACh registration status were transcribed from the Prat 2016 ESI.^69^ All distribution coefficients reported in this section correspond to the COSMO-RS infinite-dilution predictions $\log D_{i}^{x,\infty }$ of the solute in the binary solvent + water system at 313.15 K and are not to be confused with the ternary or multicomponent LLE measurements discussed in Results.

The five hard constraints (C1–C5), their thresholds, and their rationale are summarized in Table 5 and depicted in the selection workflow of Figure 1. They are applied sequentially, a solvent failing any constraint being removed before the next is evaluated: C1 (phase split with water at 313.15 K) defines the aqueous-immiscible pool, C2 caps the COSMO-RS-predicted solvent loss at 15 wt % (a conservative bound relative to the 10 wt % benchmark of Santaella et al.,^70^ padded for COSMO-RS uncertainty in mutual-solubility predictions^66^), C3 requires commercial availability at industrially relevant scales, and C4–C5 remove solvents with a CHEM21 max­(S, H, E) > 7 or a hazardous verdict.^69^ Only candidates surviving the full cascade enter the TOPSIS aggregation (see Figure 2).

**Table 5:** Hard-Constraint Cascade Applied to the 156-Solvent Pool of Candidates Identified as Immiscible with Aqueous Phases at 313.15 K^a^

ID	Criterion	Threshold	Rationale
C1	Phase split with water at 313.15 K (COSMO-RS)	Two liquid phases	A miscibility gap is a prerequisite for liquid–liquid extraction; fully water-miscible solvents are excluded *a priori*.
C2	Solvent loss to the aqueous phase at 313.15 K	≤15 wt %	Upper bound aligned with the 10 wt % benchmark of Santaella et al.,^70^ padded for COSMO-RS uncertainty in mutual solubility predictions.^66^
C3	Commercial availability status	{Verified, Estimated}	Limits the pool to solvents accessible at industrially relevant scales; estimated entries are bulk-quote interpolations validated against >1 t suppliers (Alibaba, ICIS, ChemicalBook).
C4	CHEM21 max(S, H, E) score^69^	≤7	Above this threshold, the CHEM21 guide assigns a hazardous verdict and explicitly recommends substitution.
C5	CHEM21 categorical verdict^69^	≠ Hazardous	Redundant safeguard against database inconsistencies between max(S, H, E) and the verdict (e.g., highly hazardous reassignments by expert panel).

a Constraints are evaluated sequentially; surviving solvents enter the TOPSIS aggregation.

**Figure 1:**
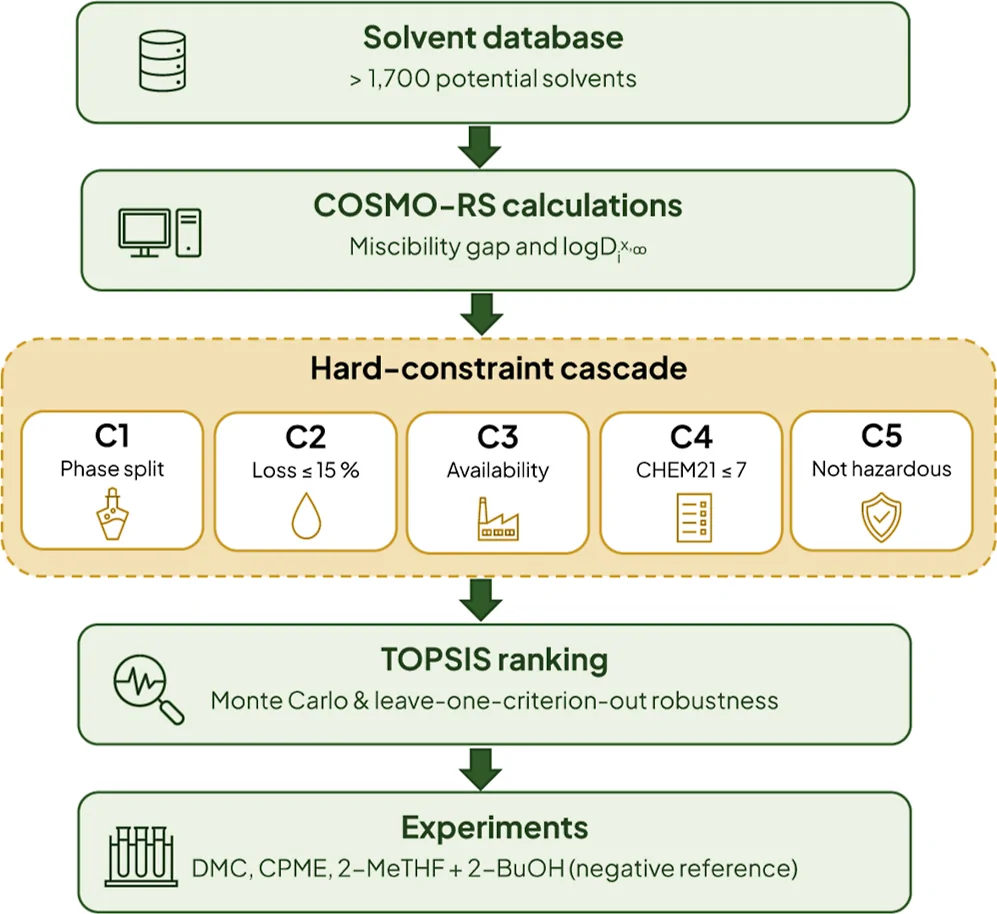
Solvent-selection workflow integrating thermodynamic, SHE, and commercial-availability criteria through a multicriteria (TOPSIS) ranking.

**Figure 2:**
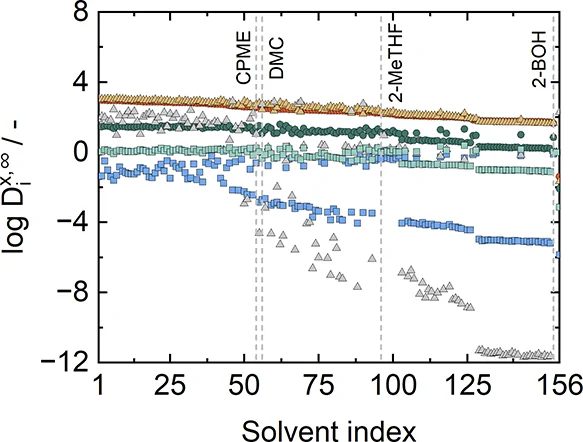
COSMO-RS calculation of infinite dilution distribution coefficients $\left(\log D_{i}^{x,\infty }\right)$ as a function of the solvent index. Vertical dashed lines indicate the four solvents selected for experimental validation: CPME, DMC, 2-MeTHF, and 2-BuOH. Symbols represent solutes: guaiacol ◯ (red ring open), 2,6-DMP Δ (yellow UCDelta,Greek), acetol □ (blue box), furfural ◯ (green ring open), d-fructose Δ (gray), and acetic acid □ (green box).

The surviving pool was then ranked by the Technique for Order of Preference by Similarity to Ideal Solution (TOPSIS), a multicriteria decision analysis (MCDA) method introduced by Hwang and Yoon.^71^ TOPSIS normalizes each criterion across the alternatives, weights them according to user-defined importance, and ranks the alternatives by the relative geometric distance of each candidate to the positive ideal solution (best value on every criterion) and the negative ideal solution (worst value on every criterion); the output is a closeness coefficient between 0 (worst) and 1 (best). TOPSIS was selected over alternative MCDA tools (e.g., AHP, PROMETHEE, VIKOR, SAW) on three grounds explicitly endorsed in the green-solvent selection literature: (i) algorithmic simplicity and full transparency, with no pairwise-comparison or preference-elicitation step that introduces user-dependent inputs;^72^ (ii) compatibility with cardinal numerical criteria of heterogeneous units once normalization is applied, which matches the structure of our criterion set (continuous $\log D_{i}^{x,\infty }$ values, integer CHEM21 scores, and a categorical boiling-point window);^73^ and (iii) precedent in solvent-selection studies for both green-solvent guides^72,73^ and liquid–liquid microextraction screening.^74^ Makwakwa et al.^74^ further reported that TOPSIS and SAW rankings of extraction–solvent pairs were strongly monotonically correlated, indicating that the specific MCDA aggregator is not the dominant source of variability when the criteria and weights are held fixed.

Four normalized criteria entered the TOPSIS aggregation with weights [0.30, 0.30, 0.22, 0.18]: $c_{\log D_{G}^{x,\infty }}$ (capacity toward guaiacol, maximize), $c_{\log D_{DMP}^{x,\infty }}$ (capacity toward 2,6-DMP, maximize), *c*_CHEM21_ = – max­(*S*, *H*, *E*) (SHE penalty, maximize), and *c*_BP_ (distillation-recovery window 70–139 °C per Prat 2016,^69^ scored {0, 0.5, 1}). Bulk price was not used as a quantitative criterion in the aggregation: industrial bulk prices fluctuate substantially with feedstock, region, and time, and treating them on a linear scale alongside ordinal SHE scores would impose a fragile economic axis on a screening intended to highlight thermodynamic and sustainability performance. Bulk price ranges are reported in Table 6 and Supporting Information Table S5 as qualitative industrial context for the candidates retained for further analysis. The weight allocation reflects the *two-target* structure of the application: equal capacity weight is assigned to each methoxyphenol (0.30 + 0.30 = 0.60), consistent with MCDA-based solvent screening practice where dominant criteria receive the largest aggregate share;^72,73^ CHEM21 carries 0.22 to materialize the sustainability dimension as an *active* criterion (not merely a hard-constraint filter, which already excluded hazardous solvents through C5); and the distillation-window term carries the residual 0.18 as a downstream-feasibility tie-breaker among candidates of similar capacity and SHE profile. The MCDA uses $\log D_{i}^{x,\infty }$ values directly rather than selectivity ratios of the form *D*_target_/*D*_reject_: such ratios are sensitive to the arbitrary choice of reject solute and, when expressed as geometric means across multiple rejects, inflate the score when one *D*_reject_ is very small, masking antiselectivity in specific pairs.

**Table 6:** Bio-Based Solvents Retained for Experimental Validation (Upper Block) and Five Conventional Reference Extractants Compared as an External Benchmark (Lower Block)^a^

Solvent	$\log D_{G}^{x,\infty }$	$\log D_{DMP}^{x,\infty }$	*S*	*H*	*E*	Max	Verdict	*T*_b_/°C	**bulk price** ^a^ **/USD kg** ^**–1**^
DMC	2.55	2.72	4	1	3	4	Recommended	90.5	0.58–0.76
CPME	2.53	2.44	7	2	5	7	Problematic	108.7	8.00–15.00
2-MeTHF	2.21	2.20	6	5	3	6	Problematic	80.0	3.00–8.00
2-BuOH	1.64	1.69	3	2	3	3	Recommended	101.6	1.00–1.50
DCM	2.62	3.17	1	7	7	7	Hazardous	39.6	0.20–0.84
CHCl_3_	2.65	3.16	2	7	5	7	Highly hazardous	61.2	0.23–0.87
*n*-Hexane	1.71	1.64	8	7	7	8	Hazardous	68.7	1.01–1.68
Toluene	2.44	2.64	5	6	3	6	Problematic	110.6	0.85–0.99
Et_2_O	2.50	2.44	10	3	7	10	Highly Hazardous	34.6	2.51–5.28

a Bulk prices for industrial-scale supply (>1 t), expressed as USD/kg ranges for the Verified tier. Sources: ChemAnalyst, Intratec, IMARC Group, businessanalytiq, ECHEMI, ICIS, and PriceWatch.ai (Q3 2025-Q1 2026); see Supporting Information Section.

#### 3.2.2 Robustness Analysis, Benchmark, and Complementary HSP Analysis

Robustness of the TOPSIS ranking was probed through two complementary channels. A dual Monte Carlo was run with *N* = 3000 draws perturbing (i) the weight vector by a uniform ±30% and (ii) each $\log D_{i}^{x,\infty }$ value by a Gaussian σ = 0.30, covering the COSMO-RS uncertainty range reported for H-bonded systems.^66^ Concordance between the nominal ranking and the Monte Carlo median ranking was quantified by the Spearman rank correlation coefficient ρ, a nonparametric measure of monotonic association between two rank lists that takes values in [−1, 1]: ρ = 1 indicates identical ranks, ρ = 0 indicates no rank association, and ρ < 0 indicates rank reversal.^75^ By convention, |ρ| is interpreted as very strong above 0.80 and as strong between 0.60 and 0.80;^76^ the closer ρ approaches unity, the smaller the average rank reshuffling induced by the perturbation, and therefore, the smaller the influence of input uncertainty on the final ordering. Leave-one-criterion-out (LOO) analysis was applied to test the sensitivity of the ranking to each individual criterion: each criterion is removed in turn, the remaining weights are renormalized, and the perturbed ranking is compared with the nominal one. A solvent whose rank is bounded within a narrow interval across all single-criterion drops is said to be *not load-bearing* on any individual criterion, an assessment recommended by the MCDA literature to flag rankings driven by a single dominant criterion.^72,77^

In addition to the in-pool ranking, five conventional reference solvents (dichloromethane, chloroform, *n*-hexane, toluene, and diethyl ether) were evaluated under the same COSMO-RS protocol and reported in a reference-only table to allow a head-to-head comparison with the potential solvent candidates on equal computational footing.

As an independent affinity check, Hansen solubility parameter (HSP) distances *R*_*a*_ were computed for the 40-solvent pool using the targets δ_*D*_ = 19.4, δ_*P*_ = 7.8, δ_*H*_ = 12.3 MPa^1/2^ for guaiacol and δ_*D*_ = 19.6, δ_*P*_ = 7.0, δ_*H*_ = 11.7 MPa^1/2^ for 2,6-DMP, estimated via the Hoftyzer–van Krevelen group-contribution method.^78^ Solvents were ranked by ascending *R*_a_ (lower *R*_a_ = greater predicted affinity); no interaction radius *R*_0_ was imposed because the Hansen solubility sphere framework was designed for polymer dissolution, and no experimentally determined *R*_0_ exists for small-molecule phenolic solutes.^67^ The HSP results are reported as a complementary transparency analysis in the Supporting Information section rather than as a criterion in the MCDA; the reasoning is detailed therein and summarized in Section 4.1.1.

## 4 Results and Discussion

### 4.1 In Silico Solvent Screening

Of the more than 1700 solvents in the initial database, 156 were immiscible with aqueous phases at 313.15 K and yielded positive distribution coefficients for guaiacol and 2,6-DMP, defining the curated pool that entered the subsequent screening (Figure 1). Tables S2–S4 in the Supporting Information list these 156 candidates, which encompass a wide variety of chemical classes, with alcohols (17%) and esters (15%) being the most abundant.

Among the screening descriptors, COSMO-RS-predicted solvent loss showed low values for the vast majority of candidates (below 15 wt %, the ceiling later applied as constraint C2). This parameter is a critical factor during downstream process design because the energy required for solvent recovery can be substantial (e.g., in distillation processes), which has relevant consequences for extraction efficiency and process economics.^79,80^ In the literature, a universally defined “acceptable” threshold for this criterion does not exist, as it depends not only on the concentration achieved in the water-rich phase but also on the solvent boiling point, solvent cost, the presence of cosolvents, and other factors.^81^ Additionally, this criterion is aligned with green chemistry principles, which emphasize that solvent loss should be minimized; some studies express this magnitude through the “E-factor” metric for chemical processes.^82^ For instance, Li et al. (2022) reported that the maximum methanol concentration in the solvent-rich phase was 2 v/v % during alkaloid extraction.^81^ Orm et al. (2020) reported losses of neutral vegetable oils ranging from 1.3% to 10.6% into the ethanol/water phase during walnut oil deacidification processes.^83^ Similarly, Pereira et al. (2020) observed comparable solvent loss ranges when investigating the deacidification of Amazonian Pracaxi and Patawa oils using alcoholic solvents in liquid–liquid extraction systems.^84^ Some polyalcohols exhibit similar solvent loss levels during the removal of methoxyphenols from aliphatic phases.^21,37^

These COSMO-RS coefficients are best read as qualitative trends and a relative ranking rather than as absolute values, particularly for polyhydroxylated sugars, whose multiple conformers and extensive intramolecular hydrogen bonding are difficult to capture and lead COSMO-RS to underestimate their aqueous affinity.^29,85^ This limited absolute accuracy explains why fewer candidates than expected are predicted to reject d-fructose, although its strong hydrophilicity still places it, together with acetic acid, among the solutes that are least favorably extracted from the water-rich phase.

These distribution-coefficient trends can be rationalized from the molecular descriptors provided by COSMO-RS, namely, the σ-profile and the σ-potential of each solute (Figure 3). The σ-profile (Figure 3A) places guaiacol and 2,6-DMP predominantly in the nonpolar region with a minor polar contribution attributable to their –OH groups, whereas acetol, d-fructose, and acetic acid display peaks in the polar regions and furfural in the nonpolar region. This distribution explains why d-fructose and acetic acid partition preferentially into polar solvents while the methoxyphenols favor solvents of intermediate polarity. The σ-potential (Figure 3B), where more negative μ­(σ) values indicate stronger affinity for hydrogen-bond donors or acceptors,^86^ shows that guaiacol exhibits the lowest affinity for hydrogen-bond-donor (HBD) solvents and a partial affinity for hydrogen-bond-acceptor (HBA) solvents, with 2,6-DMP displaying the opposite preference; among the remaining solutes, acetol and acetic acid show the highest affinity for HBD and HBA solvents, respectively. These trends are consistent with experimental and molecular-simulation studies of structurally related molecules.^20,85,87–89^

**Figure 3:**
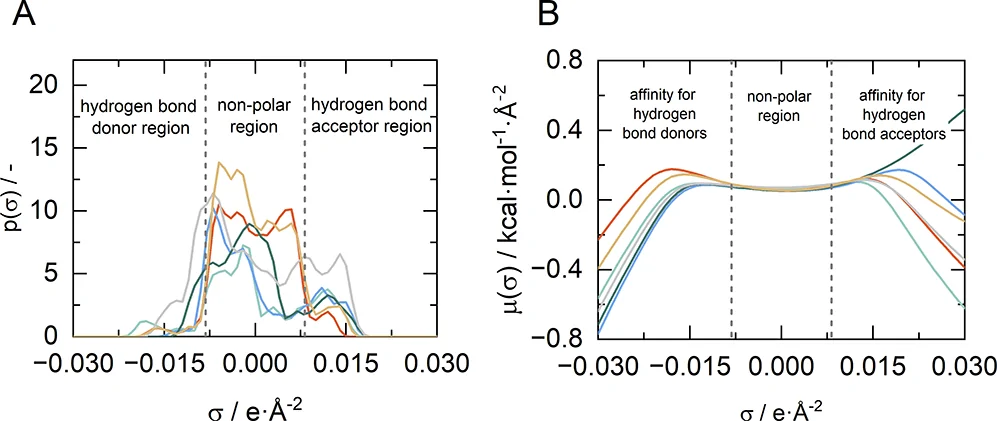
COSMO-RS interaction analysis based on (A) σ-profile and (B) σ-potential of SynBO solutes. Curves represent solutes: guaiacol, (red—)­2,6-DMP (yellow—), acetol (blue—), furfural (green—), d-fructose (gray—), and acetic acid (green—).

Beyond thermodynamic capacity, the CHEM21 solvent selection guide classifies the 156-solvent pool into three categories (recommended, problematic, and hazardous) according to a composite score that integrates safety, health, and environmental factors.^69^ Of the curated candidates, 28% were classified as recommended, 70% as problematic, and 2% as hazardous. The normal boiling point *T*_b_ entered this assessment as a downstream-feasibility descriptor rather than as a hard exclusion criterion: the CHEM21 guide identifies a desirable window of 70 ≤ *T*_b_/°C ≤ 139 that balances volatility-related environmental impacts and the energy demand of solvent recovery by distillation, which can account for up to 80% of the total energy used in the production of bulk chemicals.^69,90,91^ Solvents falling outside this window remain admissible at the screening stage but are penalized in the subsequent multicriteria aggregation through the boiling-point criterion *c*_BP_ (Section 3.2.1), which acts as a tie-breaker among candidates of similar capacity and SHE profile. Scalability and technological readiness should likewise be considered when emerging separation alternatives are evaluated.^13^

In summary, the thermodynamic landscape reveals a clear hierarchy of extraction affinity across SynBO components, with guaiacol and 2,6-DMP consistently favored over the remaining solutes. However, multiple competing factors (thermodynamic capacity, solvent loss, SHE profile, boiling point, and commercial availability) preclude straightforward selection from this landscape alone. To convert the 156-solvent pool into a tractable shortlist, the hard-constraint cascade (Table 5, Figure 1) was applied sequentially, yielding 40 candidates for the multicriteria aggregation; commercial availability (C3) was by far the most restrictive step, and the number of solvents retained and removed at each step is tabulated in Supporting Information Table S1. The following subsections apply the formal multicriteria decision framework to this 40-solvent pool, followed by a benchmark against conventional extractants and a complementary HSP analysis.

#### 4.1.1 Multicriteria Ranking and Robustness

Application of the hard-constraint cascade (C1–C5) to the 156-solvent pool yielded the 40-solvent shortlist that entered the multicriteria aggregation. The TOPSIS ranking of this pool, in which each candidate receives a closeness coefficient between 0 (worst on every criterion) and 1 (best on every criterion), is presented in Figure 4a; the full numerical content of the figure (rank, $\log D_{i}^{x,\infty }$ for each target, S/H/E scores, CHEM21 verdict, normal boiling point, and TOPSIS closeness coefficient for the 40 solvents) is tabulated in Supporting Information Table S7. The ranking is led by a cluster of C_3_–C_5_ esters (ethyl methacrylate, *n*-propyl acetate, isobutyl acetate, *tert*-butyl acetate, isopropyl acetate, and ethyl propionate, occupying ranks 1 to 6) that share a recommended CHEM21 verdict and the highest predicted distribution coefficients in the pool, with closeness coefficients at or above 0.879. The ester cluster is followed by 4-methyl-2-pentanone (MIBK) at rank 7 (closeness 0.867), the only recommended ketone with capacity competitive with the leading esters. DMC follows at rank 8/40 with a closeness coefficient of 0.795. The capacity plane $\log D_{G}^{x,\infty }\times \log D_{DMP}^{x,\infty }$ (Figure 4b), partitioned by the medians of the 40-solvent pool, distributes the candidates very unevenly across four quadrants. Quadrant I (high capacity for both targets, 19 solvents) concentrates the leading C_3_–C_5_ esters, MIBK, and DMC, together with a residual mixture of problematic acrylates, ketones, and aldehydes whose high distribution coefficients are penalized by their CHEM21 max ≥5 score. Quadrant III (low capacity for both targets, 19 solvents) is a heterogeneous group spanning recommended short-chain alcohols, problematic alkanes and cycloalkanes, several ethers with CHEM21 max = 6–7 driven by health or flash-point severity, and aromatic hydrocarbons. Quadrants II and IV are sparsely populated: cyclopentyl methyl ether is the only pool member in IV (high $\log D_{G}^{x,\infty }$ , low $\log D_{DMP}^{x,\infty }$ ), and toluene—an external benchmark, not part of the 40-solvent pool—falls in II (low $\log D_{G}^{x,\infty }$ , high $\log D_{DMP}^{x,\infty }$ ). The fact that most of the pool concentrates along the diagonal connecting Quadrants I and III carries a practical implication for solvent selection: the two methoxyphenol targets are extracted by largely the same chemical families, so a candidate that performs well on one target tends to perform well on the other. The few solvents that fall in the off-diagonal Quadrants II and IV are therefore not typical members of the pool but useful diagnostic points for probing target-specific preference, which becomes relevant when one methoxyphenol must be enriched relative to the other.

**Figure 4:**
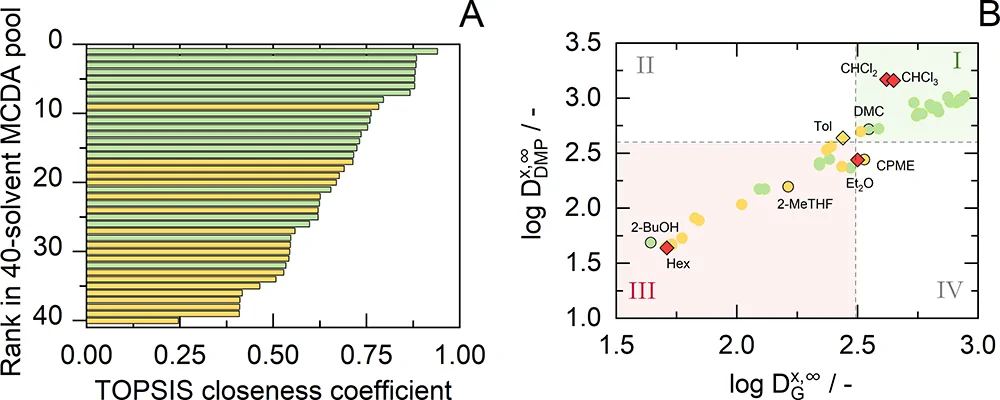
Multicriteria ranking of the 40-solvent MCDA pool. Panel (A) shows the TOPSIS closeness coefficient of each candidate sorted in descending order; panel (B) places the same pool, together with five conventional reference extractants, in the capacity plane $\log D_{G}^{x,\infty }$ versus $\log D_{DMP}^{x,\infty }$ . Markers in both panels are color-coded by CHEM21 verdict^69^ (green box solid) = Recommended, (yellow box solid) = Problematic, red box solid = Hazardous). (A) (black star solid) marks the four candidates retained for experimental validation: DMC (rank 8), CPME (rank 27), 2-MeTHF (rank 30), and 2-BuOH (rank 32). (B)•: 40-solvent MCDA pool; (black diamond solid): five conventional references (Table 6); (black diamond solid): pool medians.

The stability of the ranking under the uncertainty of its inputs was probed with two complementary tests: a dual Monte Carlo perturbation, which simultaneously injects Gaussian noise into the predicted distribution coefficients (standard deviation σ_log *D*_) and uniform variability into the criterion weights (±30%), and a LOO sensitivity, which removes one criterion at a time and reruns the aggregation to assess whether any single criterion alone drives the result. Under σ_log *D*_ = 0.30 and ±30% weight perturbations, the rank-8 candidate (DMC) remained within the top ten in 40% of the 3000 Monte Carlo runs, with the 10th and 90th percentiles of its rank distribution at 5 and 21, respectively; extending the COSMO-RS uncertainty envelope to σ_log *D*_ = 0.50, representative of the upper-bound error reported for H-bonded, carboxylic-acid-containing systems,^66^ shifted its median rank to 14 with the central 50% of its rank distribution lying between 8 and 20. The Spearman rank correlation between the nominal ranking and the Monte Carlo median ranking is ρ = 0.977; values close to unity indicate that the same solvents occupy the same relative positions before and after perturbation, so the aggregation is stable under the uncertainty envelope considered. The LOO analysis (Table S10) bounded the rank of this candidate between 8 and 13 across all four single-criterion drops, meaning that no single criterion alone is responsible for its position; conversely, candidates whose rank shifted by more than five positions when CHEM21 was removed were flagged as primarily SHE-driven and identified as informative diagnostic references for testing the discriminating power of the protocol experimentally. The full Monte Carlo distribution is reported in Tables S8 and S9 of the Supporting Information.

#### 4.1.2 Benchmark against Conventional Solvents and Selection for Experimental Validation

To frame the 40-solvent MCDA pool against the prior art, five conventional extractants commonly reported in the bio-oil fractionation literature (dichloromethane, chloroform, *n*-hexane, toluene, and diethyl ether) were evaluated under the same COSMO-RS protocol and compiled in the lower block of Table 6 as an external reference set. These solvents were not part of the curated 156-candidate pool that entered the cascade and were never considered as biobased alternatives; they are included exclusively to quantify, on equal computational footing, the capacity gap between the biobased candidates retained for ranking and the conventional extractants historically used in the field. As a demonstrative exercise, the same C1–C5 thresholds were applied retrospectively to the five references to make the regulatory and SHE positioning of each solvent explicit: under those thresholds, dichloromethane, chloroform, and diethyl ether would have been removed by C5 (CHEM21 Hazardous or Highly Hazardous verdict);^69^ *n*-hexane would have been removed by C4 (max­(S, H, E) = 8, with the Safety score reflecting its low flash point and the Health score driven by the reproductive-toxicity flag H361, classified as Category 2 under the European Union CLP regulation for carcinogenic, mutagenic, or reprotoxic (CMR) substances) in addition to C5; and only toluene (Problematic, CHEM21 max = 6) would have passed C1–C5 unaltered. Within the table, dichloromethane and chloroform achieve the highest $\log D_{DMP}^{x,\infty }$ (3.17 and 3.16, respectively) but at the cost of CHEM21 verdicts that exclude them from any modern screening exercise (chloroform is upgraded from Problematic to Highly Hazardous in Prat et al. after CHEM21 expert-panel discussion despite sharing max­(S, H, E) = 7 with dichloromethane);^69^ toluene, a widely used extractant for bio-oil phenolics,^25,92^ offers competitive capacity (2.44 and 2.64) with a Problematic CHEM21 profile (*H* = 6); and diethyl ether, a cornerstone of the VTT/Oasmaa bio-oil fractionation scheme^93^ and one of the most widely employed solvents for bio-oil LLE,^94^ carries *S* = 10 (the highest safety score in the set, driven by an extremely low flash point combined with peroxide formation) yet shows $\log D_{i}^{x,\infty }$ values (2.50 and 2.44) comparable to those of typical biobased candidates in the upper region of the MCDA pool. Taken together, these five entries establish a quantitative baseline of capacity that any candidate retained from the MCDA pool must approach to be considered an industrially meaningful alternative; the benchmark therefore frames the trade-off without prescribing the selection.

As an independent sanity check, HSP distances *R*_a_ were computed for the 40-solvent pool to verify that the COSMO-RS-driven ranking does not contradict a simpler, geometric measure of solvent–solute affinity built from group-contribution descriptors. The Spearman correlation between the TOPSIS score and—*R*_*a*,avg_ (the negative of the average HSP distance to guaiacol and 2,6-DMP) is positive but weak (ρ = 0.36, *p* = 0.03), in line with the general expectation that the two frameworks probe overlapping but not identical aspects of affinity.^95,96^ The conventional references DCM and CHCl_3_ show the shortest *R*_*a*_ distances (6.49 and 8.35 MPa^1/2^, respectively), consistent with their high extraction capacity but unfavorable CHEM21 profiles, while *n*-hexane sits at the opposite extreme (*R*_*a*,avg_ = 16.85). Several short-chain alcohols, ethers, and the carbonate ester DMC in the MCDA pool fall within *R*_*a*,avg_ ≤ 10 MPa^1/2^ from the targets, indicating that biobased candidates competitive with the conventional benchmarks also exist on the HSP axis. The full *R*_*a*_ ranking and the HSP values for the targets, estimated by the Hoftyzer–van Krevelen group-contribution method, are reported in Supporting Information Section. Given the weak correlation and the inherent uncertainty of group-contribution HSP estimates for phenolic solutes,^67^ the analysis is used here only as a transparency check and is not propagated into the MCDA aggregation.

Two design choices merit explicit justification before turning to the selection step. First, the screening criteria are not arbitrary: the hard-constraint cascade is anchored in physical and regulatory thresholds (immiscibility, solvent-loss ceiling, commercial availability, and CHEM21 classification), the Monte Carlo weight perturbation quantifies the sensitivity to the chosen weights, and two criteria that were deliberately excluded (bulk price as a quantitative criterion and selectivity ratios) were addressed in Section 3.2.1 and reported only as qualitative context. Second, the benchmark against conventional extractants provides a direct frame of reference for any candidate retained from the MCDA pool under the same COSMO-RS protocol, and the HSP analysis serves as an independent physical sanity check that is explicitly not propagated into the screening, consistent with patterns documented in the solvent-screening literature.^22,70,97^ This multicriteria architecture follows the holistic solvent-selection philosophy advocated by Diorazio et al.,^98^ who demonstrated that combining computational property predictions with regulatory and safety–health–environment (SHE) filters on a pool of 272 solvents yields more robust selections than any single-criterion ranking.

Drawing on the TOPSIS ranking, the robustness analysis, and the benchmark just established, four solvents were retained from the 40-solvent MCDA pool for experimental evaluation: DMC, CPME, 2-MeTHF, and 2-BuOH; their in silico screening profiles are reported in the upper block of Table 6. Two operational constraints shaped the working set. First, only solvents already available in the laboratory at the purities reported in Table 1 could be tested within the planned experimental campaign; this practical limitation is disclosed here for transparency. Second, within that subset, the four candidates were chosen so that they sample distinct regions of the capacity plane introduced in Section 4.1.1, which provides measurable contrast against which the discriminating power of the multicriteria framework can be tested. DMC samples Quadrant I (high capacity for both targets); CPME samples the sparsely populated Quadrant IV (high $\log D_{G}^{x,\infty }$ , low $\log D_{DMP}^{x,\infty }$ ); 2-MeTHF and 2-BuOH both fall in Quadrant III (low capacity for both targets) but carry contrasting CHEM21 verdicts (Problematic and Recommended, respectively), allowing the role of the SHE axis to be probed at a fixed thermodynamic position. All four candidates remain stable under the Monte Carlo perturbation envelope reported in Section 4.1.1 and are supported by independent precedent in the green-solvent literature so that any subsequent observation can be interpreted in light of the screening protocol rather than attributed to chance.

Compared against the conventional baseline of Table 6, DMC (rank 8/40, TOPSIS = 0.795) matches or exceeds the regulatory-compliant references *n*-hexane and toluene on $\log D_{i}^{x,\infty }$ for both target methoxyphenols, while DCM and CHCl_3_ achieve higher $\log D_{DMP}^{x,\infty }$ but are excluded from any modern screening pool by their CHEM21 verdicts. DMC is a nontoxic carbonate ester proposed as a substitute for chlorinated solvents;^99^ its industrial-scale production and bulk-pricing implications are discussed in the industrial-perspective section below. CPME and 2-MeTHF both carry problematic CHEM21 verdicts (CHEM21 max = 7 and 6, respectively) driven primarily by safety and health endpoints rather than by environmental burden; both have been highlighted as biobased alternatives aligned with green chemistry principles, with CPME valued for its stability, low water solubility, and favorable environmental profile relative to traditional ethers,^41^ and 2-MeTHF is derived from lignocellulosic biomass via furfural or levulinic acid pathways.^42^ The two selected ethers and the carbonate ester have been documented as effective extractants of phenolic compounds while leaving sugars in the aqueous phase.^100–105^ 2-BuOH (rank 32/40) was retained as a thermodynamic lower bound: its low predicted $\log D_{i}^{x,\infty }$ for both targets places it in the bottom third of the MCDA pool, while its Recommended CHEM21 verdict and fermentative production routes^106^ make it a meaningful counterpoint to CPME and 2-MeTHF. The safety scores of CPME and 2-MeTHF reflect their low flash points (−1 °C and −11 °C, respectively^69^), which determine their CHEM21 fire-hazard contribution. In a comprehensive review, Usman et al. (2023) documented the use of 2-MeTHF, CPME, and DMC for the extraction of fatty acids, lipids, and oils from solid matrices (solid–liquid extraction);^16^ those applications differ fundamentally from the liquid–liquid extraction approach employed in this work.

### 4.2 Extraction of Value-Added Compounds from SynBO

#### 4.2.1 Thermodynamic Validation for Ternary Systems

Ternary LLE data at 313.15 K (Figures S1–S5 and Tables S15–S34 in the Supporting Information) establish the validation reference for thermodynamic models. System selection followed three hierarchical criteria: (i) abundance of compounds in SynBO, (ii) absence of experimental data in the literature, and (iii) low reaction probability between the solute and solvent under measurement conditions.

Partitioning trends varied significantly among SynBO components: guaiacol and 2,6-DMP concentrated preferentially in the solvent-rich phases (>87 wt %), while d-fructose remained almost exclusively in the water-rich phases (<0.1 wt % in the solvent-rich phase). Acetol and acetic acid are distributed to both phases with near-unity distribution coefficients, as indicated by the nearly horizontal tie-lines. Regarding solvent loss to the aqueous phase, CPME exhibited the lowest values (0.4–1.9 wt %), followed by 2-MeTHF (6.4–17.6 wt %) and DMC (8.3–25.8 wt %), with acetic acid acting as a cosolvent that enhanced solvent solubility in water across all systems. These experimental trends provide the quantitative basis for assessing model predictions in the following section.

Three thermodynamic approaches were evaluated at two distinct levels of predictions. At the purely predictive level, COSMO-RS and modified UNIFAC 2.0 were applied without adjustable parameters, relying exclusively on molecular structure information. At the parametrized level, the PC-SAFT equation of state was employed with binary interaction parameters (*k*_*ij*_) fitted to the experimental ternary data. This distinction is critical for a fair comparison, as the predictive models address the industrially relevant scenario where no prior data are available, while the parametrized PC-SAFT approach leverages fitted binary interactions to enhance the robustness and transferability of multicomponent predictions.

Model performance was quantified using the root-mean-square deviation (RMSD) over the tie-line compositions, as defined in eq 11

11 $$RMSD=\sqrt{\frac{1}{N}\sum_{i=1}^{N}{\left(w_{i}^{\exp}-w_{i}^{calc}\right)}^{2}}$$

where *w*_*i*_^exp^ and *w*_*i*_^calc^ are the experimental and calculated mass fractions, respectively, and N is the total number of data points (tie-lines × phases × components). Note that this composition-based RMSD differs from the metric most often reported for COSMO-RS in the literature, where accuracy is expressed as a deviation in the distribution coefficient (typically 0.2–0.5 log units^37^); the two are not directly comparable, and on the log-coefficient basis, the present predictions lie within that range.

Table 7 summarizes the RMSD values obtained for all of the models. Among the purely predictive models, COSMO-RS achieved a lower overall RMSD (0.0478) than that of Modified UNIFAC 2.0 (0.0749). The PC-SAFT equation of state, benefiting from the fitted binary interaction parameters, achieved the lowest overall RMSD (0.0310).

**Table 7:** RMSD of LLE Calculations for Ternary Systems Composed of Water, Solvent, and Solute at 313.15 K^a^ ^,^ ^b^

**Organic phase** ^b^	Solute	Predictive		Parameterized
		**COSMO-RS**	**modified UNIFAC 2.0**	**PC-SAFT**
Guaiacol	Acetol	0.0441	0.0669	0.0262
	Acetic acid	0.0389	0.0474	0.0202
	2,6-DMP	0.0210	0.0490	0.0285
	D-Fructose	0.0365	0.1199	0.0300
	Average	0.0351	0.0708	0.0262
Furfural	Acetol	0.0639	0.1938^a^	0.0393
	Acetic acid	0.0422	0.1703^a^	0.0283
	2,6-DMP	0.0436	0.0730	0.0287
	d-Fructose	0.0822	0.1077	0.0220
	Average	0.0580	0.1362	0.0296
CPME	Acetol	0.0128	0.0048	0.0080
	Acetic acid	0.0092	0.0314	0.0123
	2,6-DMP	0.0051	0.0839	0.0298
	d-Fructose	0.0029	0.0030	0.0036
	Average	0.0075	0.0308	0.0134
2-MeTHF	Acetol	0.1228	0.0437	0.0733
	Acetic acid	0.0632	0.1466	0.0276
	2,6-DMP	0.0662	0.0627	0.0570
	D-fructose	0.1486	0.0227	0.0085
	Average	0.1002	0.0689	0.0416
DMC	Acetol	0.0387	0.0574	0.0768
	Acetic acid	0.0697	0.0968	0.0266
	2,6-DMP	0.0160	0.0547	0.0474
	d-fructose	0.0292	0.0624	0.0256
	*Average*	0.0384	0.0678	0.0441
Overall		0.0478	0.0749	0.0310

a Based on 2 tie lines.

b Component forming the organic-rich phase in each ternary system (water + solute + organic phase).

Across all 20 systems, COSMO-RS (triangular symbols in Figures S1–S5) reproduced the qualitative tie-line trends and, among the predictive models, gave the lowest overall RMSD (0.0478). Its accuracy was highest for the structurally simple, dispersion-dominated CPME systems (average RMSD 0.0075), where the σ-profile approach performs best, and lowest for acetol and d-fructose, particularly in 2-MeTHF (RMSD 0.1228 and 0.1486; Figure S4), where intramolecular hydrogen bonding and conformational flexibility are not adequately captured. These observations are consistent with the reduced accuracy documented for COSMO-RS on such polar, conformationally flexible solutes,^37^ in line with the experimental validation required when COSMO-RS is used to screen the biphasic dehydration of carbohydrates.^85^

Modified UNIFAC 2.0 provided fully predictive calculations without adjustable parameters, consistent with prior UNIFAC-based work on bio-oil systems.^29,107^ It gave the highest overall deviation (RMSD 0.0749), with the largest errors for furfural systems (average RMSD 0.1362), where the model failed to converge near the plait point and the RMSD was limited to two tie-lines (Table 7, footnote *a*) and for the multifunctional solutes acetol and d-fructose. The advantage of this machine-learning parametrization over the original and Dortmund-modified UNIFAC is one of coverage rather than accuracy: by completing the group-interaction matrix,^36^ it supplies the parameters needed to treat the multifunctional bio-oil constituents studied here, several of which the earlier variants could not describe for lack of tabulated interactions. This broader applicability does not, however, overcome the underlying group-additivity assumption, so for the proximity-effect systems, the accuracy of Modified UNIFAC 2.0 remains below that of COSMO-RS.

The systematic deviations of the group-contribution models for acetol and d-fructose arise from the *proximity effect*:^68,108^ adjacent polar groups, such as the hydroxyl and carbonyl of acetol and the dense intramolecular hydrogen-bond network of the d-fructose furanose ring, violate the assumption that each functional group contributes independently of its molecular environment. Higher-order corrections that mitigate this effect for other properties have not yet been implemented for the activity–coefficient parameters of Modified UNIFAC 2.0, so its LLE predictions for such multifunctional solutes remain semiquantitative at best.

With binary interaction parameters fitted to the ternary data, PC-SAFT achieved the best overall accuracy (RMSD 0.0310) and performed consistently across all solvent groups, with its explicit association terms capturing the hydrogen-bonding interactions of phenolic, carbonyl, and hydroxyl functionalities.^21,60^ The main residual challenge was for carbohydrate structures such as d-fructose, whose nonassociative polar sites (the ring and hemiacetal oxygens) fall outside conventional association schemes. All models reproduced the tie-line slopes of the methoxyphenol and ether systems within reasonable deviations, whereas plait point compositions showed the largest errors (exceeding 15% for the predictive models), a well-documented limitation of activity coefficient models near consolute points.^109^

Overall, model choice depends on data availability and solute complexity: when no experimental data are available, COSMO-RS offers the best balance of accuracy and generality, particularly for ether-based systems; modified UNIFAC 2.0 is a computationally efficient alternative whose accuracy deteriorates for multifunctional solutes where proximity effects violate group additivity; and when data permit parameter regression, PC-SAFT delivers the quantitative accuracy required for rigorous process simulation. These findings provide practical guidance for thermodynamic model selection in the computer-aided design of separation processes for bio-oil upgrading and renewable chemical recovery.

#### 4.2.2 Ternary Phase Diagrams of SynBO and Bio-Oil

TPDs for the water + SynBO + solvent systems at 313.15 K and atmospheric pressure are presented in Figure 5, corresponding to DMC, CPME, 2-MeTHF, and 2-BuOH, respectively, where the experimental data points are classified as single-phase or biphasic systems. Furthermore, a diagonal line that represents the initial water content of SynBO is included in the diagram and delimits the experimental feasible region. A comparative analysis of the SynBO TPDs reveals a similar and extensive miscibility gap for the DMC, CPME, and 2-MeTHF systems (Figure 5.A, 5.B, and 5.C, respectively). The phase envelopes in these three SynBO systems extend deep into the interior of the diagram, reflecting a comparably limited mutual solubility between these organic solvents and water. From an operational perspective, these large and equivalent two-phase regions ensure phase separation across a wide range of feed compositions, suggesting that DMC, CPME, and 2-MeTHF provide similar flexibility for extraction processes when applied to SynBO. In sharp contrast, the SynBO + 2-BuOH system Figure 5.D) displays a constricted biphasic region, where the phase envelope collapses rapidly at moderate alcohol concentrations (≈65 wt %). This reduced immiscibility window constrains the operational flexibility of 2-BuOH as an extractant, as numerous experimental points fall within the single-phase domain. This behavior reflects the high mutual solubility of 2-butanol and water, which would also entail substantial solvent losses to the aqueous phase; together with the low distribution coefficients predicted during the screening, it reinforces the role of 2-butanol as a thermodynamic negative reference. These phase envelopes are specific to 313.15 K since liquid–liquid equilibrium is temperature-dependent; the same protocol could be applied at other practical extraction temperatures, where a higher temperature is generally expected to shrink the two-phase region and hence the operable extraction area as the system approaches its upper critical solution behavior. This trend is illustrated by comparison with Han et al.,^25^ whose 1-butanol/water/bio-oil binodal was measured at 293.15 K and delimits a broad two-phase region, 1-butanol being among their most effective fractionation solvents, whereas the isomeric 2-BuOH studied here at 313.15 K yields a markedly constricted region. The change from a primary to a more water-miscible secondary alcohol and the 20 K higher temperature both act in the same direction, and the narrower gap obtained here is consistent with the expected contraction of the two-phase region as temperature increases.

**Figure 5:**
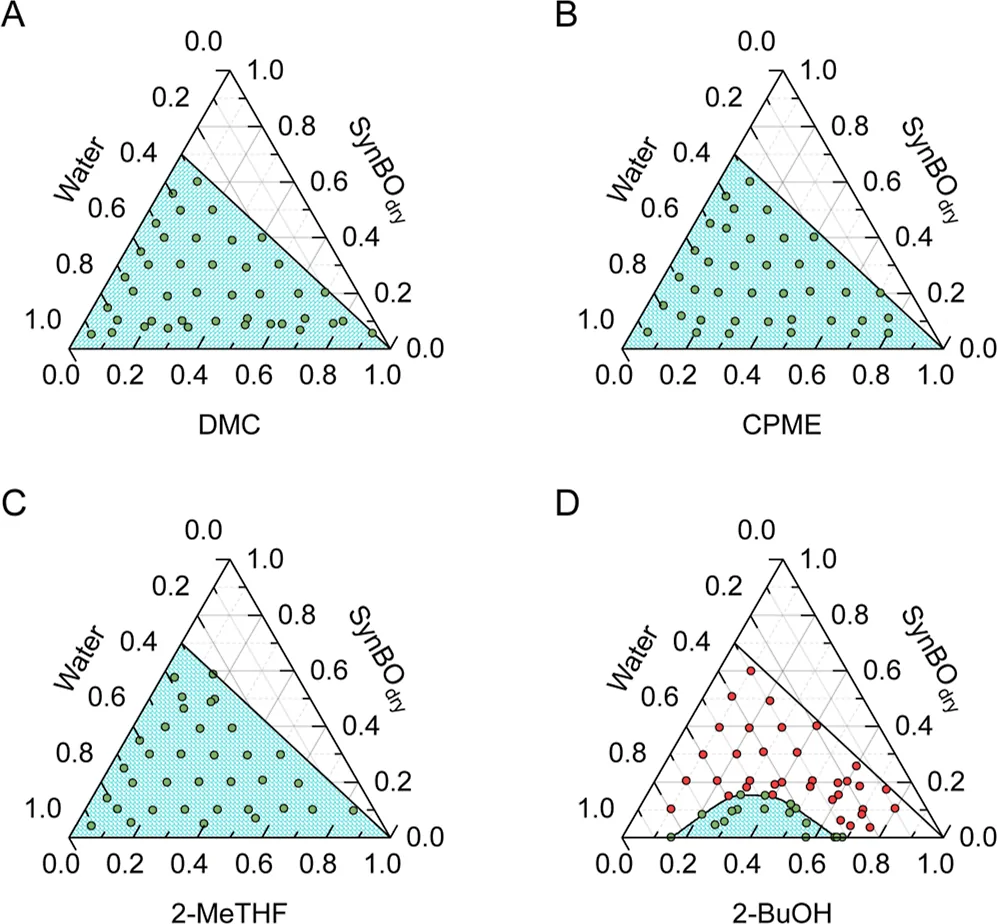
Ternary phase diagrams (mass fraction basis) of SynBO_dry_ with water and different solvents: (A) DMC, (B) CPME, (C) 2-MeTHF, and (D) 2-BuOH at 313.15 K and atmospheric pressure. ◯ (ring open) indicates single-phase formation and ◯ (ring open) indicates two-phase formation. The cyan shingle pattern represents the experimental biphasic region built from data.

To assess the representativeness of SynBO as a model system for real bio-oil extraction, TPDs were also determined experimentally for the water + bio-oil + solvent systems under identical conditions, as shown in Figure 6A–D. In these diagrams, three distinct regions are identified: single-phase zones, biphasic LLE without solid formation, and biphasic systems accompanied by solid precipitation. The precipitate, visually identified as a dark brown solid, is attributed to pyrolytic lignin, consistent with previous reports in the literature.^25,110,111^ High-molecular-mass compounds, such as pyrolytic lignin, can promote intermolecular interactions and solvent–solute aggregation during extraction,^112,113^ potentially modifying the phase equilibria and generating precipitation zones within the TPD.

**Figure 6:**
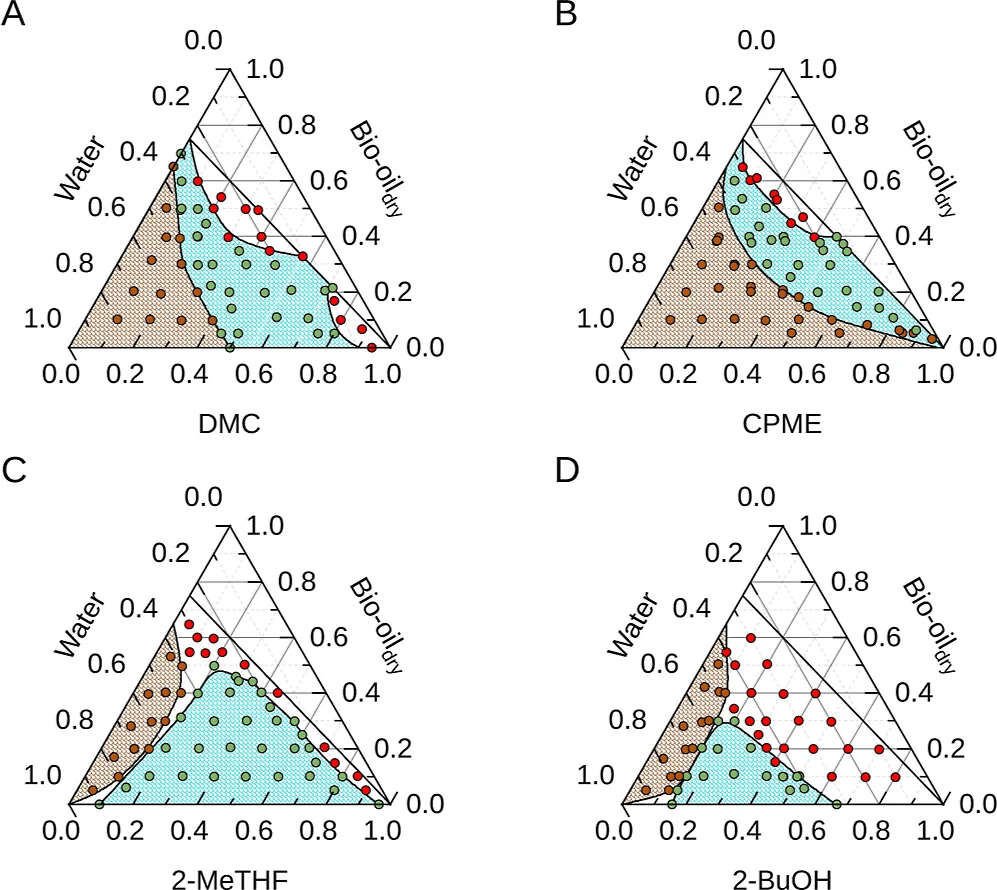
Ternary phase diagrams (mass fraction basis) of bio-oil_dry_ with water and different solvents: (A) DMC, (B) CPME, (C) 2-MeTHF, and (D) 2-BuOH at 313.15 K and atmospheric pressure. ◯ (red ring open) indicates single-phase formation, ◯ (green ring open) indicates LLE, and ◯ (brown ring open) indicates LLE with solid precipitation. The cyan shingle pattern represents the experimental LLE region, while the brown shingle pattern denotes the region where solid precipitation was observed alongside LLE.

A comparative analysis between SynBO and real bio-oil systems reveals both similarities and critical differences. For DMC, CPME, and 2-MeTHF, the overall topology of the phase diagrams is preserved: extensive biphasic regions are observed, confirming that these solvents maintain their capacity for phase separation in complex bio-oil matrixes. However, the presence of pyrolytic lignin and other high-molecular-mass species in real bio-oil generates an additional precipitation zone that encroaches upon the biphasic region, effectively reducing the operational window for clean liquid–liquid extraction. This phenomenon is particularly pronounced in the water-rich corner of the diagrams, where the limited solubility of pyrolytic lignin in aqueous media promotes its precipitation upon the addition of solvent.

Among the four solvents evaluated, 2-MeTHF (Figure 6C) exhibits the largest liquid–liquid region free of solid precipitation, suggesting superior operational flexibility for real bio-oil processing. On the contrary, CPME presents the largest solid precipitation zone at intermediate water contents. DMC presents an intermediate liquid–liquid and precipitation zone, in contrast to the two mentioned solvents. The 2-BuOH system confirms the predictions from the screening stage: the constricted biphasic region observed in SynBO becomes further reduced in real bio-oil, with most of the experimental space occupied by single-phase behavior or solid precipitation. These limitations validate the exclusion of 2-BuOH from subsequent extraction studies and demonstrate that the in silico screening protocol correctly discriminates between effective and ineffective solvents.

A semiquantitative analysis of the TPD data (Tables S11–S14) provides further insight into the extent of PL precipitation across solvents. Of the experimental compositions surveyed for each bio-oil system, the fraction of points exhibiting simultaneous LLE and solid precipitation was 25% for DMC (13/53), 46% for CPME (29/63), 23% for 2-MeTHF (13/57), and 26% for 2-BuOH (14/54). CPME therefore displays a markedly larger precipitation zone, nearly double that of the other three solvents. Importantly, the PL-affected compositions are not randomly distributed: precipitation concentrates at high water content and low solvent content. For DMC, the mean water content of PL-positive points is 55.8 wt % (range 34.9–79.8 wt %), compared with 32.7 wt % for PL-free LLE points; for 2-MeTHF, the pattern is sharper still (mean water 60.0 wt % for PL vs 33.6 wt % for clean LLE), with PL occurring almost exclusively below 15 wt % solvent. These trends are consistent with the well-documented antisolvent behavior of water toward pyrolytic lignin: at high dilution the bio-oil matrix is disrupted and the oligomeric lignin fraction loses its colloidal stability.^111,113^ From an operational standpoint, the solvent-to-feed ratios employed in the multicomponent extractions (25–75 wt % solvent, Section 4.2.3) fall predominantly within the PL-free LLE region of DMC and 2-MeTHF, suggesting that partition coefficients measured with SynBO remain representative of the liquid-phase equilibrium in real bio-oil under practical extraction conditions. For CPME, however, the broader precipitation zone implies that industrial operation may require a pretreatment or settling step to remove PL before solvent recovery. A rigorous correlation between precipitated mass, extraction efficiency, and solvent loss was not attempted because the TPD protocol employed visual phase identification rather than gravimetric quantification of the precipitate; this limitation should be addressed in future work through dedicated precipitation assays with mass-balance closure.

The absence of solid precipitation in SynBO systems, in contrast to real bio-oil, demonstrates that simplified model mixtures enable clearer identification of biphasic liquid regions. While the well-defined composition of SynBO ensures experimental reproducibility and facilitates thermodynamic model validation without interference from undefined high molecular mass fractions, the simplification excludes potential synergistic or antagonistic effects arising from PL that may influence partition coefficients under industrial conditions. It is worth noting that the thermodynamic impact of PL omission depends on the property of interest: while vapor–liquid equilibrium predictions can be more sensitive to PL absence,^27,28^ recent LLE studies have shown that partition coefficients of individual compounds can be adequately predicted without explicit PL representation, even when bulk phase ratios exhibit larger sensitivities to its omission.^29^ This compound-dependent behavior suggests that for extraction applications targeting specific molecules, the SynBO approach captures the relevant thermodynamic features governing solute partitioning. Nevertheless, the qualitative agreement in phase behavior between SynBO and real bio-oil, particularly regarding the relative performance ranking of the candidate solvents, confirms that the simplified model reproduces the essential thermodynamic trends governing extraction. Given the experimental complexity associated with solid precipitation and the compositional variability of real bio-oil, subsequent distribution coefficient measurements and thermodynamic modeling efforts were conducted exclusively with SynBO, providing systematic characterization of solute partitioning behavior while the TPD validation, presented here supports the transferability of these results to real bio-oil systems within the LLE regime. These considerations delimit the applicability of the SynBO surrogate: it is validated for determining and modeling the liquid–liquid partitioning of individual bio-oil solutes at 313.15 K. The multicomponent modeling is performed entirely within the two-phase liquid–liquid region, which for DMC and 2-MeTHF coincides with the 25–75 wt % solvent operating window shown by our real-bio-oil diagrams to lie outside the pyrolytic-lignin precipitation zone; omitting an explicit PL fraction is therefore justified for this purpose. The surrogate is correspondingly not intended to reproduce the overall phase-split ratios or operation in the water-rich, low-solvent region where PL precipitates, most notably for CPME. Three principal limitations of the SynBO surrogate should nonetheless be kept in mind when extrapolating these results. First, the seven-component formulation omits the high-molecular-mass PL fraction, which typically constitutes 15–25 wt % of real bio-oil (with our specific bio-oil at 26.6 wt %, quantified by cold-water precipitation^25^) and can modify both the effective solvent capacity and the hydrodynamic behavior of the extract phase through viscosity increases of up to 20× relative to SynBO.^110^ Second, SynBO does not include minor reactive species (e.g., hydroxyacetaldehyde and levoglucosan) that may participate in competing reactions (most notably aldol condensation and esterification) under industrial residence times, potentially altering the effective distribution coefficients. Third, the controlled water content of SynBO (fixed at 30 wt % by formulation) cannot reproduce the batch-to-batch variability of real bio-oil (typically 20–35 wt % water), which shifts phase envelopes and affects solvent-to-feed ratio requirements. Addressing these gaps will require extraction campaigns with real bio-oil fractions or with PL-spiked surrogates, coupled with gravimetric mass balance closure for both the liquid and solid phases.

#### 4.2.3 Distribution Coefficients and Thermodynamic Modeling

Following the detection of biphasic regions via TPDs for the water + SynBO + organic solvent system at 313.15 K, liquid–liquid extractions were performed to determine the distribution ratios of all SynBO compounds. These extractions were carried out for the DMC, CPME, and 2-MeTHF. The selected extraction points corresponded to solvent concentrations of 25, 50, and 75 wt % relative to the total mixture mass, maintaining a SynBO_*dry*_/water mass ratio of 2:3. The experimental partition coefficients, defined here on a mass-fraction basis as *D* = *w*_OS_/*w*_aq_ (distinct from the mole-fraction-based *D*_*i*_^x^ used in the COSMO-RS iterative procedure, eq 3), for the six representative solutes in the water + SynBO + organic solvent (DMC, CPME, or 2-MeTHF) systems at 313.15 K are presented in Figure 7, while the corresponding RMSD values for the thermodynamic frameworks are summarized in Table 8. Detailed values of multicomponent systems are shown in Table S35.

**Figure 7:**
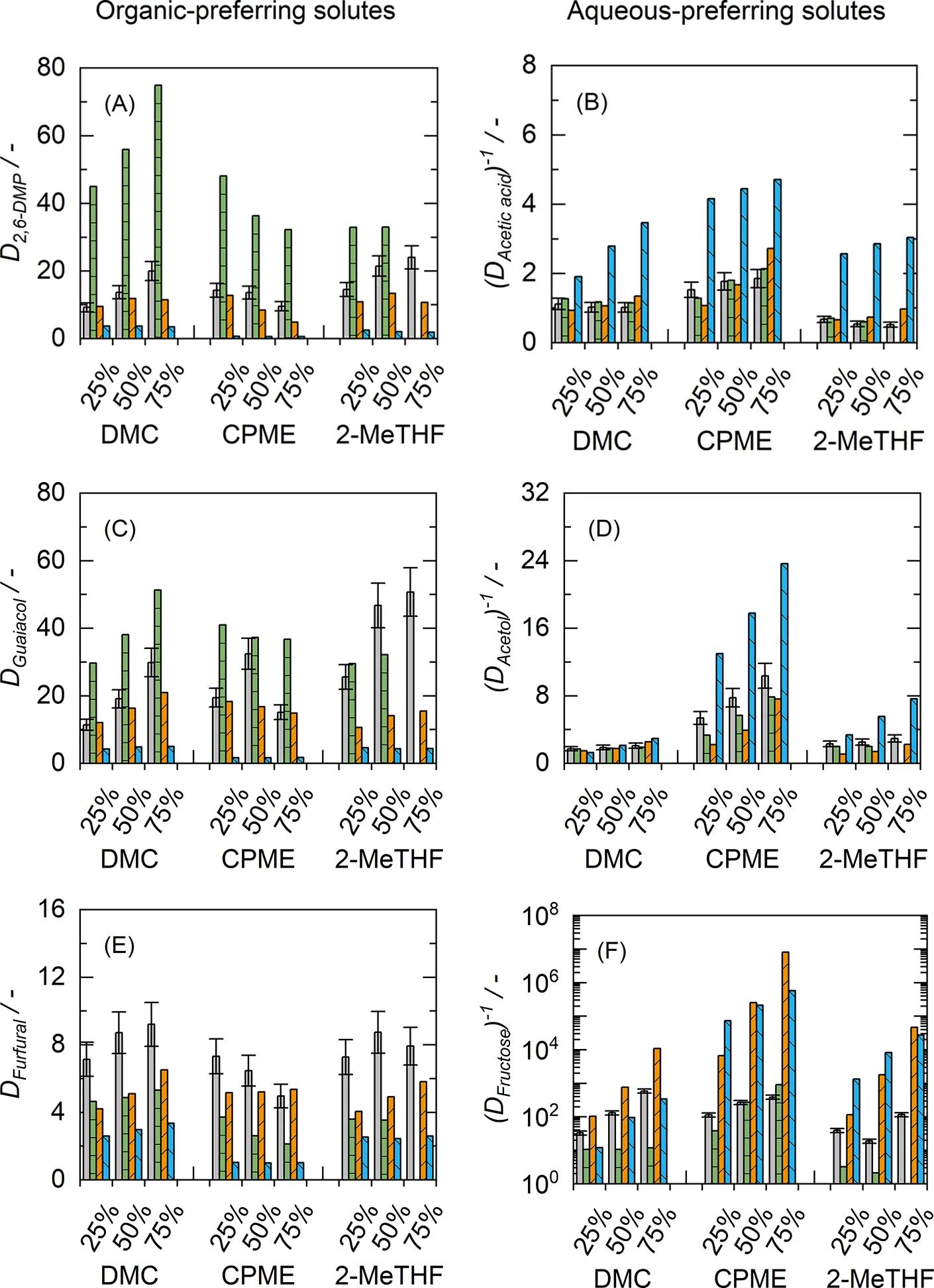
Partition coefficient (*D* = *w*_OS_/*w*_aq_) for various solutes in the SynBO system (dry SynBO/water mass ratio of 2:3) extracted using different organic solvents (OS) at varying mass contents and 313.15 K. The solutes include (A) 2,6-DMP, (B) acetic acid, (C) guaiacol, (D) acetol, (E) furfural, and (F) d-fructose. Experimental data are represented by 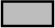 bars, while model predictions are shown for 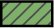 COSMO-RS, 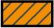 PC-SAFT, and 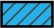 Modified UNIFAC 2.0.

**Table 8:** RMSD for Multicomponent LLE Predictions of the Water + SynBO + Solvent System at 313.15 K

System	Predictive		Parameterized
	**COSMO-RS**	**Modified UNIFAC 2.0**	**PC-SAFT**
DMC −25%	0.0329	0.0397	0.0270
DMC −50%	0.0338	0.0468	0.0273
DMC −75%	0.0472	0.0503	0.0258
*DMC Average*	0.0380	0.0456	0.0267
CPME −25%	0.0298	0.0537	0.0355
CPME −50%	0.0346	0.0395	0.0333
CPME −75%	0.0323	0.0267	0.0287
*CPME Average*	0.0322	0.0399	0.0325
2-MeTHF −25%	0.0275	0.0530	0.0303
2-MeTHF −50%	0.0466	0.0378	0.0218
2-MeTHF −75%	–^a^	0.0226	0.0103
*2-MeTHF Average*	0.0371^b^	0.0378	0.0208
Overall Average	0.0358^b^	0.0411	0.0267

a The COSMO-RS did not converge for this system.

b Average excludes 2-MeTHF −75%.

As illustrated in Figure 7, a hierarchical partitioning was observed, primarily governed by the chemical functionalities of the solutes. Methoxyphenols, namely, 2,6-DMP and guaiacol, exhibited the highest affinity for the solvent-rich phase, with maximum experimental distribution coefficients of *D*_2,6–DMP_ = 23.99 and *D*_Guaiacol_ = 50.73 at 75 wt % solvent content, respectively. Among the thermodynamic models evaluated, the PC-SAFT equation of state provided the most accurate prediction of this behavior, yielding the lowest overall average RMSD (0.0267). This predictive performance can be attributed to the use of optimized binary interaction parameters derived from previously measured ternary systems, which adequately capture the hydrogen-bonding interactions between phenolic hydroxyl groups and the biobased solvents.

In contrast, oxygenated solutes such as acetic acid and acetol exhibited amphiphilic behavior with *D* values near unity (0.10–1.92), indicating a significant presence in both phases. While PC-SAFT (overall RMSD = 0.0267) and COSMO-RS (overall RMSD = 0.0358) captured these trends qualitatively, modified UNIFAC 2.0 showed higher deviations (overall RMSD = 0.0411), particularly at elevated solvent contents. These discrepancies suggest that group-contribution methods have inherent difficulties in accounting for the proximity effects and complex intramolecular interactions present in multifunctional molecules for multicomponent mixtures. For d-fructose, which remained almost exclusively in the water-rich phase (*D*_D–fructose_ < 0.06), all models predicted its low extraction efficiency. However, COSMO-RS failed to converge for the water + SynBO + 2-MeTHF system at 75 wt % solvent content. At such high solvent fractions, the residual aqueous phase becomes dilute, and the chemical potential differences between phases diminish, causing the iterative scheme (eqs 3 and 4) to oscillate rather than converge. This behavior is consistent with the known sensitivity of COSMO-RS flash calculations to asymmetric compositions near the boundary of the two-phase region in multicomponent systems.^114^

The observed partitioning hierarchy can be rationalized through three complementary molecular-level mechanisms. First, the high distribution coefficients of guaiacol and 2,6-DMP arise from strong, directional O–H···O hydrogen bonds between their phenolic hydroxyl groups and the acceptor sites of the ether (CPME, 2-MeTHF) or carbonyl (DMC) solvents, combined with favorable π–π and CH–π dispersion interactions between the aromatic ring and the solvent alkyl backbone. Second, the near-unity *D* values of acetic acid and acetol reflect a competition between two solvation environments: their carboxylic or hydroxyl functionalities form strong H-bonds with water, while their short aliphatic chains interact weakly with the organic phase, producing amphiphilic partitioning that shifts only modestly with solvent content. Third, the effective exclusion of d-fructose (*D* < 0.06) is governed by its polyhydroxylated structure, which establishes an extensive cooperative H-bond network with the surrounding water molecules; the entropic penalty of disrupting this hydration shell far exceeds any enthalpic gain from transfer to the organic phase. This molecular picture, which combines preferential extraction of moderately hydrophobic phenolics, coextraction of small amphiphiles, and rejection of highly hydrophilic sugars, underpins the “thermodynamic sieve” behavior exploited in the screening protocol and is consistent with the σ-profile analysis of COSMO-RS, where the charge-density overlap between solvent and solute surfaces determines the sign and magnitude of the chemical-potential driving force for phase transfer.

Extending the comparison from the ternary systems to the multicomponent SynBO matrix, PC-SAFT retained its accuracy (Table 8), supporting the transferability of its fitted molecular and association parameters, whereas Modified UNIFAC 2.0 again showed the largest deviations for the multifunctional solutes. These magnitudes are consistent with the literature: Liang et al.^115^ reported maximum absolute average deviations of 28.1% for water-rich-phase solubilities when applying PC-SAFT to petroleum fluid + MEG + water systems, yet still recognized it as suitable for such complex mixtures; our previous COSMO-RS predictions for methoxyphenol extraction from aliphatic phases gave root-mean-square errors below 0.41 logarithmic units;^37^ and Rojas Salas et al.^29^ obtained, with UNIFAC Dortmund on a 17-compound fast-pyrolysis bio-oil, mean relative errors of approximately 27% for the organic-to-aqueous mass ratio and 8.7–10.5% for levoglucosan partitioning, with acetic acid consistently underpredicted (∼34%) owing to the difficulty of capturing strong hydrogen bonding. Because the choice of thermodynamic model propagates into the efficiency and economics of downstream separation design,^116^ the combined use of PC-SAFT for rigorous process simulation and COSMO-RS for exploratory solvent assessment, as applied here to the SynBO mixture, is a practical strategy whose continued validation for complex biomass-derived systems remains warranted.^114^

From an industrial perspective, the practical implementation of the proposed extraction scheme depends on solvent cost, availability, and recoverability. At the industrial scale, DMC benefits from established large-volume production (>100 kt/year, 0.58–0.76 USD/kg),^99^ whereas CPME and 2-MeTHF are currently produced at smaller scales, which translates into higher unit costs (8–15 and 3–8 USD/kg, respectively).^41,42^ The bulk-price ranges reported in Table 6 were compiled from industrial sources and market reports; details on the estimation methodology are provided in the Supporting Information section. Solvent recovery via simple distillation is facilitated by the moderate normal boiling points of the three candidates (DMC: 90.5 °C; 2-MeTHF: 80.0 °C; CPME: 108.7 °C), all of which lie within the CHEM21-recommended distillation window (Section 3.2.1) and are well separated from the boiling points of the target methoxyphenols (guaiacol: 205 °C; 2,6-DMP: 261 °C). The low solvent losses observed for CPME in ternary experiments (0.4–1.9 wt %) further support its candidacy for process scale-up, whereas the higher losses of DMC and 2-MeTHF would require optimized recovery stages. The boiling-point gaps between solvent and target methoxyphenols (Δ*T*_b_ > 95 °C in all cases) suggest that single-stage distillation at atmospheric pressure should achieve >95% solvent recovery, as reported for analogous ether–phenol separations.^41^ CPME’s chemical stability toward peroxide formation under anhydrous conditions^41^ and DMC’s thermal stability up to 350 °C^99^ are favorable for multiple recycle loops; however, 2-MeTHF’s susceptibility to ring-opening under strongly acidic conditions (pH < 2)^42^ may limit its reuse in processes involving concentrated acetic acid fractions. Quantitative assessment of solvent degradation rates and recovery yields over multiple extraction–distillation cycles remains an open question that should be addressed experimentally before process scale-up. Advancing these solvents beyond the current technology readiness level will require pilot-scale validation addressing solvent recycling efficiency, long-term chemical stability under process conditions, and the economic trade-off between extraction performance and solvent replacement costs.

## 5 Conclusion

This study applied an in silico, multicriteria strategy to select biobased solvents for the recovery of guaiacol and 2,6-DMP from SynBO. Distribution coefficients predicted by COSMO-RS were combined with the CHEM21 SHE profile and with commercial availability and economic criteria, and these inputs were aggregated through a TOPSIS ranking that was tested by Monte Carlo and leave-one-criterion-out analyses. DMC emerged as a high-ranking candidate (rank 8/40) whose predicted capacity for both targets matched or exceeded the conventional extractants *n*-hexane and toluene, while it retained a recommended CHEM21 verdict. DMC, CPME, and 2-MeTHF were carried forward for experimental validation, and 2-BuOH was included as a thermodynamically negative reference.

Ternary phase diagrams were then measured experimentally to map the biphasic regions of the four solvents in both SynBO and real bio-oil. DMC, CPME, and 2-MeTHF displayed wide miscibility gaps that ensure phase separation over a broad composition range, whereas 2-BuOH showed a constricted two-phase region that collapsed at a moderate alcohol content. This limited phase behavior, together with its low predicted capacity, led to its exclusion from the subsequent extraction experiments, which confirmed its intended role as a negative reference. In real bio-oil, the same diagrams additionally revealed PL precipitation zones that narrowed the operable feed ratios but preserved the qualitative ranking of the solvents.

In the multicomponent SynBO extractions performed with DMC, CPME, and 2-MeTHF, the target methoxyphenols partitioned strongly into the solvent-rich phase, with guaiacol and 2,6-DMP reaching distribution coefficients as high as 50.73 and 23.99, respectively. In contrast, d-fructose was almost entirely rejected to the water-rich phase (*D* < 0.06), while the amphiphilic acetic acid and acetol were distributed between both phases (*D* ≈ 1). The extraction therefore behaved as a thermodynamic sieve that concentrated the value-added methoxyphenols and retained the most hydrophilic sugar in the aqueous stream. Among the three models tested against these data, PC-SAFT reproduced the multicomponent partitioning most accurately (overall RMSD of 0.0267), ahead of the purely predictive COSMO-RS and Modified UNIFAC 2.0, and was therefore the most adequate model for these systems. To the best of our knowledge, this work also provides the first evaluation of Modified UNIFAC 2.0 for multicomponent LLE with more than three components.

Future work incorporating PL, a broader temperature range, and additional methoxyphenols would extend this approach from SynBO to real bio-oil and industrial extraction conditions.

## Supplementary Material



## Notes

Curated 156-solvent list with COSMO-RS distribution coefficients, CHEM21 SHE profiles, and bulk-price ranges; step-by-step reduction of the solvent pool through the hard-constraint cascade; bulk-price estimation methodology; Hansen solubility parameter analysis; TOPSIS-ranked 40-solvent pool with Monte Carlo and leave-one-criterion-out robustness analyses; ternary phase-diagram compositions for the four tested solvents with synthetic and real bio-oil; experimental ternary liquid–liquid equilibrium data for the model validation systems; and experimental and model-predicted (COSMO-RS, Modified UNIFAC 2.0, and PC-SAFT) multicomponent liquid–liquid equilibrium data (PDF)
