# Supplementary material for: Pyrolysis-Oil Methoxyphenol Extraction: From Biobased Solvent Screening to Thermodynamic Modeling
Source: Energy Fuels. 2026 Aug 14;40(34):18970–89. doi: 10.1021/acs.energyfuels.6c02836 (PMC13528198; doi:10.1021/acs.energyfuels.6c02836)
Supplement: Supplementary file 1 [file ef6c02836_si_001.pdf]

Supporting Information: Pyrolysis-oil methoxyphenol extraction: from bio-based solvent screening to thermodynamic modeling

Sebastián Ormazábal-Latorre,† Esteban Cea-Klapp,† Maximilian Fleckenstein,† Nicolás F. Gajardo-Parra,† Ady Giordano,‡ Andrea Sánchez-Monedero,¶ Emilio J. González,¶ María González-Miquel,¶ and Roberto I. Canales*,†

†*Departamento de Ingeniería Química y Bioprocesos, Pontificia Universidad Católica de Chile, Santiago, 7820436, Chile.*

‡*Facultad de Química y de Farmacia, Pontificia Universidad Católica de Chile, Santiago 7820436, Chile.*

¶*Departamento de Ingeniería Química Industrial y del Medio Ambiente, ETSI Industriales, Universidad Politécnica de Madrid, C/ José Gutiérrez Abascal 2, Madrid, 28006, Spain*

E-mail: rocanalesm@uc.cl

Final solvent list

Table S1: Step-by-step reduction of the 156-solvent aqueous-immiscible pool through the hard-constraint cascade (C1–C5), giving the number of solvents retained and removed at each step. C1 is satisfied by construction of the curated pool; the 40 solvents surviving the full cascade enter the TOPSIS aggregation.

Step	Constraint	Survivors	Removed
0	Aqueous-immiscible curated pool	156	n/a
C1	Phase split with water at 313.15 K (COSMO-RS)	156	0
C2	Solvent loss to aqueous phase $\leq$15 wt%	154	2
C3	Commercial status $\in$ {Verified, Estimated}	42	112
C4	CHEM21 max(S, H, E) $\leq$ 7	41	1
C5	CHEM21 verdict $\neq$ Hazardous	40	1

Table S2: *In silico* process results of selected solvents (Part 1 of 3).

Index	Solvent	CAS No.	Solvent loss	$\log D^{\infty}_{i,\mathrm{OS/aq}}$ @ 40 °C						CHEM21				Bulk price[a]
			[wt%]	AcAcid	Gua	Acetol	Furf	D-fruc	2,6-DMP	E	H	S	Cond.	[US$/kg]
1	2-methyl-pentanal	123-15-9	0.77	1.88	2.99	0.01	1.45	−1.39	3.04	7	2	4	Prob.	n.a.
2	2-ethylbutyraldehyde	97-96-1	0.92	2.04	2.97	0.02	1.45	−1.35	3.03	5	5	4	Prob.	n.a.
3	(z)-2-methyl-2-pentenal	623-36-9	2.72	1.98	2.97	0.15	1.49	−0.49	3.03	5	6	3	Prob.	n.a.
4	valeraldehyde	110-62-3	2.10	1.76	2.96	0.10	1.49	−1.01	3.04	3	2	4	Rec.	n.a.
5	hexanal	66-25-1	0.59	1.84	2.96	0.03	1.45	−1.40	3.03	3	2	3	Rec.	n.a.
6	7-oxabicyclo[4,1,0]heptane	286-20-4	1.32	2.44	2.95	−0.08	1.35	−1.61	2.98	3	7	1	Prob.	n.a.
7	isoamylformate	110-45-2	0.50	0.98	2.94	0.04	1.47	−1.32	3.06	5	5	3	Prob.	n.a.
8	n-propylacetate	109-60-4	2.16	2.10	2.94	0.11	1.48	−0.85	3.02	3	2	4	Rec.	1.32–1.51
9	3-methylbutanal	590-86-3	2.54	1.72	2.94	0.09	1.48	−1.08	3.03	7	2	5	Prob.	n.a.
10	n-butylacetate	123-86-4	0.70	2.10	2.94	0.04	1.45	−1.17	3.00	3	2	4	Rec.	0.96–1.76
11	sec-butylacetate	105-46-4	0.85	2.33	2.93	0.04	1.44	−1.21	2.98	5	5	3	Prob.	0.73–1.55
12	aceticacid-2-methylpropylester	110-19-0	0.77	2.08	2.92	0.04	1.44	−1.24	2.99	3	2	4	Rec.	1.20–1.80
13	tert-butylacetate	540-88-5	0.88	2.33	2.91	0.03	1.42	−1.23	2.96	3	2	4	Rec.	2.50–4.00
14	2,2-dimethylpropanal	630-19-3	3.06	1.76	2.90	0.07	1.46	−1.12	2.98	3	3	5	Rec.	n.a.
15	n-propylformate	110-74-7	3.50	1.04	2.89	0.15	1.50	−0.87	3.04	5	6	5	Prob.	n.a.
16	isopropylacetate	108-21-4	2.57	2.02	2.88	0.11	1.46	−0.94	2.97	3	2	4	Rec.	0.80–1.10
17	ethylpropionate	105-37-3	1.95	2.13	2.88	0.10	1.47	−1.07	2.97	3	2	4	Rec.	1.50–2.50
18	propylpropionate	106-36-5	0.58	2.24	2.88	0.02	1.43	−1.40	2.94	3	2	3	Rec.	n.a.
19	ethylbutyrate	105-54-4	0.55	2.15	2.88	0.01	1.41	−1.44	2.95	3	2	3	Rec.	n.a.
20	diethylcarbonate	105-58-8	1.28	1.72	2.88	0.13	1.50	−1.18	3.01	3	2	3	Rec.	0.90–1.50
21	2-hexanone	591-78-6	1.59	1.97	2.87	0.10	1.45	−0.89	2.93	5	5	3	Prob.	n.a.
22	dimethyl$_{a}dipate$	627-93-0	2.28	1.23	2.87	0.22	1.51	−0.68	2.99	7	2	1	Prob.	n.a.
23	3-hexanone	589-38-8	1.54	2.26	2.86	0.10	1.48	−1.08	2.91	5	5	3	Prob.	n.a.
24	tert-butylformate	762-75-4	5.56	1.47	2.85	0.20	1.50	−0.49	2.95	5	6	5	Prob.	n.a.
25	2-methylpropanoicacidethylester	97-62-1	0.68	2.20	2.85	0.02	1.42	−1.48	2.92	3	2	1	Rec.	n.a.
26	3-methyl-2-pentanone	565-61-7	3.24	1.95	2.85	0.11	1.44	−0.64	2.88	3	1	4	Rec.	n.a.
27	2-methyl-3-pentanone	565-69-5	2.48	2.15	2.85	0.10	1.45	−0.82	2.88	5	5	4	Prob.	n.a.
28	aceticacid-2-propenylester	591-87-7	2.30	1.35	2.84	0.18	1.50	−0.92	2.99	3	9	4	Haz.	n.a.
29	4-methyl-2-pentanone	108-10-1	2.94	1.82	2.84	0.11	1.44	−0.69	2.88	3	2	4	Rec.	1.16–1.98
30	2-methylpropanoicacidmethylester	547-63-7	2.22	1.95	2.83	0.08	1.45	−1.17	2.93	3	2	4	Rec.	n.a.
31	2-propenoicacid-2-methylpropylester	106-63-8	0.23	1.65	2.83	−0.00	1.41	−1.52	2.92	5	2	3	Rec.	2.00–3.50
32	2,4-dimethyl-3-pentanone	565-80-0	0.67	2.41	2.82	0.03	1.44	−1.47	2.86	3	2	4	Rec.	n.a.
33	5-hexen-2-one	109-49-9	3.28	1.37	2.82	0.19	1.48	−0.68	2.91	5	5	0	Prob.	n.a.
34	ethylmethacrylate	97-63-2	0.52	1.78	2.81	0.02	1.43	−1.56	2.91	3	2	3	Rec.	4.00–5.00
35	2-propenoicacidethylester	140-88-5	1.79	1.51	2.80	0.13	1.48	−1.14	2.94	5	6	4	Prob.	1.18–1.71
36	3-methylbutanoicacidethylester	108-64-5	0.24	2.08	2.80	−0.05	1.36	−1.69	2.85	3	2	3	Rec.	n.a.
37	3,3-dimethyl-2-butanone	75-97-8	4.26	1.76	2.79	0.11	1.41	−0.58	2.84	5	2	4	Prob.	n.a.
38	butanal	123-72-8	7.33	1.61	2.77	0.15	1.43	−0.65	2.86	3	3	7	Prob.	1.35–1.40
39	4-methyl-3-penten-2-one	141-79-7	4.13	1.57	2.76	0.17	1.41	−0.22	2.79	3	6	3	Rec.	n.a.
40	methylpropionate	554-12-1	6.00	1.75	2.75	0.16	1.45	−0.74	2.86	3	3	5	Rec.	1.50–2.50
41	ethylacetate	141-78-6	7.51	1.71	2.74	0.17	1.42	−0.51	2.84	3	3	5	Rec.	0.79–1.37
42	vinylacetate	108-05-4	1.86	0.13	2.73	0.15	1.49	−1.48	2.96	5	7	5	Prob.	0.92–1.11
43	propanoicacidethenylester	105-38-4	0.62	0.13	2.73	0.05	1.43	−1.77	2.92	5	6	4	Prob.	n.a.
44	3-pentanone	96-22-0	4.39	1.84	2.72	0.12	1.42	−0.87	2.78	3	2	4	Rec.	n.a.
45	5-methyl-2(3h)-furanone	591-12-8	6.63	0.69	2.67	0.25	1.49	−0.61	2.85	7	5	1	Prob.	n.a.
46	1,1-diethoxyethane	105-57-7	1.71	2.91	2.65	−0.09	1.31	−2.15	2.65	3	2	7	Prob.	n.a.
47	2-pentanone	107-87-9	6.08	1.43	2.64	0.12	1.36	−0.54	2.69	3	2	4	Rec.	n.a.
48	diallylether	557-40-4	0.41	1.25	2.62	−0.05	1.34	−2.17	2.72	5	5	5	Prob.	n.a.
49	3-methyl-2-butanone	563-80-4	8.36	1.54	2.61	0.14	1.34	−0.41	2.65	3	2	5	Rec.	n.a.
50	2-methoxyfuran	25414-22-6	0.51	−2.92	2.60	0.17	1.52	−2.24	2.92	5	5	4	Prob.	n.a.
51	1-methoxy-1,3-butadiene	3036-66-6	0.33	−2.68	2.59	0.03	1.38	−2.41	2.84	5	5	5	Prob.	n.a.
52	paraldehyde	123-63-7	10.08	1.90	2.59	0.22	1.44	−0.98	2.72	3	1	3	Rec.	5.00–6.00
53	diethoxymethane	462-95-3	1.66	2.23	2.58	−0.17	1.21	−2.43	2.60	3	2	5	Rec.	n.a.
54	dimethylcarbonate	616-38-6	10.54	1.04	2.55	0.24	1.40	−0.62	2.72	3	1	4	Rec.	0.58–0.76
55	benzene	71-43-2	0.15	−4.62	2.54	0.04	1.51	−2.85	2.79	3	10	6	Haz.	0.70–0.95
56	cyclopentyl-methyl-ether	5614-37-9	0.63	2.75	2.53	−0.35	1.08	−2.71	2.44	5	2	7	Prob.	8.00–15.00
57	2,5-dimethylfuran	625-86-5	0.16	−3.18	2.51	−0.14	1.26	−2.65	2.69	5	5	5	Prob.	5.00–10.00
58	3-methoxy-propyne	627-41-8	6.00	0.28	2.50	0.22	1.44	−0.77	2.71	5	6	5	Prob.	n.a.
59	cycloheptatriene	544-25-2	0.10	−4.65	2.49	−0.08	1.35	−3.02	2.70	5	5	3	Prob.	n.a.
60	methyl-tert-amylether	994-05-8	0.53	2.89	2.47	−0.36	1.07	−2.92	2.37	3	2	6	Rec.	0.66–1.00
61	butylvinylether	111-34-2	0.06	−1.98	2.47	−0.30	1.09	−2.85	2.56	5	2	5	Prob.	n.a.
62	1,6-heptadiyne	2396-63-6	0.14	−5.26	2.46	0.16	1.58	−3.03	2.84	5	5	4	Prob.	n.a.
63	2-ethylfuran	3208-16-0	0.10	−4.76	2.46	−0.15	1.25	−3.05	2.68	5	5	5	Prob.	n.a.
64	thioaceticacid	507-09-5	8.76	−0.48	2.46	0.33	1.51	−0.89	2.76	3	7	4	Prob.	n.a.
65	2,4-hexadien-1-ol	111-28-4	1.95	0.27	2.45	0.21	1.35	−0.39	2.60	7	5	1	Prob.	n.a.

Table S3: *In silico* process results of selected solvents (Part 2 of 3).

Index	Solvent	CAS No.	Solvent loss [wt%]	$\log D^{\infty}_{i,\mathrm{OS/aq}}$ @ 40 °C AcAcid	Gua	Acetol	Furf	D-fruc	2,6-DMP	CHEM21 E	H	S	Cond.	Bulk price[a] [US$/kg]
66	isobutylvinylether	109-53-5	0.06	−2.50	2.45	−0.31	1.07	−2.92	2.54	5	3	5	Prob.	n.a.
67	toluene	108-88-3	0.05	−5.57	2.44	−0.13	1.31	−3.22	2.64	3	6	5	Prob.	0.85–0.99
68	2-ethoxy-2-methyl-propane	637-92-3	0.60	2.75	2.44	−0.32	1.11	−3.09	2.38	3	3	7	Prob.	0.92–1.25
69	1-ethoxybutane	628-81-9	0.14	2.87	2.43	−0.42	1.01	−3.10	2.31	3	2	5	Rec.	n.a.
70	butadione	431-03-8	13.91	0.38	2.42	0.24	1.36	−0.79	2.61	5	6	3	Prob.	n.a.
71	2-hexyne	764-35-2	0.05	−4.06	2.42	−0.24	1.15	−3.08	2.55	5	6	5	Prob.	n.a.
72	3-hexyne	928-49-4	0.05	−3.99	2.41	−0.27	1.13	−3.14	2.53	5	6	5	Prob.	n.a.
73	hexyne	693-02-7	0.06	−6.63	2.40	−0.30	1.24	−3.71	2.64	5	6	7	Prob.	n.a.
74	1,4-cyclohexadiene	628-41-1	0.10	−5.22	2.39	−0.27	1.15	−3.31	2.54	5	5	5	Prob.	n.a.
75	ethylbenzene	100-41-4	0.02	−6.00	2.39	−0.20	1.23	−3.37	2.56	5	2	4	Prob.	0.78–1.17
76	1-pentanol	71-41-0	1.96	0.41	2.38	−0.03	1.16	−0.54	2.44	3	4	3	Rec.	2.00–4.00
77	2-hexanol	626-93-7	1.12	0.37	2.38	−0.06	1.15	−0.63	2.41	5	5	3	Prob.	n.a.
78	1,4-dimethylbenzene	106-42-3	0.02	−6.04	2.37	−0.23	1.19	−3.43	2.53	5	2	4	Prob.	0.85–0.89
79	4-methyl-1-pentyne	7154-75-8	0.07	−7.02	2.37	−0.29	1.24	−3.86	2.62	5	6	5	Prob.	n.a.
80	2,5-norbornadiene	121-46-0	0.12	−4.88	2.37	−0.30	1.11	−3.24	2.53	3	7	5	Prob.	n.a.
81	1,3-cycloheptadiene	4054-38-0	0.05	−5.47	2.36	−0.33	1.08	−3.39	2.51	5	5	4	Prob.	n.a.
82	5-methyl-1-hexyne	2203-80-7	0.03	−7.11	2.36	−0.34	1.20	−3.83	2.59	5	5	5	Prob.	n.a.
83	1-heptyne	628-71-7	0.02	−7.22	2.36	−0.38	1.17	−3.88	2.57	3	2	5	Rec.	n.a.
84	isopentanol	123-51-3	2.89	0.36	2.34	0.00	1.17	−0.51	2.41	3	2	3	Rec.	1.50–3.00
85	4-methyl-2-pentanol	108-11-2	1.67	0.24	2.34	−0.02	1.17	−0.61	2.39	3	2	3	Rec.	1.50–2.50
86	di-n-propylether	111-43-3	0.09	2.35	2.33	−0.50	0.95	−3.42	2.23	5	5	5	Prob.	n.a.
87	2-methyl-1-butanol	137-32-6	2.75	0.19	2.33	0.00	1.17	−0.54	2.42	3	4	3	Rec.	n.a.
88	octyne	629-05-0	0.01	−7.68	2.33	−0.44	1.11	−4.04	2.54	3	2	4	Rec.	n.a.
89	4-penten-1-ol	821-09-0	4.60	0.38	2.33	0.18	1.25	−0.24	2.43	5	5	3	Prob.	n.a.
90	2,2’-oxybis-butane	—	0.04	2.75	2.31	−0.47	0.96	−3.44	2.19	5	5	3	Prob.	n.a.
91	3-butyn-2-one	1423-60-5	9.20	0.01	2.30	0.23	1.39	−0.80	2.52	5	5	5	Prob.	n.a.
92	cis-2-penten-1-ol	1576-95-0	4.72	0.24	2.29	0.11	1.20	−0.28	2.39	5	5	3	Prob.	n.a.
93	2,4-dimethyl-1,3-pentadiene	1000-86-8	0.02	−6.09	2.28	−0.42	0.98	−3.48	2.40	5	5	4	Prob.	n.a.
94	3-pentanol	584-02-1	3.10	0.18	2.25	0.05	1.18	−0.47	2.32	5	5	3	Prob.	n.a.
95	3-hexyn-2-ol	109-50-2	3.90	0.20	2.25	0.26	1.32	−0.36	2.43	5	6	3	Prob.	n.a.
96	2-methyl-2-pentanol	565-67-3	2.39	0.11	2.25	0.01	1.13	−0.39	2.29	5	5	3	Prob.	n.a.
97	2-methyltetrahydrofuran	96-47-9	9.03	0.96	2.21	−0.01	1.02	0.11	2.20	3	5	6	Prob.	3.00–8.00
98	2,3-dimethyl-2-butanol	594-60-5	3.47	0.05	2.21	0.05	1.15	−0.33	2.27	5	5	3	Prob.	n.a.
99	2-pentanol	6032-29-7	3.54	0.20	2.20	0.00	1.10	−0.37	2.25	5	5	3	Prob.	n.a.
100	1-penten-3-ol	616-25-1	4.47	−0.06	2.19	0.15	1.22	−0.42	2.32	5	5	3	Prob.	n.a.
101	3-methyl-2-butanol	598-75-4	4.82	0.11	2.15	0.02	1.10	−0.33	2.21	5	5	3	Prob.	n.a.
102	1-butanol	71-36-3	6.47	0.30	2.12	0.02	1.06	−0.23	2.18	3	4	3	Rec.	0.74–1.12
103	cyclohexene	110-83-8	0.05	−6.88	2.11	−0.67	0.71	−4.05	2.16	7	7	5	Haz.	1.50–2.50
104	isobutanol	78-83-1	7.40	0.19	2.09	0.04	1.07	−0.29	2.17	3	4	3	Rec.	1.15–1.65
105	2-methyl-1-hexene	6094-02-6	0.01	−7.08	2.09	−0.68	0.69	−3.94	2.13	5	5	5	Prob.	n.a.
106	cycloheptene	628-92-2	0.02	−6.74	2.09	−0.72	0.66	−4.02	2.11	5	5	5	Prob.	n.a.
107	4-methylcyclohexene	591-47-9	0.02	−7.23	2.08	−0.70	0.67	−4.05	2.11	5	5	5	Prob.	n.a.
108	trimethylsilanol	1066-40-6	5.04	−0.09	2.07	0.04	1.09	−0.42	2.15	3	2	4	Rec.	n.a.
109	4,4-dimethyl-1-pentene	762-62-9	0.01	−8.09	2.07	−0.67	0.69	−4.12	2.11	5	6	5	Prob.	n.a.
110	2-methyl-1-heptene	15870-10-7	0.00	−7.45	2.06	−0.71	0.65	−4.01	2.09	5	5	4	Prob.	n.a.
111	2,3-dimethyl-2-butene	563-79-1	0.02	−8.29	2.06	−0.67	0.70	−4.14	2.11	7	3	5	Prob.	n.a.
112	(e)-3-methyl-2-pentene	616-12-6	0.02	−7.83	2.05	−0.70	0.67	−4.10	2.08	5	6	5	Prob.	n.a.
113	2,4,4-trimethyl-1-pentene	107-39-1	0.01	−7.96	2.05	−0.71	0.64	−4.11	2.08	5	5	5	Prob.	n.a.
114	4-methyl-1-hexene	3769-23-1	0.01	−8.31	2.04	−0.71	0.64	−4.19	2.08	5	5	5	Prob.	n.a.
115	1-heptene	592-76-7	0.00	−8.01	2.04	−0.70	0.66	−4.17	2.07	3	2	5	Rec.	n.a.
116	1,4-pentadien-3-ol	922-65-6	7.21	−0.22	2.04	0.34	1.28	−0.26	2.25	5	5	3	Prob.	n.a.
117	1-methylcyclohexene	591-49-1	0.01	−7.82	2.03	−0.75	0.62	−4.23	2.05	5	5	5	Prob.	n.a.
118	4-penten-2-ol	625-31-0	8.55	0.17	2.03	0.11	1.07	−0.13	2.10	5	5	3	Prob.	n.a.
119	1-methylcyclopentene	693-89-0	0.03	−7.68	2.03	−0.73	0.64	−4.13	2.06	5	6	5	Prob.	n.a.
120	octene	111-66-0	0.00	−8.41	2.02	−0.74	0.61	−4.27	2.04	7	2	4	Prob.	1.00–1.50
121	2,2,4-trimethyl-2-pentene	107-40-4	0.00	−8.34	2.01	−0.72	0.62	−4.26	2.04	5	5	4	Prob.	n.a.
122	trans-2-heptene	14686-13-6	0.00	−8.47	2.00	−0.74	0.61	−4.31	2.01	5	5	5	Prob.	n.a.
123	trans-2-octene	13389-42-9	0.00	−8.65	1.99	−0.76	0.58	−4.35	1.99	5	5	4	Prob.	n.a.
124	3-methyl-1-pentyn-3-ol	77-75-8	6.90	−0.41	1.97	0.39	1.32	−0.33	2.22	5	4	3	Prob.	n.a.
125	trans-3-octene	14919-01-8	0.00	−8.85	1.96	−0.78	0.55	−4.42	1.95	7	6	4	Prob.	n.a.

Table S4: *In silico* process results of selected solvents (Part 3 of 3).

Index	Solvent	CAS No.	Solvent loss	$\log D^{\infty}_{i,\mathrm{OS/aq}}$ @ 40 °C						CHEM21				Bulk price[a]
			[wt%]	AcAcid	Gua	Acetol	Furf	D-fruc	2,6-DMP	E	H	S	Cond.	[US$/kg]
126	(e)-4-octene	14850-23-8	0.00	−8.89	1.96	−0.78	0.55	−4.42	1.95	5	5	4	Prob.	n.a.
127	2-methyl-2-butanol	75-85-4	8.14	−0.08	1.84	0.03	0.94	−0.14	1.89	3	4	4	Prob.	2.00–4.00
128	2-butenol	6117-91-5	12.31	0.18	1.83	0.11	0.97	−0.05	1.91	5	5	3	Prob.	3.00–5.00
129	2,2,3-trimethyl-butane	464-06-2	0.01	−11.32	1.79	−0.99	0.32	−4.97	1.75	7	3	5	Prob.	n.a.
130	1,1-dimethylcyclohexane	590-66-9	0.00	−11.29	1.77	−1.02	0.29	−5.05	1.73	5	5	4	Prob.	n.a.
131	2,2,4-trimethylhexane	16747-26-5	0.00	−11.48	1.77	−0.99	0.31	−5.02	1.72	5	5	4	Prob.	n.a.
132	2,2,4-trimethylpentane	540-84-1	0.00	−11.39	1.77	−0.99	0.31	−5.00	1.73	7	2	5	Prob.	1.50–2.50
133	2,3,4-trimethylpentane	565-75-3	0.00	−11.51	1.77	−1.00	0.29	−5.02	1.73	5	5	4	Prob.	n.a.
134	2,2-dimethylhexane	590-73-8	0.00	−11.46	1.77	−1.00	0.29	−5.01	1.72	5	5	5	Prob.	n.a.
135	3,3-dimethylpentane	562-49-2	0.00	−11.44	1.77	−1.01	0.29	−5.01	1.72	5	5	5	Prob.	n.a.
136	2,2-dimethylpentane	590-35-2	0.00	−11.41	1.76	−1.01	0.29	−5.00	1.72	5	6	5	Prob.	n.a.
137	3,4-dimethylhexane	583-48-2	0.00	−11.60	1.76	−1.02	0.27	−5.06	1.71	5	5	4	Prob.	n.a.
138	2,4-dimethylhexane	589-43-5	0.00	−11.56	1.75	−1.02	0.28	−5.05	1.70	5	5	4	Prob.	n.a.
139	2,3-dimethylpentane	565-59-3	0.00	−11.57	1.75	−1.03	0.27	−5.05	1.70	7	2	5	Prob.	n.a.
140	cis-1,3-dimethylcyclohexane	591-21-9	0.00	−11.40	1.75	−1.04	0.27	−5.09	1.70	5	5	4	Prob.	n.a.
141	2,4-dimethylpentane	108-08-7	0.00	−11.48	1.75	−1.02	0.28	−5.03	1.70	5	6	5	Prob.	n.a.
142	2,5-dimethylhexane	592-13-2	0.00	−11.56	1.75	−1.02	0.27	−5.05	1.69	5	5	3	Prob.	n.a.
143	2-methyl-3-butene-2-ol	115-18-4	10.76	−0.16	1.74	0.15	1.00	−0.19	1.85	3	2	4	Rec.	n.a.
144	3-methylheptane	589-81-1	0.00	−11.68	1.74	−1.04	0.25	−5.09	1.68	5	5	4	Prob.	n.a.
145	2-methylheptane	592-27-8	0.00	−11.65	1.74	−1.04	0.25	−5.08	1.67	5	5	4	Prob.	n.a.
146	ethylcyclohexane	1678-91-7	0.00	−11.58	1.73	−1.07	0.24	−5.14	1.68	7	2	3	Prob.	n.a.
147	3-methyl-hexane	589-34-4	0.00	−11.65	1.73	−1.05	0.25	−5.08	1.67	5	5	5	Prob.	n.a.
148	2-methylhexane	591-76-4	0.00	−11.62	1.73	−1.05	0.25	−5.08	1.67	5	5	5	Prob.	n.a.
149	methylcyclohexane	108-87-2	0.01	−11.50	1.73	−1.07	0.24	−5.13	1.67	7	2	6	Prob.	1.70–1.80
150	octane	111-65-9	0.00	−11.69	1.73	−1.05	0.24	−5.09	1.66	7	2	4	Prob.	1.50–2.00
151	cycloheptane	291-64-5	0.01	−11.59	1.73	−1.10	0.21	−5.16	1.67	5	5	4	Prob.	n.a.
152	n-heptane	142-82-5	0.00	−11.67	1.72	−1.06	0.23	−5.10	1.65	7	2	6	Prob.	2.16–2.23
153	cyclohexane	110-82-7	0.01	−11.64	1.70	−1.11	0.20	−5.18	1.65	7	3	6	Prob.	0.91–0.95
154	2-butanol	78-92-2	12.41	0.06	1.64	0.03	0.84	−0.10	1.69	3	2	3	Rec.	1.00–1.50
155	2-methyl-3-butyn-2-ol	115-19-5	70.38	−0.00	0.00	0.00	0.00	−0.00	0.00	3	6	4	Prob.	n.a.
156	tert-butanol	75-65-0	81.34	2.29	−1.39	−3.15	−2.07	−5.86	−1.66	3	3	4	Rec.	0.74–1.71

The column headers in Tables S2–S4 use abbreviated identifiers for the SynBO target solutes and for the CHEM21 safety, health, and environment classification. The solute labels AcAcid, Gua, Acetol, Furf, D-fruc, and 2,6-DMP correspond to acetic acid, guaiacol, acetol, furfural, D-fructose, and 2,6-dimethoxyphenol, respectively. The CHEM21 sub-scores E, H, and S denote the Environment, Health, and Safety axes proposed by Prat et al.[1], and the consolidated condition (Cond.) reports the overall verdict assigned to each solvent within the same framework, abbreviated as Rec. for Recommended, Prob. for Problematic, and Haz. for Hazardous.

The last column of each table reports the bulk price[a] expressed in US$/kg, corresponding to industrial-scale supply (>1 t) and aggregated as a low–high range over the period Q3 2025–Q1 2026. Entries flagged as "n.a." indicate that no industrial-scale bulk-supply data was available at the time of compilation; the corresponding solvents are nevertheless retained in the table because their thermodynamic and CHEM21 descriptors remain informative for the screening cas-

cade. The pricing data were assembled from a combination of recognized commercial pricing intelligence providers (ChemAnalyst, Intratec, IMARC Group, businessanalytiq, ECHEMI, ICIS, and PriceWatch.ai) and supplier quotations from Alibaba and ChemicalBook, with the full per-solvent classification (Verified, Estimated, or n.a.) and the underlying assignment rules documented in Section .

Bulk price estimation for industrial-scale implementation

The bulk prices reported in Tables S2–S4 correspond to industrial-scale supply (>1 t) expressed in US$/kg, suitable for tank-truck or ISO-tank deliveries. Such prices are not distributed through laboratory catalogues but through commercial pricing intelligence services and producer quotations. For 43 of the 156 candidates a public bulk price could be retrieved or estimated; the remaining 113 entries are reported as “n.a.” because no industrial-scale bulk-supply data was available at the time of compilation. Bulk prices were compiled through the structured three-tier procedure described below.

Data hierarchy

For each solvent in the final list, an attempt was made to classify the bulk price into one of three tiers according to the reliability of the underlying data source:

- **Verified**: a public quotation expressed in US$/metric ton (or equivalent) is available from a recognized commercial pricing intelligence provider (e.g., ChemAnalyst, Intratec, IMARC Group, businessanalytiq, ECHEMI, ICIS, or PriceWatch.ai) for the period Q3 2025–Q1 2026, with explicit regional coverage (Asia-Pacific, Europe, and/or North America). Twenty-three candidates met this criterion, the majority corresponding to high-volume commodity chemicals such as ethyl acetate (CAS 141-78-6), toluene (CAS 108-88-3), benzene (CAS 71-43-2), *n*-butanol (CAS 71-36-3), cyclohexane (CAS 110-82-7), and dimethyl carbonate (CAS 616-38-6).

- **Estimated**: no direct quotation is available, but the bulk price can be bracketed using a combination of analogies with verified compounds, derivative relationships, reported market-size information, and supplier price signals. Twenty candidates fall in this category. The estimation procedure is detailed below.

- **Not found**: no commercial pricing information could be retrieved through any public source,

and the compound cannot be anchored confidently to a verified analogue. This tier groups 113 candidates, most of which are low-volume, specialty, or laboratory-only chemicals (e.g., saturated and unsaturated aldehydes, short-chain ethers, alkyl furans, and niche alkanes) that are not traded as bulk commodities.

Estimation techniques

When a verified quotation could not be retrieved, the bulk price range was constructed using one or more of the following four techniques, applied in order of preference:

1. *Chemical-class analogy.* The price of a compound was anchored to the verified range of a reference compound belonging to the same functional-group family and having a similar molecular weight. This approach exploits the fact that, within a homologous series, feedstock cost and process complexity are broadly comparable, so that the bulk price is expected to lie within a narrow multiplicative factor of the analogue. For example, isobutyl acetate (CAS 110-19-0) and sec-butyl acetate (CAS 105-46-4) were bracketed using the verified range of *n*-butyl acetate (CAS 123-86-4), to which a small premium was added to reflect the higher cost of the branched alcohol feedstock relative to *n*-butanol.

2. *Derivative relationship.* The price of a compound obtained through one additional synthetic step from a verified precursor was expressed as the precursor price augmented by a processing margin consistent with typical downstream conversion costs. This technique was used, for instance, for 4-methyl-2-pentanol (CAS 108-11-2), which is produced by catalytic hydrogenation of 4-methyl-2-pentanone (MIBK, CAS 108-10-1), and for diethyl carbonate (CAS 105-58-8), which is obtained from dimethyl carbonate (verified) by transesterification with ethanol.

3. *Anchoring to a neighbouring commodity.* For near-commodity compounds whose market is dominated by a single application sector (primarily fuel oxygenates and solvent-grade alkanes), the estimate was based on the publicly quoted price of the closest traded molecule in

the same application space. Methyl tert-amyl ether (TAME, CAS 994-05-8) was anchored to MTBE; *n*-octane (CAS 111-65-9) and 2,2,4-trimethylpentane (CAS 540-84-1) were anchored to the verified range of *n*-hexane.

4. *Qualitative market reports.* For specialty solvents whose global market size is small ($\sim$ US\$ 10^8–10^9) and for which neither analogies nor derivative relationships apply, the bulk price range was taken from qualitative statements in publicly available market-research summaries (Procurement Resource, ECHEMI market news, Fortune Business Insights, Valuates Reports) or from bulk-quantity quotations published on B2B platforms (Made-in-China, ChemicalBook, LookChem, Alibaba). This was the case for cyclopentyl methyl ether (CPME, CAS 5614-37-9), 2-methyltetrahydrofuran (CAS 96-47-9), and 2,5-dimethylfuran (CAS 625-86-5), all of which are emerging bio-based solvents with limited industrial production.

Uncertainty and applicability

Verified entries carry a relative uncertainty of approximately ±10–20%, reflecting the inherent regional dispersion of industrial prices between Asia-Pacific, Europe, and North America across Q3 2025–Q1 2026. Estimated entries carry a substantially larger uncertainty, of the order of ±30–50%, because they are constructed from analogies rather than from observed transactions; they should therefore be interpreted as order-of-magnitude references rather than as market quotations. Compounds classified as *Not found* were not assigned any price and were excluded from economic comparisons at the industrial scale; this is a conservative choice that avoids propagating speculative figures into downstream process-design decisions. Table S5 summarizes the compound-specific justifications used for each *Estimated* entry, including the reference compound or analogue employed and the estimation technique applied.

The information summarized in Table S5, together with the verified quotations reported in the supplementary spreadsheet, provides the economic layer used in the industrial-scale comparison of the four selected extractants (DMC, CPME, 2-MeTHF, and 2-butanol). All bulk prices were retrieved between October 2025 and March 2026; accordingly, they should be revisited if the can-

Table S5: Compound-specific justifications for the *Estimated* bulk prices assigned to candidates without direct public quotations.

#	Solvent	CAS No.	Bulk price [US$/kg]	Technique	Reference / justification
1	Isobutyl acetate	110-19-0	1.20–1.80	Class analogy	Anchored to verified *n*-butyl acetate (0.96–1.76) with a premium for isobutanol feedstock.
2	tert-Butyl acetate	540-88-5	2.50–4.00	Market report	Branched ester obtained via isobutylene + acetic acid; bulk quotations on Made-in-China for volumes $\geq$ 1 MT.
3	Ethyl propionate	105-37-3	1.50–2.50	Class analogy	C_3-ester class; premium over *n*-propyl acetate (verified 1.32–1.51) for propionic acid feedstock.
4	Methyl propionate	554-12-1	1.50–2.50	Class analogy	C_3-ester class; aligned with verified ethyl acetate and *n*-propyl acetate ranges.
5	Diethyl carbonate	105-58-8	0.90–1.50	Derivative	Obtained from DMC (verified 0.58–0.76) via transesterification with ethanol; chemical grade only (battery grade excluded).
6	Isobutyl methacrylate	106-63-8	2.00–3.50	Class analogy	Methacrylate ester anchored to verified ethyl acrylate (1.18–1.71) with a premium for the methacrylate route.
7	Cyclopentyl methyl ether (CPME)	5614-37-9	8.00–15.00	Market report	Specialty solvent produced mainly by Zeon Corp.; global market $\sim$US$ 268 M (2024); price derived from bulk-quantity supplier quotations.
8	2,5-Dimethylfuran	625-86-5	5.00–10.00	Market report	Emerging bio-based solvent; production cost reported as 2–3× gasoline cost.
9	Methyl tert-amyl ether (TAME)	994-05-8	0.66–1.00	Commodity anchor	Fuel oxygenate anchored to MTBE (661 US$/MT, SE Asia, August 2025, Intratec).
10	1-Pentanol	71-41-0	2.00–4.00	Class analogy	Specialty C_5 alcohol; premium 2–3× over verified *n*-butanol due to lower production volume.
11	Isopentanol	123-51-3	1.50–3.00	Class analogy	Obtained as fusel-oil derivative or via oxo synthesis; priced slightly below 1-pentanol.
12	4-Methyl-2-pentanol (MIBC)	108-11-2	1.50–2.50	Derivative	Obtained by hydrogenation of verified MIBK (1.16–1.98) with a small processing margin.
13	2-Methyltetrahydrofuran (2-MeTHF)	96-47-9	3.00–8.00	Market report	Bulk price estimated as $\sim$1/3 of catalogue prices (10–50 US$/kg for laboratory packs).
14	Cyclohexene	110-83-8	1.50–2.50	Derivative	Partial dehydrogenation of verified cyclohexane (0.91–0.95); premium $\sim$2× for lower production volume.
15	1-Octene	111-66-0	1.00–1.50	Commodity anchor	Linear α-olefin C_8; range consistent with Chevron Phillips and Shell LAO reports.
16	2-Methyl-2-butanol (tert-amyl)	75-85-4	2.00–4.00	Class analogy	Tertiary C_5 alcohol; premium over verified tert-butanol (0.74–1.71) for lower production volume.
17	Crotyl alcohol (2-butenol)	6117-91-5	3.00–5.00	Market report	Specialty unsaturated C_4 alcohol; range from bulk quotations on ChemicalBook and LookChem.
18	2,2,4-Trimethylpentane	540-84-1	1.50–2.50	Commodity anchor	Reference fuel grade; premium over gasoline components for high-purity isomer.
19	*n*-Octane	111-65-9	1.50–2.00	Commodity anchor	Solvent-grade alkane; anchored to verified *n*-hexane ($\sim$1079 US$/MT, ChemAnalyst Q4 2025).
20	2-Butanol	78-92-2	1.00–1.50	Class analogy	Linear interpolation between verified *n*-butanol (0.74–1.12) and tert-butanol (0.74–1.71).

didate list is reused for future techno-economic assessments, since the global chemicals market has exhibited significant volatility over the past two years in response to feedstock cost fluctuations and regional supply disruptions.

Hansen solubility parameter analysis

Target HSP values and R_a ranking approach

Hansen solubility parameters for the two target solutes were estimated via the Hoftyzer–van Krevelen group-contribution method[2] and cross-checked against the tabulation of Hansen[3]:

- Guaiacol (2-methoxyphenol, CAS 90-05-1): $\delta_D = 19.4$, $\delta_P = 7.8$, $\delta_H = 12.3$ MPa$^{1/2}$
- 2,6-Dimethoxyphenol (syringol, CAS 91-10-1): $\delta_D = 19.6$, $\delta_P = 7.0$, $\delta_H = 11.7$ MPa$^{1/2}$

Solvents are ranked by ascending Hansen distance R_a (Eq. 1), where lower R_a indicates greater predicted affinity. No interaction radius R_0 or relative energy difference (RED = R_a/R_0) was used, because the solubility sphere framework was designed for polymer dissolution and no experimentally determined R_0 exists for small-molecule phenolic solutes; Hansen[3] (Table A.1) tabulates HSP components (δ_D, δ_P, δ_H) but not R_0 for phenol or any methoxyphenol. Using R_a as a direct ranking metric, without imposing a binary good/bad threshold, avoids introducing an untraceable parameter. This approach is consistent with the 3D HSP visualization model of Jiang et al.[4] for phenolic extraction from bio-oil, which ranks solvents by proximity in HSP space without requiring an interaction radius.

$$R_a^2 = 4(\delta_D^s - \delta_D^t)^2 + (\delta_P^s - \delta_P^t)^2 + (\delta_H^s - \delta_H^t)^2 \quad (1)$$

HSP results

Table S6: Hansen solubility parameter distances (R_a, MPa$^{1/2}$) for the four tested bio-based solvents and five conventional reference solvents. Lower R_a indicates greater predicted affinity (see Eq. 1). $R_{a,\text{avg}} = (R_{a,\text{G}} + R_{a,\text{DMP}})/2$.

**Solvent**	δ_D	δ_P	δ_H	$R_{a,\text{G}}$	$R_{a,\text{DMP}}$	$R_{a,\text{avg}}$
DMC	15.5	3.9	9.7	9.15	8.98	9.07
CPME	16.7	4.3	4.3	10.29	9.74	10.02
2-MeTHF	16.9	5.0	4.3	9.81	9.35	9.58
2-BuOH	15.8	5.7	14.5	7.83	8.15	7.99
DCM	18.2	6.3	6.1	6.77	6.21	6.49
$CHCl_3$	17.8	3.1	5.7	8.65	8.05	8.35
n-Hexane	14.9	0.0	0.0	17.15	16.55	16.85
Toluene	18.0	1.4	2.0	12.45	11.65	12.05
Diethyl ether	14.5	2.9	5.1	13.11	12.82	12.97

All four tested bio-based solvents exhibit R_a distances below 10.3 MPa$^{1/2}$ for both target solutes, indicating strong predicted HSP affinity. Among them, 2-BuOH shows the shortest average distance ($R_{a,\text{avg}} = 7.99$), followed by DMC (9.07), 2-MeTHF (9.58), and CPME (10.02). Dichloromethane shows the overall shortest distance ($R_{a,\text{avg}} = 6.49$), consistent with its historical use for phenolic extraction despite its Hazardous CHEM21 verdict. Toluene ($R_{a,\text{avg}} = 12.05$), diethyl ether (12.97), and *n*-hexane (16.85) show substantially larger distances, consistent with their weaker extraction capacity for methoxyphenols as predicted by COSMO-RS.

Agreement between HSP and COSMO-RS screening

The correlation between the HSP affinity ($-R_{a,\text{avg}}$) and the TOPSIS score across the 40-solvent pool is weak: Spearman $\rho = 0.36$ ($p = 0.03$). This weak correlation indicates that HSP and COSMO-RS probe overlapping but distinct aspects of solvent–solute affinity: HSP captures global $\delta_D/\delta_P/\delta_H$ matching within the Hildebrand framework, while COSMO-RS resolves the full σ-profile including directional H-bonding and charge-density anisotropy. Systematic divergence between HSP and COSMO-RS for aromatic H-bonding systems has been documented by Niederquell et al.[5] for pharmaceutical solubility prediction and by Mathieu[6] for estimation of Hansen parameters; Klamt[7]

provides the broader argument that COSMO-RS subsumes the information content of HSP when σ-moments are available.

Rationale for not using HSP as a pre-filter or MCDA criterion

Three considerations argue against deploying an R_a-based threshold as a gate before COSMO-RS or as a criterion in the TOPSIS aggregation:

1. The weak Spearman correlation ($\rho = 0.36$) between $-R_{a,\text{avg}}$ and TOPSIS score indicates that R_a carries limited independent information beyond what COSMO-RS already captures. Introducing R_a as a fifth MCDA criterion would add a weakly informative, redundant axis.

2. The HSP framework was designed for polymer dissolution; applying it to liquid–liquid extraction of small molecules introduces conceptual limitations (no interaction radius R_0 is available for the target solutes, and the three-parameter decomposition does not resolve directional H-bonding)[3]. Any arbitrary R_a cutoff would be methodologically unjustified without experimental solubility data in 20–30 solvents to fit a sphere.

3. Among the top-ten TOPSIS candidates, three esters (isobutyl, *tert*-butyl, and *sec*-butyl acetate) show $R_{a,\text{avg}}$ values above 10 $\text{MPa}^{1/2}$ despite strong COSMO-RS performance (TOPSIS > 0.78). An arbitrary R_a cutoff would risk excluding validated candidates.

HSP is therefore retained as a complementary transparency analysis: the four tested solvents show R_a distances comparable to or shorter than the conventional benchmarks that pass the CHEM21 filter ($CHCl_3$, DCM), strengthening the physical plausibility of the experimental design. This reading of HSP as complement rather than gate is consistent with the approaches of Santaella et al.[8], Soukup-Carné et al.[9], and Wu et al.[10].

Multi-criteria ranking of the 40-solvent pool and robustness analysis

TOPSIS-ranked 40-solvent pool

Table S7 reports the full numerical content of the multi-criteria ranking: the 40 solvents that survive the hard-constraint cascade (C1–C5), in descending TOPSIS order, with the COSMO-RS-predicted distribution coefficients for guaiacol and 2,6-DMP, the CHEM21 S/H/E scores and categorical verdict, and the normal boiling point T_b. The four candidates retained for experimental validation (DMC, CPME, 2-MeTHF, 2-BuOH) are highlighted in bold. The table is also the input dataset of the robustness tests reported in Tables S8–S10 below.

Table S7: TOPSIS-ranked pool of 40 solvents surviving the hard-constraint cascade (C1–C5). All $\log D_i^{\mathrm{x},\infty}$ values are COSMO-RS predictions at 313.15 K with the BP-TZVPD-FINE parametrization. CHEM21 S/H/E scores and verdicts follow Prat et al.[1] The TOPSIS column uses $\mathbf{w}$ = [0.30, 0.30, 0.22, 0.18] over $[c_{\log D_{\mathrm{G}}^{\mathrm{x},\infty}}, c_{\log D_{\mathrm{DMP}}^{\mathrm{x},\infty}}, c_{\mathrm{CHEM21}}, c_{\mathrm{BP}}]$. The four solvents retained for experimental validation are typeset in bold.

Rank	Solvent	$\log D_{\mathrm{G}}^{\mathrm{x},\infty}$	$\log D_{\mathrm{DMP}}^{\mathrm{x},\infty}$	S	H	E	max	Verdict	T_{b} / °C	TOPSIS
1	ethyl methacrylate	2.81	2.91	3	2	3	3	Recommended	127.5	0.940
2	*n*-propyl acetate	2.94	3.02	4	2	3	4	Recommended	117.6	0.884
3	isobutyl acetate	2.92	2.99	4	2	3	4	Recommended	135.5	0.883
4	*tert*-butyl acetate	2.91	2.96	4	2	3	4	Recommended	127.4	0.880
5	isopropyl acetate	2.88	2.97	4	2	3	4	Recommended	115.2	0.879
6	ethyl propionate	2.88	2.97	4	2	3	4	Recommended	112.7	0.879
7	4-methyl-2-pentanone (MIBK)	2.84	2.88	4	2	3	4	Recommended	119.0	0.867
8	**dimethyl carbonate (DMC)**	**2.55**	**2.72**	**4**	**1**	**3**	**4**	**Recommended**	**90.5**	**0.795**
9	*sec*-butyl acetate	2.93	2.98	3	5	5	5	Problematic	135.2	0.782
10	methyl propionate	2.75	2.86	5	3	3	5	Recommended	88.1	0.762
11	ethyl acetate	2.74	2.84	5	3	3	5	Recommended	93.2	0.759
12	diethyl carbonate	2.88	3.01	3	2	3	3	Recommended	148.0	0.753
13	isopentanol	2.34	2.41	3	2	3	3	Recommended	124.2	0.735
14	4-methyl-2-pentanol	2.34	2.39	3	2	3	3	Recommended	136.4	0.731
15	*n*-butyl acetate	2.94	3.00	4	2	3	4	Recommended	140.7	0.724
16	1-pentanol	2.38	2.44	3	4	3	4	Recommended	128.1	0.716
17	2,5-dimethylfuran	2.51	2.69	5	5	5	5	Problematic	111.8	0.713
18	ethyl acrylate	2.80	2.94	4	6	5	6	Problematic	118.4	0.690
19	ethylbenzene	2.39	2.56	4	2	5	5	Problematic	134.4	0.677
20	1,4-dimethylbenzene	2.37	2.53	4	2	5	5	Problematic	135.3	0.669
21	isobutyl acrylate	2.83	2.92	3	2	5	5	Recommended	156.6	0.654
22	toluene	2.44	2.64	5	6	3	6	Problematic	110.6	0.626
23	1-butanol	2.12	2.18	3	4	3	4	Recommended	109.4	0.624
24	vinyl acetate	2.73	2.96	5	7	5	7	Problematic	87.1	0.620
25	isobutanol	2.09	2.17	3	4	3	4	Recommended	104.0	0.620
26	methyl *tert*-amyl ether	2.47	2.37	6	2	3	6	Recommended	85.5	0.598
27	**cyclopentyl methyl ether (CPME)**	**2.53**	**2.44**	**7**	**2**	**5**	**7**	**Problematic**	**108.7**	**0.558**
28	paraldehyde	2.59	2.72	3	1	3	3	Recommended	214.0	0.547
29	2-methyl-2-butanol	1.84	1.89	4	4	3	4	Problematic	114.6	0.546
30	**2-methyltetrahydrofuran (2-MeTHF)**	**2.21**	**2.20**	**6**	**5**	**3**	**6**	**Problematic**	**80.0**	**0.544**
31	2-ethoxy-2-methylpropane	2.44	2.38	7	3	3	7	Problematic	98.9	0.542
32	**2-butanol (2-BuOH)**	**1.64**	**1.69**	**3**	**2**	**3**	**3**	**Recommended**	**101.6**	**0.534**
33	butanal	2.77	2.86	7	3	3	7	Problematic	60.3	0.528
34	2-buten-1-ol	1.83	1.91	3	5	5	5	Problematic	114.1	0.507
35	1-octene	2.02	2.04	4	2	7	7	Problematic	109.0	0.464
36	2,2,4-trimethylpentane	1.77	1.73	5	2	7	7	Problematic	88.8	0.417
37	methylcyclohexane	1.73	1.67	6	2	7	7	Problematic	93.9	0.411
38	*n*-octane	1.73	1.66	4	2	7	7	Problematic	107.0	0.410
39	*n*-heptane	1.72	1.65	6	2	7	7	Problematic	81.2	0.409
40	cyclohexane	1.70	1.65	6	3	7	7	Problematic	64.8	0.246

Purpose and reading guide

Any MCDA ranking is built on two classes of inputs that carry residual uncertainty: the numerical scores entering each criterion (here, the COSMO-RS-predicted distribution coefficients $\log D_i^{\mathrm{x},\infty}$,

which carry a model error of the order $\sigma_{\log D} \sim 0.3$–0.5 for hydrogen-bonded systems[7]) and the weight vector that aggregates them, which reflects an analyst's judgment of relative importance and is intrinsically subjective[11,12]. A ranking that changes substantially when these inputs are perturbed within their realistic uncertainty ranges cannot be claimed to identify robust top performers. The two complementary tests reported in this section probe the ranking from each angle: the dual Monte Carlo perturbs the inputs jointly to map how each of the four experimentally tested solvents distributes across the 40-position pool under uncertainty, and the leave-one-criterion-out (LOO) sensitivity removes one criterion at a time to verify that no single criterion alone is responsible for the result. Throughout this section, the four solvents subsequently retained for experimental validation (DMC, CPME, 2-MeTHF, 2-BuOH) are tracked individually because the conclusions of the paper rest on their specific positions in the pool, not on the average behavior of the 40 candidates.

Dual Monte Carlo setup

Two independent sources of uncertainty are perturbed simultaneously. First, the TOPSIS weight vector $\mathbf{w} = [0.30, 0.30, 0.22, 0.18]$ is multiplied element-wise by $1 + U(-0.3, 0.3)$ and renormalized to sum to one. The ±30% envelope is the standard range used to test ranking stability against weight elicitation in solvent-selection MCDA[8,11,13]: it is wide enough to absorb plausible reweighting of the two methoxyphenol targets (e.g., a 0.35/0.25 instead of 0.30/0.30 split) and of the SHE versus boiling-point trade-off, but narrow enough that the criteria retain their declared roles (none collapses to zero, none dominates the aggregation). Second, the $\log D_{\mathrm{G}}^{\mathrm{x},\infty}$ and $\log D_{\mathrm{DMP}}^{\mathrm{x},\infty}$ values for every one of the 40 solvents are perturbed independently by Gaussian noise of standard deviation $\sigma_{\log D} = 0.30$, consistent with the upper-bound COSMO-RS error reported for H-bonded systems[7]. The CHEM21 score and boiling-point window class are integer/categorical and are not noised. $N = 3000$ independent draws are generated and the full TOPSIS aggregation (normalization, weighting, distance to ideal solutions) is rerun for each draw, producing a distribution of 3000 ranks for every solvent.

In Table S8, the *Median* column reports the central rank of each tracked solvent across the 3000

perturbed runs (rank 1 = best, rank 40 = worst), p_{10} and p_{90} bound the 10th and 90th percentiles of that rank distribution, the *Top-10 %* column reports the fraction of runs in which the solvent occupied one of the first ten positions, and *Top-quartile %* reports the fraction of runs in which it was in the top ten of the 40-solvent pool (i.e., the 25% best). A high Top-10 percentage with a tight p_{10}–p_{90} window indicates that the candidate is a stable top performer; a high percentage with a wide window indicates that the candidate occasionally ranks well but is volatile; a near-zero percentage indicates that the candidate does not credibly belong to the leading group.

Table S8: Monte Carlo rank distribution for the four tested solvents under $\sigma_{\log D} = 0.30$ and weight perturbations ±30%, $N = 3000$.

**Solvent**	**Median**	p_{10}	p_{90}	**Top-10 %**	**Top-quartile %**
DMC	12	5	21	40%	40%
CPME	27	20	32	0%	0%
2-MeTHF	30	24	34	0%	0%
2-BuOH	32	27	36	0%	0%

Reading Table S8: DMC reaches the top ten in 40% of the perturbed runs and shows the widest p_{10}–p_{90} window (5–21), signaling a candidate that is competitive with the leading C_3–C_5 ester cluster but whose precise position fluctuates with the weight allocation between guaiacol and 2,6-DMP. The other three tested solvents (CPME, 2-MeTHF, 2-BuOH) never enter the top ten across the 3000 runs and remain confined to the lower half of the pool, which is consistent with their nominal mid-pool ranks (27, 30, 32 of 40) and with the fact that they were retained for experimental validation on grounds other than peak TOPSIS score: CPME and 2-MeTHF as bio-based ethers with documented industrial precedent, and 2-BuOH as a thermodynamic negative-reference (low-capacity, Recommended) used to test the discriminating power of the protocol. The Monte Carlo therefore separates the four candidates into two distinct groups, a result that the experimental program reproduced.

To probe how this picture degrades as the COSMO-RS error envelope widens, the noise standard deviation $\sigma_{\log D}$ is swept across three regimes: 0.10 (an optimistic estimate representative of best-case predictions for non-associating systems), 0.30 (the default value used in Table S8), and 0.50 (the upper-bound error reported for hydrogen-bonded, polar systems with multiple H-donor

sites)[7]. The DMC rank distribution is reported in Table S9 as the most informative diagnostic, since DMC sits at the boundary between the leading ester cluster and the bulk of the pool and is therefore the most sensitive of the four tested solvents to input noise.

Table S9: Sensitivity of DMC's Monte Carlo rank to the log $D^{x,\infty}$ noise standard deviation $\sigma_{\log D}$.

$\sigma_{\log D}$	**DMC median**	**Interquartile range**
0.10	10	[9–12]
0.30	12	[8–17]
0.50	14	[8–20]

The DMC median rank shifts modestly with the noise level (10 at $\sigma = 0.10$, 12 at $\sigma = 0.30$, 14 at $\sigma = 0.50$) while the interquartile range widens monotonically from [9–12] to [8–20]. Even at the pessimistic $\sigma_{\log D} = 0.50$, DMC remains in the top half of the pool with a top-20 probability of 76%, meaning that it would be retained as a credible candidate under the worst plausible COSMO-RS error envelope for this class of systems. The Spearman rank correlation between the nominal 40-solvent ranking and the Monte Carlo median ranking is $\rho = 0.977$; values close to unity indicate that the same solvents occupy the same relative positions before and after perturbation, so the aggregation is stable and the input uncertainty does not propagate into a reshuffled hierarchy of candidates.

In summary, the dual Monte Carlo establishes that (i) the four experimentally tested solvents are correctly separated into a top-of-the-pool candidate (DMC) and three mid-pool candidates (CPME, 2-MeTHF, 2-BuOH), in agreement with their nominal ranks; (ii) the separation is preserved under joint ±30% weight perturbation and Gaussian log D noise; and (iii) widening the noise envelope to the upper-bound COSMO-RS error does not displace DMC out of the top half of the pool. What this analysis cannot reveal, by construction, is whether the result is being driven by one criterion alone or by the balanced contribution of all four. The leave-one-criterion-out analysis below addresses precisely that question.

Leave-one-criterion-out analysis

LOO probes the dependence of the ranking on each individual criterion by removing one criterion at a time, renormalizing the three remaining weights to sum to one, and rerunning the TOPSIS aggregation; the rank that each candidate occupies in the resulting three-criterion ranking is recorded and compared against its nominal four-criterion rank. The construct rests on the heuristic adopted by Tobiszewski et al.[11] and Zavadskas and Turskis[12] for green-solvent and general-MCDA screening: a candidate whose rank stays within a narrow window across all single-criterion drops is said to be *not load-bearing* on any individual criterion (its position is supported by the joint contribution of all four criteria), whereas a candidate whose rank shifts by more than five positions when one specific criterion is removed is identified as primarily driven by that criterion. The latter outcome is not a flaw, but a useful diagnostic: it reveals *why* a particular candidate occupies its nominal position and, in the present screening, separates capacity-driven from SHE-driven candidates.

Table S10: Rank of each tested solvent when each of the four criteria is removed from the TOPSIS aggregation. Nominal rank in parentheses.

**Criterion removed**	**DMC (8)**	**CPME (27)**	**2-MeTHF (30)**	**2-BuOH (32)**
$c_{\log D_{\mathrm{G}}^{\mathrm{x},\infty}}$	8	31	30	25
$c_{\log D_{\mathrm{DMP}}^{\mathrm{x},\infty}}$	8	31	29	24
c_{CHEM21}	13	16	28	38
c_{BP}	11	30	33	29

Reading Table S10 candidate by candidate clarifies the role each criterion plays in supporting the four experimental selections. DMC moves between rank 8 and rank 13 across the four single-criterion drops, a window of five positions that, by the threshold introduced above, classifies it as *not load-bearing* on any individual criterion: its top-of-the-pool position is sustained by the joint contribution of capacity (both targets) and SHE/distillation considerations, not by one dominant axis. CPME shifts from rank 27 to rank 16 when CHEM21 is dropped (an 11-position gain), showing that the MCDA penalizes its position primarily through SHE (CHEM21 max = 7, driven by safety endpoints (low flash point)), not through capacity; without that penalty, CPME would already rank

in the upper third of the pool. The opposite signature is observed for 2-BuOH, which falls from rank 32 to rank 38 when CHEM21 is dropped, indicating that its inclusion in the four-solvent shortlist rests on its Recommended CHEM21 verdict rather than on thermodynamic capacity. This is the diagnostic signature of a thermodynamic negative reference and, as such, validates the role originally assigned to 2-BuOH in the experimental design: a Recommended candidate whose low predicted capacity should be *visible* in the laboratory measurements, providing a control against which the predictive power of the COSMO-RS-based screening can be gauged. 2-MeTHF is stable around rank 30 across the four drops; its inclusion in the experimental program is therefore supported neither by a peak TOPSIS score nor by a single distinctive criterion, but by an independent line of evidence: the documented record of 2-MeTHF as a bio-based extraction solvent for phenolic and oxygenated targets[14,15], which the present work tests rather than predicts.

Taken together, the dual Monte Carlo and the LOO analysis support three conclusions that frame the use of the TOPSIS ranking. First, the protocol reproducibly distinguishes a leading group (esters and DMC) from the bulk of the pool under both weight and log D uncertainty, so the identification of DMC as the highest-scoring carbonate-ester candidate is not an artifact of a particular weight choice. Second, no single criterion is solely responsible for that result: removing capacity (either target), CHEM21, or boiling point shifts ranks but does not collapse the leading cluster, so the aggregation is genuinely multi-criteria rather than a re-expression of one dominant axis. Third, the four solvents retained for experimental validation (DMC, CPME, 2-MeTHF, 2-BuOH) span complementary positions in this stability landscape: DMC as a robust top-of-the-pool candidate, CPME as a SHE-penalized but thermodynamically competitive ether, 2-BuOH as a Recommended low-capacity reference, and 2-MeTHF as a literature-supported bio-based ether. The diversity of these supporting profiles, made explicit by the LOO breakdown, is what makes the four-solvent experimental program informative as a test of the predictive value of the screening rather than as a confirmation of its highest-ranked candidate alone.

Ternary phase diagram

Table S11: Ternary phase diagram compositions for DMC + {SynBO or Bio-oil} + Water systems at 313.15 K

SynBO system				Bio-oil system			
**DMC / wt%**	**SynBO / wt%**	**Water / wt%**	**2 Phases?**	**DMC / wt%**	**Bio-oil / wt%**	**Water / wt%**	**2 Phases?**
91.81	5.43	2.76	Yes	94.26	0.00	5.74	No
80.43	9.86	9.70	Yes	87.78	6.74	5.49	No
77.95	8.90	13.15	Yes	79.99	5.04	14.96	Yes
69.85	20.11	10.03	Yes	79.72	9.91	10.37	No
68.84	6.59	24.57	Yes	74.91	5.07	20.02	Yes
68.12	10.83	21.05	Yes	74.13	16.74	9.13	No
62.14	8.79	29.06	Yes	71.33	21.51	7.16	Yes
61.80	19.67	18.53	Yes	69.50	20.43	10.07	Yes
58.52	8.80	32.68	Yes	69.15	10.59	20.26	Yes
50.69	8.44	40.87	Yes	59.15	10.99	29.86	Yes
50.34	30.29	19.36	Yes	58.88	20.71	20.42	Yes
50.30	10.72	38.98	Yes	56.37	32.73	10.89	No
49.72	19.63	30.65	Yes	51.00	19.70	29.30	Yes
41.30	19.21	39.49	Yes	50.17	0.00	49.83	Yes
40.90	9.74	49.35	Yes	50.06	5.10	44.84	Yes
40.76	29.16	30.08	Yes	49.24	29.62	21.13	Yes
40.26	39.89	19.86	Yes	44.97	34.82	20.22	No
33.31	7.69	59.00	Yes	44.79	5.05	50.16	Yes
31.42	38.90	29.68	Yes	43.54	14.04	42.43	Yes
30.38	9.99	59.63	Yes	40.25	29.91	29.84	Yes
29.87	20.07	50.06	Yes	39.94	19.90	40.17	Yes
29.85	30.07	40.08	Yes	39.94	39.90	20.16	No
27.29	7.31	65.40	Yes	39.70	9.84	50.46	Yes (PL)
21.42	18.66	59.92	Yes	35.57	34.69	29.74	Yes
20.91	9.84	69.24	Yes	34.07	49.46	16.46	No
19.97	49.81	30.21	Yes	30.10	49.82	20.09	No
19.96	39.73	40.31	Yes	30.04	9.85	60.11	Yes (PL)
19.77	7.72	72.51	Yes	29.96	20.01	50.04	Yes (PL)
19.71	30.36	49.93	Yes	29.83	20.13	40.21	Yes
10.69	5.62	83.69	Yes	29.74	29.97	40.30	Yes
10.18	59.95	29.86	Yes	29.70	39.74	30.56	No
10.17	10.22	79.61	Yes	25.24	29.76	45.01	Yes
9.99	49.63	40.37	Yes	20.39	54.08	25.53	No
9.91	39.83	50.26	Yes	20.32	44.59	35.09	Yes
9.84	20.52	69.64	Yes	20.15	39.84	40.01	Yes
9.74	30.17	60.10	Yes	20.08	10.13	69.79	Yes (PL)
5.19	34.75	60.06	Yes	20.01	50.01	29.98	No
4.81	14.73	80.46	Yes	19.99	30.08	49.93	Yes (PL)
4.76	44.92	50.32	Yes	19.92	40.08	40.01	Yes
4.73	25.64	69.63	Yes	19.00	19.27	61.73	Yes (PL)
4.60	55.78	39.62	Yes	15.14	49.89	34.97	Yes
4.60	5.01	90.38	Yes	14.77	39.36	45.87	Yes (PL)
–	–	–	–	10.46	39.70	49.84	Yes (PL)
–	–	–	–	10.10	59.83	30.06	No
–	–	–	–	10.00	49.98	40.02	Yes
–	–	–	–	9.94	10.22	79.84	Yes (PL)
–	–	–	–	9.93	20.41	69.66	Yes (PL)
–	–	–	–	9.90	49.45	40.64	Yes
–	–	–	–	9.90	31.55	58.55	Yes (PL)
–	–	–	–	5.19	50.12	44.69	Yes (PL)
–	–	–	–	5.19	59.78	35.03	Yes
–	–	–	–	0.00	69.76	30.24	Yes
–	–	–	–	0.00	65.07	34.93	Yes (PL)

Table S12: Ternary phase diagram compositions for CPME + {SynBO or Bio-oil} + Water systems at 313.15 K

SynBO system				Bio-oil system			
**CPME / wt%**	**SynBO / wt%**	**Water / wt%**	**2 Phases?**	**CPME / wt%**	**Bio-oil / wt%**	**Water / wt%**	**2 Phases?**
80.46	5.38	14.16	Yes	64.85	30.09	5.06	No
77.57	11.04	11.40	Yes	61.00	27.52	11.49	No
70.56	19.94	9.50	Yes	60.26	29.90	9.84	No
69.99	10.04	19.97	Yes	59.90	35.03	5.07	Yes
69.99	5.40	24.61	Yes	55.11	25.02	19.87	No
60.42	9.92	29.66	Yes	53.50	35.78	10.72	Yes
59.74	20.12	20.14	Yes	53.10	25.23	21.67	No
50.11	30.00	19.89	Yes	50.46	44.66	4.88	Yes (PL)
49.96	5.76	44.29	Yes	50.18	30.03	19.79	Yes
48.54	20.49	30.97	Yes	49.46	40.02	10.52	Yes
47.81	10.42	41.78	Yes	46.83	20.28	32.89	No
40.91	9.59	49.50	Yes	44.82	24.96	30.22	No
40.52	19.79	39.68	Yes	43.48	35.61	20.90	Yes
40.28	30.21	29.51	Yes	39.91	40.38	19.72	Yes
39.87	40.13	20.00	Yes	39.88	29.85	30.27	Yes
30.95	5.21	63.84	Yes	39.87	13.27	46.86	Yes
30.73	39.64	29.64	Yes	39.70	50.10	10.21	Yes (PL)
30.60	19.94	49.46	Yes	39.59	20.34	40.07	No
30.51	9.73	59.77	Yes	38.62	34.24	27.14	Yes
30.32	29.86	39.82	Yes	38.46	51.22	10.32	Yes (PL)
20.62	5.61	73.77	Yes	38.28	51.46	10.25	Yes (PL)
20.40	39.53	40.07	Yes	38.25	30.07	31.68	Yes
20.34	49.91	29.76	Yes	37.80	19.54	42.66	Yes
20.27	29.78	49.95	Yes	37.70	40.95	21.36	Yes
19.99	20.12	59.89	Yes	37.38	12.44	50.18	Yes
19.57	10.11	70.32	Yes	34.92	19.91	45.17	Yes
12.70	11.73	75.57	Yes	34.57	12.65	52.78	Yes
12.23	21.13	66.64	Yes	30.10	39.78	30.12	Yes
11.49	31.04	57.48	Yes	29.89	50.06	20.05	Yes (PL)
11.01	43.15	45.85	Yes	29.88	60.16	9.96	Yes (PL)
9.96	60.01	30.02	Yes	29.81	44.73	25.45	Yes (PL)
9.92	50.20	39.89	Yes	29.71	20.26	50.02	Yes
5.27	15.34	79.38	Yes	29.66	30.29	40.05	Yes
5.22	35.14	59.64	Yes	29.25	50.54	20.22	Yes (PL)
5.19	5.85	88.95	Yes	25.38	34.85	39.78	Yes
5.16	45.01	49.83	Yes	21.83	49.01	29.16	Yes (PL)
5.09	25.61	69.30	Yes	21.44	59.24	19.32	Yes (PL)
5.07	54.78	40.15	Yes	20.69	39.61	39.70	Yes (PL)
–	–	–	–	20.43	19.89	59.68	Yes
–	–	–	–	20.15	49.78	30.07	Yes (PL)
–	–	–	–	20.15	10.07	69.78	Yes
–	–	–	–	19.94	29.03	51.03	Yes
–	–	–	–	19.93	70.14	9.93	Yes (PL)
–	–	–	–	19.37	40.46	40.17	Yes (PL)
–	–	–	–	18.19	35.86	45.95	Yes (PL)
–	–	–	–	14.68	34.70	50.62	Yes (PL)
–	–	–	–	14.66	19.55	65.79	Yes
–	–	–	–	14.34	12.80	72.87	Yes
–	–	–	–	10.80	10.13	79.07	Yes
–	–	–	–	10.33	59.74	29.92	Yes (PL)
–	–	–	–	10.22	69.96	19.82	Yes (PL)
–	–	–	–	10.11	39.90	49.99	Yes (PL)
–	–	–	–	9.95	79.94	10.11	Yes (PL)
–	–	–	–	9.91	29.80	60.29	Yes (PL)
–	–	–	–	9.85	50.34	39.81	Yes (PL)
–	–	–	–	8.29	19.56	72.16	Yes (PL)
–	–	–	–	6.31	10.57	83.13	Yes (PL)
–	–	–	–	6.23	5.34	88.43	Yes
–	–	–	–	5.36	6.69	87.95	Yes (PL)
–	–	–	–	5.26	44.53	50.21	Yes (PL)
–	–	–	–	5.24	10.06	84.70	Yes (PL)
–	–	–	–	5.23	29.65	65.12	Yes (PL)
–	–	–	–	3.08	2.02	94.90	Yes (PL)

Table S13: Ternary phase diagram compositions for 2-MeTHF + {SynBO or Bio-oil} + Water systems at 313.15 K

SynBO system				Bio-oil system			
**2-MeTHF / wt%**	**SynBO / wt%**	**Water / wt%**	**2 Phases?**	**2-MeTHF / wt%**	**Bio-oil / wt%**	**Water / wt%**	**2 Phases?**
83.90	9.58	6.52	Yes	96.42	0.00	3.58	Yes
70.99	10.06	18.95	Yes	89.88	5.11	5.01	No
61.89	19.35	18.76	Yes	84.86	10.14	5.00	No
61.82	10.36	27.82	Yes	80.15	9.91	9.94	Yes
54.81	6.87	38.32	Yes	79.97	5.10	14.93	Yes
51.16	29.70	19.14	Yes	75.35	14.86	9.79	No
50.98	10.00	39.02	Yes	69.86	10.14	19.99	Yes
50.11	20.60	29.29	Yes	69.33	20.51	10.16	No
41.35	9.87	48.78	Yes	69.30	15.05	15.65	Yes
40.88	29.52	29.60	Yes	64.60	20.29	15.11	Yes
40.67	19.93	39.40	Yes	60.16	24.98	14.86	Yes
39.60	4.96	55.44	Yes	60.14	10.02	29.84	Yes
31.40	39.18	29.41	Yes	60.07	20.14	19.79	Yes
31.27	9.98	58.75	Yes	55.27	29.91	14.83	Yes
30.67	29.41	39.92	Yes	50.08	9.94	39.97	Yes
30.58	19.98	49.44	Yes	50.04	20.11	29.85	Yes
21.43	39.03	39.54	Yes	49.61	30.01	20.39	Yes
21.36	9.86	68.78	Yes	45.14	35.01	19.85	Yes
20.74	19.74	59.52	Yes	45.00	40.03	14.97	No
20.74	49.55	29.71	Yes	40.22	30.06	29.72	Yes
20.06	29.82	50.12	Yes	40.14	9.96	49.90	Yes
19.98	48.63	31.39	Yes	40.04	20.52	39.44	Yes
16.86	5.26	77.89	Yes	39.89	40.19	19.92	Yes
15.81	58.53	25.66	Yes	35.10	44.22	20.67	Yes
12.66	46.27	41.07	Yes	30.61	44.27	25.12	Yes
10.32	39.72	49.97	Yes	30.50	20.04	49.46	Yes
10.28	29.80	59.92	Yes	30.24	29.82	39.94	Yes
10.19	19.59	70.23	Yes	30.21	10.18	59.60	Yes
10.14	10.08	79.77	Yes	29.97	40.15	29.88	Yes
10.11	50.41	39.48	Yes	29.58	50.30	20.12	No
5.10	4.10	90.80	Yes	29.06	45.93	25.01	Yes
4.90	14.09	81.01	Yes	20.41	54.69	24.90	No
4.81	24.75	70.43	Yes	20.23	49.83	29.94	Yes
4.80	34.73	60.47	Yes	20.13	39.93	39.94	Yes
4.39	57.35	38.26	Yes	19.93	10.10	69.97	Yes
–	–	–	–	19.89	20.29	59.81	Yes
–	–	–	–	19.21	31.29	49.50	Yes
–	–	–	–	15.34	54.28	30.37	No
–	–	–	–	15.18	59.67	25.14	No
–	–	–	–	14.99	29.90	55.11	Yes (PL)
–	–	–	–	14.89	19.89	65.22	Yes (PL)
–	–	–	–	14.83	40.00	45.17	Yes (PL)
–	–	–	–	14.70	6.11	79.19	Yes
–	–	–	–	10.83	29.65	59.52	Yes (PL)
–	–	–	–	10.29	10.06	79.65	Yes (PL)
–	–	–	–	10.29	49.72	39.99	Yes (PL)
–	–	–	–	10.28	54.71	35.01	No
–	–	–	–	10.27	19.97	69.75	Yes (PL)
–	–	–	–	10.06	40.21	49.73	Yes (PL)
–	–	–	–	9.97	59.92	30.10	No
–	–	–	–	9.56	0.00	90.44	Yes
–	–	–	–	5.55	17.00	77.44	Yes (PL)
–	–	–	–	5.24	28.12	66.64	Yes (PL)
–	–	–	–	5.18	64.59	30.24	No
–	–	–	–	5.03	53.09	41.88	Yes (PL)
–	–	–	–	4.89	4.99	90.12	Yes (PL)
–	–	–	–	4.80	39.66	55.54	Yes (PL)

Table S14: Ternary phase diagram compositions for 2-Butanol + {SynBO or Bio-oil} + Water systems at 313.15 K

SynBO system				Bio-oil system			
2-Butanol / wt%	SynBO / wt%	Water / wt%	2 Phases?	2-Butanol / wt%	Bio-oil / wt%	Water / wt%	2 Phases?
79.99	10.34	9.66	No	80.15	9.84	10.02	No
75.49	3.56	20.95	No	70.90	9.76	19.33	No
73.80	17.13	9.06	No	70.20	19.78	10.02	No
70.72	8.29	21.00	No	66.75	0.00	33.25	Yes
70.18	9.95	19.86	No	60.40	19.83	19.76	No
69.26	4.04	26.70	No	60.22	10.04	29.75	No
68.76	0.00	31.24	Yes	53.97	5.58	40.44	Yes
66.96	0.00	33.04	Yes	50.24	29.73	20.03	No
66.36	0.00	33.64	Yes	50.04	10.20	39.75	Yes
65.22	5.98	28.80	No	49.70	5.11	45.20	Yes
64.90	18.38	16.72	No	49.68	19.88	30.45	No
60.21	25.68	14.11	No	49.53	9.90	40.57	Yes
60.07	20.17	19.76	No	45.28	9.96	44.76	Yes
59.96	15.18	24.87	No	45.13	10.04	44.83	Yes
58.89	13.45	27.66	No	40.36	20.08	39.57	No
57.55	19.50	22.95	No	40.23	39.90	19.88	No
57.39	0.00	42.61	Yes	40.12	15.16	44.72	No
54.94	5.03	40.03	Yes	39.95	30.05	30.00	No
49.71	18.13	32.16	No	39.94	10.11	49.95	Yes
49.30	20.24	30.46	No	34.94	20.24	44.83	No
49.12	10.28	40.60	Yes	30.32	39.71	29.98	No
47.88	8.79	43.23	Yes	30.00	24.98	45.01	No
46.72	11.83	41.45	Yes	29.93	29.83	40.24	No
40.68	40.01	19.31	No	29.86	20.34	49.80	Yes
39.95	19.81	40.24	No	29.43	10.94	59.64	Yes
39.49	15.20	45.32	No	24.89	29.89	45.22	No
39.41	30.56	30.02	No	20.31	10.10	69.60	Yes
39.32	10.25	50.43	Yes	20.17	40.15	39.68	No
38.22	18.92	42.86	No	20.16	29.88	49.96	Yes
37.11	15.01	47.88	Yes	19.93	50.32	29.74	No
31.08	9.93	58.98	Yes	19.75	19.92	60.33	Yes
30.06	39.55	30.39	No	17.64	34.38	47.98	No
29.98	17.97	52.05	No	15.60	0.00	84.40	Yes
29.83	20.23	49.94	No	14.98	10.25	74.77	Yes
29.58	9.39	61.03	Yes	14.91	29.94	55.16	Yes
29.54	15.18	55.27	Yes	14.83	20.25	64.92	Yes
29.07	5.75	65.18	Yes	14.69	4.98	80.33	Yes
28.80	30.76	40.44	No	12.74	10.11	77.16	Yes (PL)
26.84	4.57	68.59	Yes	12.07	40.07	47.86	Yes (PL)
25.84	14.92	59.24	No	12.04	30.13	57.83	Yes (PL)
21.69	49.01	29.30	No	12.01	5.06	82.94	Yes (PL)
21.04	29.80	49.16	No	11.92	20.08	68.00	Yes (PL)
20.96	8.16	70.88	Yes	10.32	59.72	29.95	No
20.02	39.33	40.65	No	10.23	9.96	79.81	Yes (PL)
19.68	20.30	60.02	No	10.15	49.96	39.88	No
15.46	0.00	84.54	Yes	10.11	40.50	49.39	Yes (PL)
10.65	39.55	49.80	No	10.03	29.60	60.37	Yes (PL)
10.63	29.62	59.75	No	9.93	19.79	70.28	Yes (PL)
10.46	59.75	29.78	No	5.10	28.17	66.73	Yes (PL)
10.40	10.12	79.49	No	5.06	54.69	40.25	No
10.01	20.41	69.58	No	4.86	5.00	90.13	Yes (PL)
9.10	50.66	40.24	No	4.73	50.36	44.91	Yes (PL)
–	–	–	–	4.68	16.48	78.84	Yes (PL)
–	–	–	–	4.53	42.26	53.21	Yes (PL)

Ternary systems

Table S15: Experimental LLE data for the ternary system water + acetol + guaiacol at 313.15 K

Tie-line	Phase I			Phase II		
	w_{water}	w_{acetol}	$w_{guaiacol}$	w_{water}	w_{acetol}	$w_{guaiacol}$
1	0.8932	0.0591	0.0478	0.0637	0.0344	0.9019
2	0.8273	0.1258	0.0469	0.0817	0.0765	0.8418
3	0.7134	0.1961	0.0906	0.1082	0.1211	0.7707

Table S16: Experimental LLE data for the ternary system water + acetic acid + furfural at 313.15 K

Tie-line	Phase I			Phase II		
	w_{water}	$w_{acetic\ acid}$	$w_{furfural}$	w_{water}	$w_{acetic\ acid}$	$w_{furfural}$
1	0.8149	0.0564	0.1287	0.0894	0.0414	0.8692
2	0.8025	0.0683	0.1291	0.1317	0.0663	0.8020
3	0.6686	0.1418	0.1897	0.2950	0.1926	0.5124

Table S17: Experimental LLE data for the ternary system water + 2,6-dimethoxyphenol + guaiacol at 313.15 K

Tie-line	Phase I			Phase II		
	w_{water}	$w_{2,6\text{-}DMP}$	$w_{guaiacol}$	w_{water}	$w_{2,6\text{-}DMP}$	$w_{guaiacol}$
1	0.9734	0.0015	0.0250	0.0525	0.0774	0.8702
2	0.9633	0.0053	0.0314	0.0576	0.1569	0.7855
3	0.9698	0.0037	0.0265	0.0537	0.1445	0.8018

Table S18: Experimental LLE data for the ternary system water + D-fructose + cyclopentyl methyl ether at 313.15 K

Tie-line	Phase I			Phase II		
	w_{water}	$w_{D\text{-}fructose}$	w_{CPME}	w_{water}	$w_{D\text{-}fructose}$	w_{CPME}
1	0.8261	0.1701	0.0039	0.0052	0.0007	0.9941
2	0.7618	0.2344	0.0038	0.0066	0.0002	0.9932
3	0.6856	0.3117	0.0026	0.0059	0.0001	0.9940

Table S19: Experimental LLE data for the ternary system water + acetol + furfural at 313.15 K

Tie-line	Phase I			Phase II		
	w_{water}	w_{acetol}	$w_{furfural}$	w_{water}	w_{acetol}	$w_{furfural}$
1	0.8201	0.0702	0.1097	0.0661	0.0253	0.9086
2	0.7412	0.1300	0.1287	0.0897	0.0604	0.8499
3	0.6516	0.1766	0.1718	0.1114	0.0962	0.7925

Table S20: Experimental LLE data for the ternary system water + acetic acid + cyclopentyl methyl ether at 313.15 K

Tie-line	Phase I			Phase II		
	w_{water}	$w_{acetic\ acid}$	w_{CPME}	w_{water}	$w_{acetic\ acid}$	w_{CPME}
1	0.0115	0.0290	0.9595	0.9233	0.0690	0.0076
2	0.0137	0.0686	0.9177	0.8413	0.1464	0.0123
3	0.0308	0.1172	0.8519	0.7643	0.2164	0.0193

Table S21: Experimental LLE data for the ternary system water + 2,6-dimethoxyphenol + furfural at 313.15 K

Tie-line	Phase I			Phase II		
	w_{water}	$w_{2,6\text{-}DMP}$	$w_{furfural}$	w_{water}	$w_{2,6\text{-}DMP}$	$w_{furfural}$
1	0.9198	0.0025	0.0777	0.0572	0.0885	0.8543
2	0.9251	0.0050	0.0699	0.0743	0.1938	0.7319
3	0.9278	0.0073	0.0649	0.0632	0.2355	0.7013

Table S22: Experimental LLE data for the ternary system water + D-fructose + dimethyl carbonate at 313.15 K

Tie-line	Phase I			Phase II		
	w_{water}	$w_{D\text{-}fructose}$	w_{DMC}	w_{water}	$w_{D\text{-}fructose}$	w_{DMC}
1	0.7496	0.1674	0.0830	0.0404	0.0000	0.9596
2	0.6515	0.2914	0.0571	0.0322	0.0005	0.9673

Table S23: Experimental LLE data for the ternary system water + acetol + cyclopentyl methyl ether at 313.15 K

Tie-line	Phase I			Phase II		
	w_{water}	w_{acetol}	w_{CPME}	w_{water}	w_{acetol}	w_{CPME}
1	0.0056	0.0039	0.9905	0.9165	0.0777	0.0059
2	0.0075	0.0092	0.9833	0.8343	0.1595	0.0063
3	0.0071	0.0160	0.9769	0.7395	0.2518	0.0087

Table S24: Experimental LLE data for the ternary system water + acetic acid + dimethyl carbonate at 313.15 K

**Tie-line**	**Phase I**			**Phase II**		
	w_{water}	$w_{\text{acetic acid}}$	w_{DMC}	w_{water}	$w_{\text{acetic acid}}$	w_{DMC}
1	0.7965	0.0527	0.1509	0.0838	0.0343	0.8818
2	0.6731	0.1142	0.2127	0.1341	0.0951	0.7708
3	0.6098	0.1321	0.2581	0.2237	0.1181	0.6582

Table S25: Experimental LLE data for the ternary system water + 2,6-dimethoxyphenol + cyclopentyl methyl ether at 313.15 K

**Tie-line**	**Phase I**			**Phase II**		
	w_{water}	$w_{\text{2,6-DMP}}$	w_{CPME}	w_{water}	$w_{\text{2,6-DMP}}$	w_{CPME}
1	0.0085	0.1718	0.8198	0.9656	0.0070	0.0064
2	0.0094	0.2398	0.7508	0.9749	0.0095	0.0055
3	0.0143	0.3146	0.6711	0.9641	0.0124	0.0064

Table S26: Experimental LLE data for the ternary system water + D-fructose + 2-methyltetrahydrofuran at 313.15 K

**Tie-line**	**Phase I**			**Phase II**		
	w_{water}	$w_{\text{D-fructose}}$	$w_{\text{2-MeTHF}}$	w_{water}	$w_{\text{D-fructose}}$	$w_{\text{2-MeTHF}}$
1	0.7917	0.1443	0.0640	0.0443	0.0004	0.9553
2	0.7303	0.2332	0.0366	0.0445	0.0000	0.9555
3	0.6509	0.3082	0.0409	0.0350	0.0000	0.9650

Table S27: Experimental LLE data for the ternary system water + acetol + dimethyl carbonate at 313.15 K

**Tie-line**	**Phase I**			**Phase II**		
	w_{water}	w_{acetol}	w_{DMC}	w_{water}	w_{acetol}	w_{DMC}
1	0.8063	0.0721	0.1216	0.0355	0.0176	0.9469
2	0.6970	0.1551	0.1480	0.0510	0.0440	0.9050
3	0.5970	0.2120	0.1911	0.0552	0.0819	0.8629

Table S28: Experimental LLE data for the ternary system water + acetic acid + 2-methyltetrahydrofuran at 313.15 K

**Tie-line**	**Phase I**			**Phase II**		
	w_{water}	$w_{\mathrm{acetic\ acid}}$	$w_{\mathrm{2\text{-}MeTHF}}$	w_{water}	$w_{\mathrm{acetic\ acid}}$	$w_{\mathrm{2\text{-}MeTHF}}$
1	0.0941	0.0754	0.8305	0.8640	0.0426	0.0934
2	0.1485	0.1501	0.7014	0.7852	0.0980	0.1168
3	0.2788	0.2072	0.5140	0.6656	0.1583	0.1762

Table S29: Experimental LLE data for the ternary system water + 2,6-dimethoxyphenol + dimethyl carbonate at 313.15 K

**Tie-line**	**Phase I**			**Phase II**		
	w_{water}	$w_{\mathrm{2,6\text{-}DMP}}$	w_{DMC}	w_{water}	$w_{\mathrm{2,6\text{-}DMP}}$	w_{DMC}
1	0.9005	0.0053	0.0942	0.0536	0.1207	0.8257
2	0.8999	0.0093	0.0908	0.0664	0.2116	0.7220
3	0.9025	0.0130	0.0845	0.0686	0.2920	0.6393

Table S30: Experimental LLE data for the ternary system water + D-fructose + guaiacol at 313.15 K

**Tie-line**	**Phase I**			**Phase II**		
	w_{water}	$w_{\mathrm{D\text{-}fructose}}$	w_{guaiacol}	w_{water}	$w_{\mathrm{D\text{-}fructose}}$	w_{guaiacol}
1	0.7975	0.1794	0.0230	0.0483	0.0004	0.9513
2	0.7679	0.2072	0.0249	0.0462	0.0000	0.9538
3	0.6856	0.2803	0.0340	0.0661	0.0000	0.9339

Table S31: Experimental LLE data for the ternary system water + acetol + 2-methyltetrahydrofuran at 313.15 K

**Tie-line**	**Phase I**			**Phase II**		
	w_{water}	w_{acetol}	$w_{\mathrm{2\text{-}MeTHF}}$	w_{water}	w_{acetol}	$w_{\mathrm{2\text{-}MeTHF}}$
1	0.0351	0.0173	0.9476	0.8498	0.0748	0.0754
2	0.0476	0.0397	0.9127	0.7526	0.1523	0.0951
3	0.0632	0.0718	0.8650	0.6804	0.2174	0.1021

Table S32: Experimental LLE data for the ternary system water + acetic acid + guaiacol at 313.15 K

Tie-line	Phase I			Phase II		
	w_{water}	$w_{\text{acetic acid}}$	w_{guaiacol}	w_{water}	$w_{\text{acetic acid}}$	w_{guaiacol}
1	0.9065	0.0594	0.0341	0.0731	0.0253	0.9016
2	0.8762	0.0840	0.0399	0.0814	0.0383	0.8803
3	0.7734	0.1545	0.0721	0.1088	0.0831	0.8081

Table S33: Experimental LLE data for the ternary system water + 2,6-dimethoxyphenol + 2-methyltetrahydrofuran at 313.15 K

Tie-line	Phase I			Phase II		
	w_{water}	$w_{\text{2,6-DMP}}$	$w_{\text{2-MeTHF}}$	w_{water}	$w_{\text{2,6-DMP}}$	$w_{\text{2-MeTHF}}$
1	0.0548	0.1303	0.8150	0.9120	0.0044	0.0836
2	0.0622	0.2067	0.7311	0.8070	0.0403	0.1527
3	0.0582	0.2826	0.6592	0.8549	0.0328	0.1123

Table S34: Experimental LLE data for the ternary system water + D-fructose + furfural at 313.15 K

Tie-line	Phase I			Phase II		
	w_{water}	$w_{\text{D-fructose}}$	w_{furfural}	w_{water}	$w_{\text{D-fructose}}$	w_{furfural}
1	0.8525	0.0725	0.0750	0.0617	0.0000	0.9383
2	0.7416	0.1914	0.0670	0.0605	0.0000	0.9395
3	0.6492	0.2966	0.0542	0.0627	0.0000	0.9373

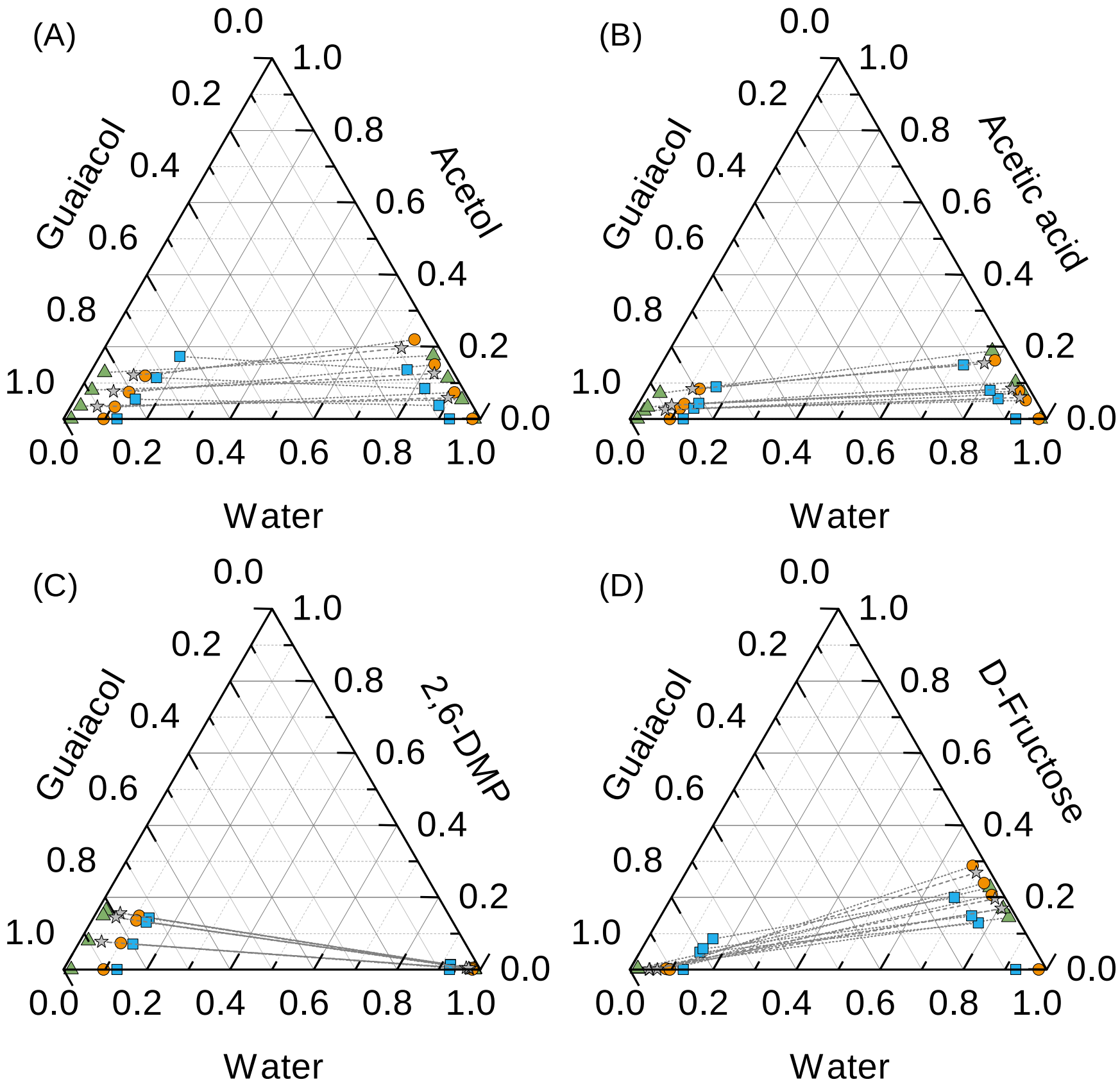

Figure S1: Ternary liquid–liquid equilibrium (LLE) phase diagrams for the systems Water + Molecule 2 + Guaiacol at 313.15 K, where Molecule 2 corresponds to (A) Acetol, (B) Acetic acid, (C) 2,6-Dimethoxyphenol (2,6-DMP), and (D) D-Fructose. ☆ denote experimental data, △ represent COSMO-RS predictions, ● correspond to PC-SAFT calculations, and ■ represent UNIFAC 2.0 predictions.

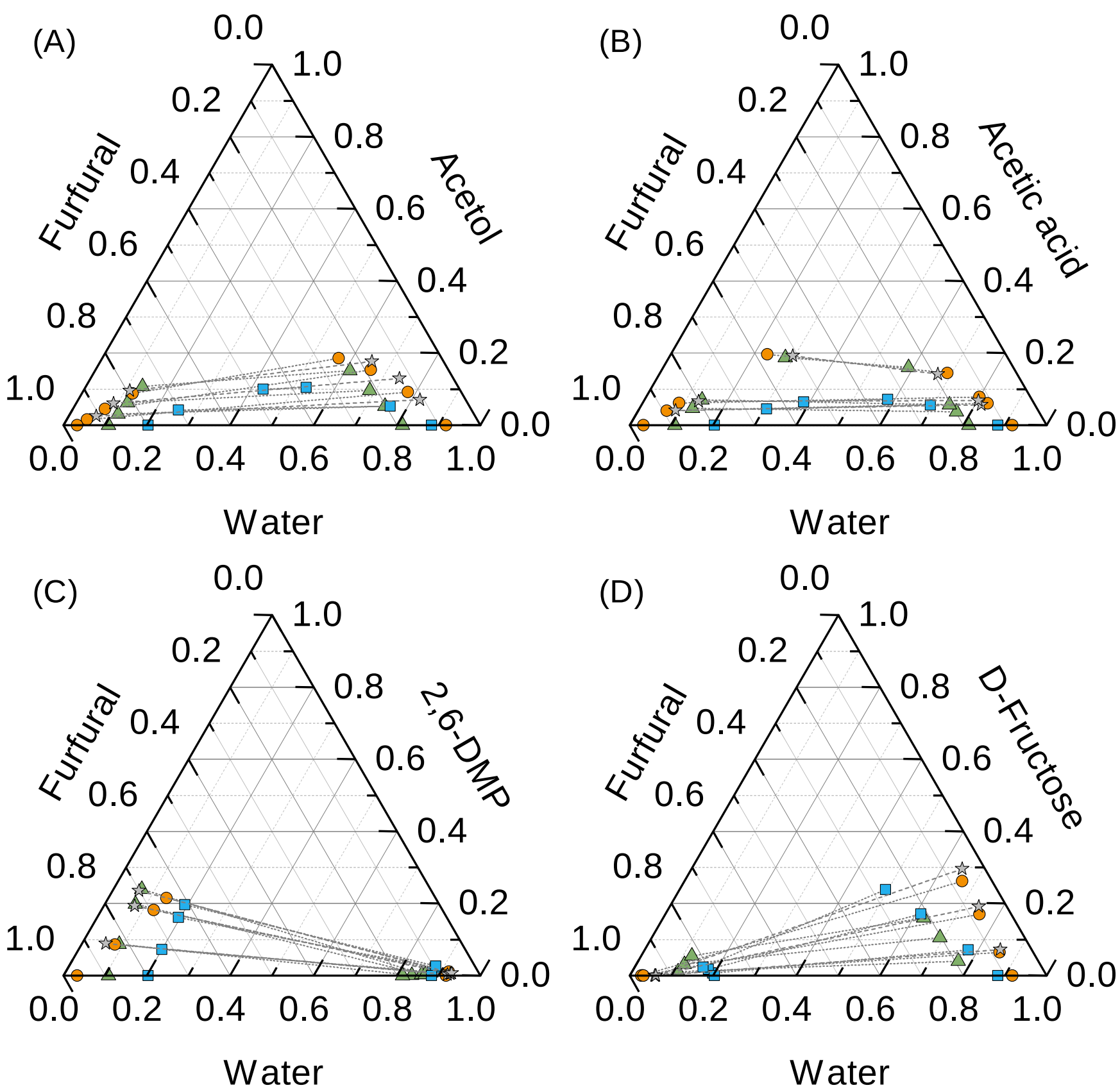

Figure S2: Ternary liquid–liquid equilibrium (LLE) phase diagrams for the systems Water + Molecule 2 + Furfural at 313.15 K, where molecule 2 corresponds to (A) Acetol, (B) Acetic acid, (C) 2,6-Dimethoxyphenol (2,6-DMP), and (D) D-Fructose. ☆ denote experimental data, ● correspond to PC-SAFT calculations, △ represent COSMO-RS predictions, and ■ represent UNIFAC 2.0 predictions.

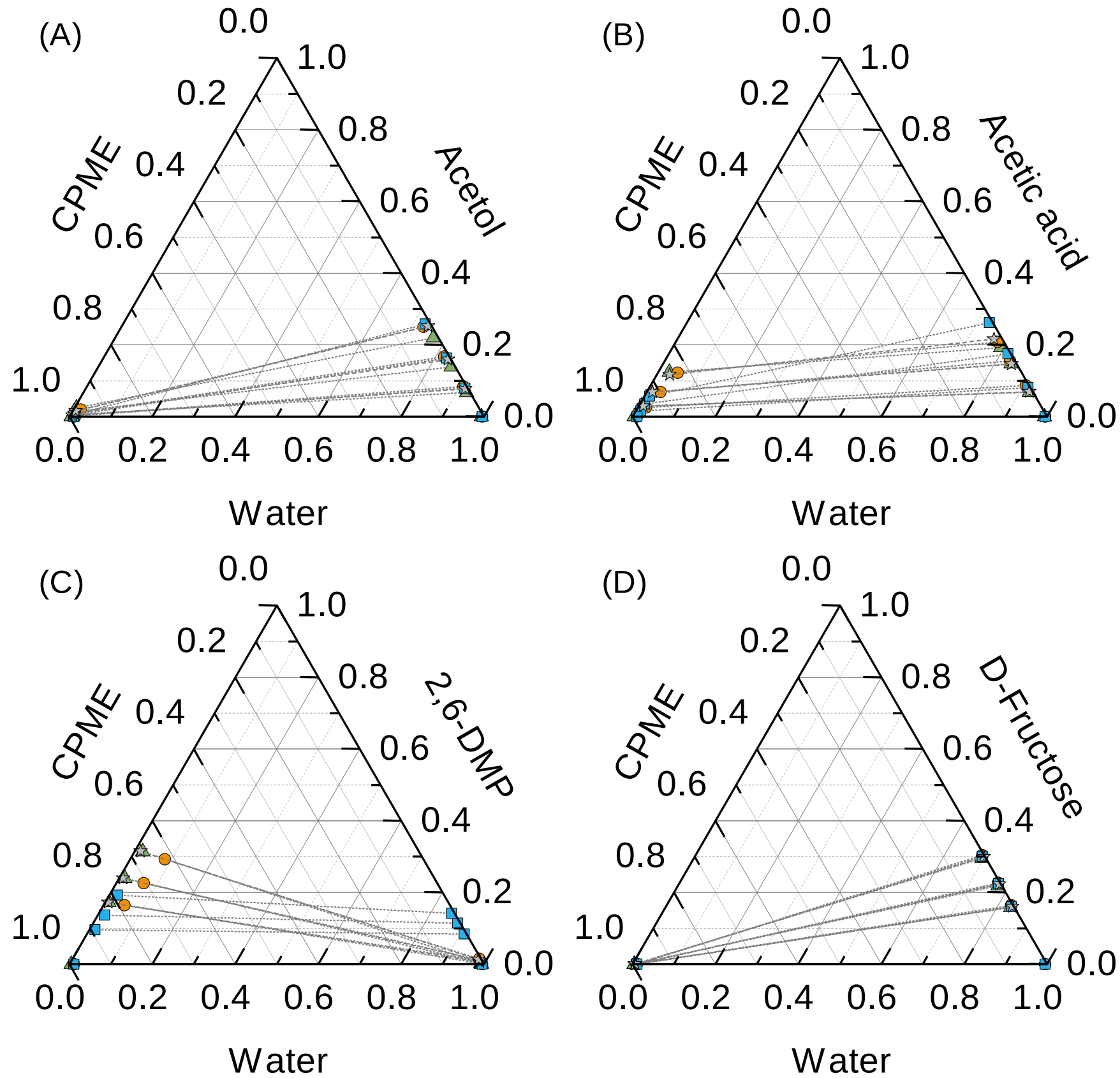

Figure S3: Ternary liquid–liquid equilibrium (LLE) phase diagrams for the systems Water + Molecule 2 + CPME at 313.15 K, where molecule 2 corresponds to (A) Acetol, (B) Acetic acid, (C) 2,6-Dimethoxyphenol (2,6-DMP), and (D) D-Fructose. ☆ denote experimental data, ● correspond to PC-SAFT calculations, △ represent COSMO-RS predictions, and ■ represent UNIFAC 2.0 predictions.

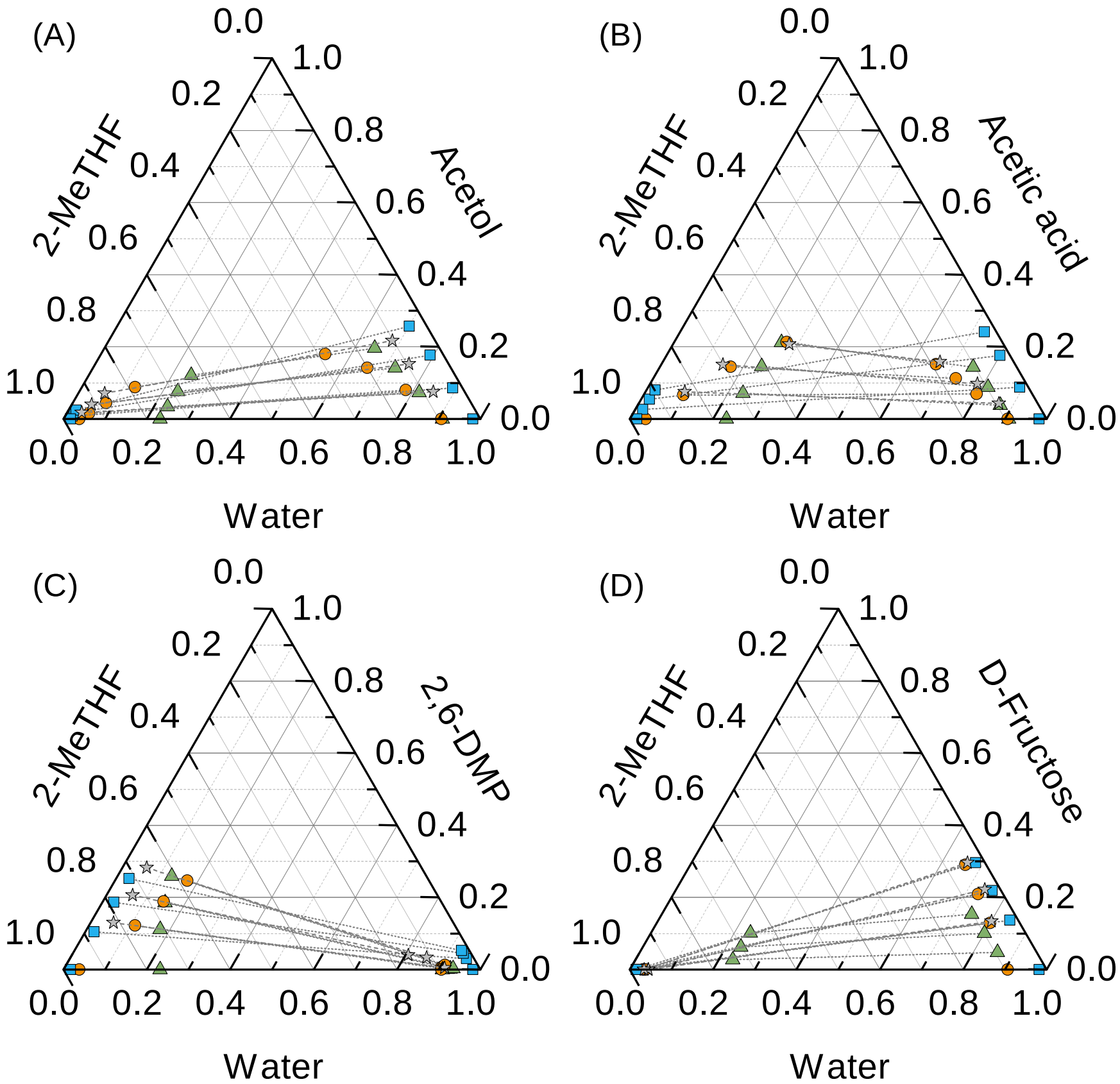

Figure S4: Ternary liquid–liquid equilibrium (LLE) phase diagrams for the systems Water + Molecule 2 + 2-MeTHF at 313.15 K, where molecule 2 corresponds to (A) Acetol, (B) Acetic acid, (C) 2,6-Dimethoxyphenol (2,6-DMP), and (D) D-Fructose. ☆ denote experimental data, ● correspond to PC-SAFT calculations, ▲ represent COSMO-RS predictions, and ■ represent UNIFAC 2.0 predictions.

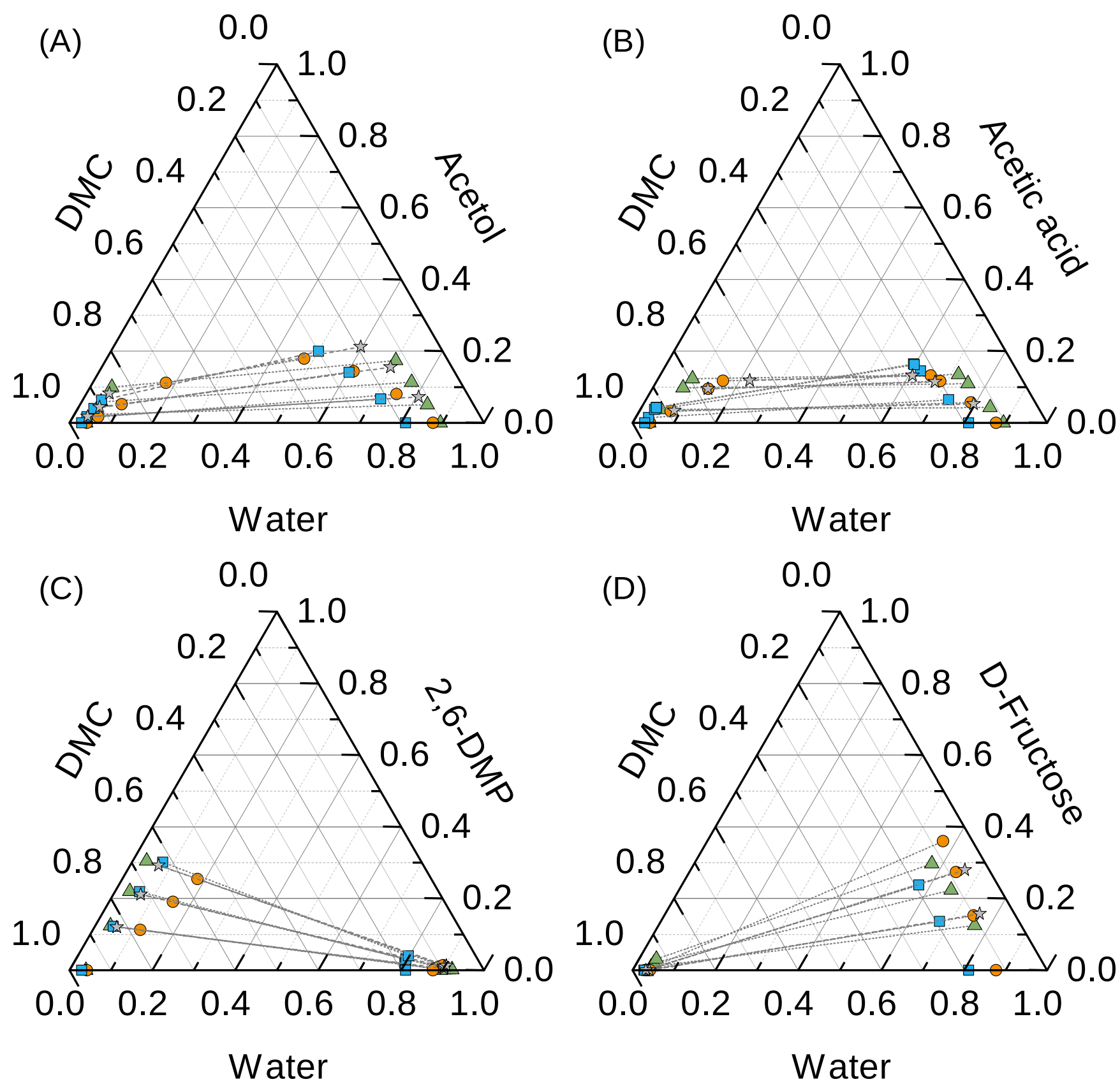

Figure S5: Ternary liquid–liquid equilibrium (LLE) phase diagrams for the systems Water + Molecule 2 + DMC at 313.15 K, where molecule 2 corresponds to (A) Acetol, (B) Acetic acid, (C) 2,6-Dimethoxyphenol (2,6-DMP), and (D) D-Fructose. ☆ denote experimental data, • correspond to PC-SAFT calculations, △ represent COSMO-RS predictions, and □ represent UNIFAC 2.0 predictions.

Multicomponent systems

Table S35: Experimental multicomponent liquid-liquid equilibrium (LLE) data for water + SynBO + organic solvent systems at 313.15 K. Organic solvents reported: dimethyl carbonate (DMC), cyclopentyl methyl ether (CPME), and 2-methyltetrahydrofuran (2-MeTHF).

	**water + SynBO + DMC**					
	25 wt% OS		50 wt% OS		75 wt% OS	
	w_{aq}	w_{os}	w_{aq}	w_{os}	w_{aq}	w_{os}
Water	0.5860	0.1105	0.6087	0.0754	0.6207	0.0458
2,6-DMP	0.0102	0.0943	0.0029	0.0393	0.0007	0.0148
Guaiacol	0.0126	0.1437	0.0031	0.0589	0.0007	0.0218
Furfural	0.0088	0.0631	0.0037	0.0325	0.0013	0.0124
Acetic acid	0.0424	0.0379	0.0276	0.0270	0.0138	0.0136
Acetol	0.0828	0.0479	0.0662	0.0352	0.0422	0.0201
d-Fructose	0.1639	0.0050	0.1925	0.0015	0.2372	0.0000
DMC	0.0932	0.4977	0.0953	0.7303	0.0832	0.8716
	**water + SynBO + CPME**					
	25 wt% OS		50 wt% OS		75 wt% OS	
	w_{aq}	w_{os}	w_{aq}	w_{os}	w_{aq}	w_{os}
Water	0.6423	0.0238	0.6439	0.0154	0.6676	0.0104
2,6-DMP	0.0063	0.0893	0.0035	0.0470	0.0016	0.0149
Guaiacol	0.0050	0.0974	0.0022	0.0729	0.0015	0.0222
Furfural	0.0100	0.0735	0.0060	0.0389	0.0025	0.0124
Acetic acid	0.0478	0.0314	0.0398	0.0225	0.0203	0.0110
Acetol	0.1094	0.0203	0.1027	0.0132	0.0792	0.0076
D-Fructose	0.1720	0.0015	0.1963	0.0007	0.1838	0.0005
CPME	0.0072	0.6629	0.0055	0.7892	0.0435	0.9211
	**water + SynBO + 2-MeTHF**					
	25 wt% OS		50 wt% OS		75 wt% OS	
	w_{aq}	w_{os}	w_{aq}	w_{os}	w_{aq}	w_{os}
Water	0.6357	0.1104	0.6695	0.0838	0.7337	0.0599
2,6-DMP	0.0058	0.0846	0.0016	0.0354	0.0006	0.0136
Guaiacol	0.0051	0.1315	0.0011	0.0529	0.0004	0.0202
Furfural	0.0078	0.0570	0.0030	0.0266	0.0014	0.0109
Acetic acid	0.0338	0.0504	0.0166	0.0307	0.0073	0.0139
Acetol	0.0848	0.0368	0.0714	0.0283	0.0511	0.0173
D-fructose	0.1696	0.0044	0.1783	0.0095	0.1503	0.0013
2-MeTHF	0.0573	0.5249	0.0584	0.7328	0.0553	0.8629

Table S36: COSMO-RS predicted multicomponent liquid-liquid equilibrium (LLE) data for water + SynBO + organic solvent systems at 313.15 K. Organic solvents: dimethyl carbonate (DMC), cyclopentyl methyl ether (CPME), and 2-methyltetrahydrofuran (2-MeTHF).

	**water + SynBO + DMC**					
	25 wt% OS		50 wt% OS		75 wt% OS	
	w_{aq}	w_{os}	w_{aq}	w_{os}	w_{aq}	w_{os}
Water	0.663	0.055	0.688	0.051	0.733	0.046
2,6-DMP	0.002	0.093	0.001	0.037	0.000	0.013
Guaiacol	0.005	0.137	0.001	0.055	0.000	0.020
Furfural	0.015	0.069	0.007	0.033	0.002	0.013
Acetic acid	0.048	0.038	0.031	0.027	0.016	0.014
Acetol	0.088	0.052	0.070	0.039	0.043	0.023
d-Fructose	0.090	0.008	0.089	0.008	0.090	0.008
DMC	0.089	0.547	0.112	0.748	0.115	0.863
	**water + SynBO + CPME**					
	25 wt% OS		50 wt% OS		75 wt% OS	
	w_{aq}	w_{os}	w_{aq}	w_{os}	w_{aq}	w_{os}
Water	0.712	0.031	0.734	0.012	0.764	0.007
2,6-DMP	0.002	0.088	0.001	0.037	0.000	0.014
Guaiacol	0.003	0.131	0.002	0.056	0.001	0.021
Furfural	0.017	0.063	0.012	0.030	0.006	0.013
Acetic acid	0.049	0.038	0.039	0.022	0.025	0.012
Acetol	0.107	0.032	0.102	0.018	0.088	0.011
d-Fructose	0.098	0.003	0.100	0.000	0.105	0.000
CPME	0.011	0.614	0.012	0.824	0.011	0.922
	**water + SynBO + 2-MeTHF**					
	25 wt% OS		50 wt% OS		75 wt% OS	
	w_{aq}	w_{os}	w_{aq}	w_{os}	w_{aq}	w_{os}
Water	0.677	0.134	0.715	0.185	NO LLE	
2,6-DMP	0.002	0.079	0.001	0.029		
Guaiacol	0.004	0.118	0.001	0.043		
Furfural	0.016	0.059	0.008	0.027		
Acetic acid	0.037	0.052	0.019	0.031		
Acetol	0.097	0.049	0.084	0.042		
d-Fructose	0.084	0.026	0.068	0.032		
2-MeTHF	0.082	0.484	0.104	0.610		

Table S37: Modified UNIFAC 2.0 predicted multicomponent liquid-liquid equilibrium (LLE) data for water + SynBO + organic solvent systems at 313.15 K. Organic solvents: dimethyl carbonate (DMC), cyclopentyl methyl ether (CPME), and 2-methyltetrahydrofuran (2-MeTHF).

	**water + SynBO + DMC**					
	25 wt% OS		50 wt% OS		75 wt% OS	
	w_{aq}	w_{os}	w_{aq}	w_{os}	w_{aq}	w_{os}
Water	0.521	0.102	0.552	0.054	0.585	0.039
2,6-DMP	0.023	0.087	0.010	0.036	0.004	0.013
Guaiacol	0.033	0.141	0.012	0.056	0.006	0.020
Furfural	0.027	0.070	0.011	0.034	0.005	0.013
Acetic acid	0.047	0.024	0.042	0.015	0.033	0.010
Acetol	0.080	0.063	0.070	0.033	0.054	0.018
d-Fructose	0.071	0.006	0.080	0.001	0.097	0.000
DMC	0.197	0.506	0.223	0.771	0.220	0.886
	**water + SynBO + CPME**					
	25 wt% OS		50 wt% OS		75 wt% OS	
	w_{aq}	w_{os}	w_{aq}	w_{os}	w_{aq}	w_{os}
Water	0.628	0.016	0.663	0.014	0.703	0.012
2,6-DMP	0.038	0.025	0.029	0.018	0.017	0.010
Guaiacol	0.043	0.072	0.025	0.042	0.011	0.019
Furfural	0.034	0.035	0.023	0.023	0.011	0.011
Acetic acid	0.055	0.013	0.050	0.011	0.039	0.008
Acetol	0.105	0.008	0.109	0.006	0.110	0.005
d-Fructose	0.085	0.000	0.091	0.000	0.105	0.000
CPME	0.011	0.831	0.010	0.887	0.009	0.935
	**water + SynBO + 2-MeTHF**					
	25 wt% OS		50 wt% OS		75 wt% OS	
	w_{aq}	w_{os}	w_{aq}	w_{os}	w_{aq}	w_{os}
Water	0.656	0.032	0.690	0.023	0.728	0.020
2,6-DMP	0.023	0.057	0.014	0.029	0.007	0.013
Guaiacol	0.023	0.108	0.012	0.050	0.005	0.020
Furfural	0.023	0.058	0.012	0.030	0.005	0.013
Acetic acid	0.054	0.021	0.046	0.016	0.032	0.010
Acetol	0.100	0.030	0.099	0.018	0.088	0.012
d-Fructose	0.089	0.000	0.096	0.000	0.098	0.000
2-MeTHF	0.032	0.694	0.030	0.833	0.028	0.913

Table S38: PC-SAFT predicted multicomponent liquid-liquid equilibrium (LLE) data for water + SynBO + organic solvent systems at 313.15 K. Organic solvents: dimethyl carbonate (DMC), cyclopentyl methyl ether (CPME), and 2-methyltetrahydrofuran (2-MeTHF).

	**water + SynBO + DMC**					
	25 wt% OS		50 wt% OS		75 wt% OS	
	w_{aq}	w_{os}	w_{aq}	w_{os}	w_{aq}	w_{os}
Water	0.629	0.166	0.637	0.123	0.639	0.079
2,6-DMP	0.008	0.076	0.003	0.033	0.001	0.013
Guaiacol	0.010	0.118	0.003	0.051	0.001	0.019
Furfural	0.015	0.066	0.006	0.032	0.002	0.013
Acetic acid	0.042	0.044	0.030	0.028	0.019	0.014
Acetol	0.089	0.058	0.073	0.040	0.056	0.021
d-Fructose	0.097	0.001	0.116	0.000	0.158	0.000
DMC	0.111	0.471	0.133	0.693	0.125	0.841
	**water + SynBO + CPME**					
	25 wt% OS		50 wt% OS		75 wt% OS	
	w_{aq}	w_{os}	w_{aq}	w_{os}	w_{aq}	w_{os}
Water	0.719	0.072	0.735	0.040	0.748	0.024
2,6-DMP	0.006	0.074	0.004	0.034	0.003	0.013
Guaiacol	0.006	0.115	0.003	0.053	0.001	0.020
Furfural	0.012	0.066	0.006	0.033	0.002	0.013
Acetic acid	0.044	0.041	0.038	0.023	0.030	0.011
Acetol	0.100	0.045	0.097	0.024	0.091	0.012
d-Fructose	0.103	0.000	0.107	0.000	0.115	0.000
CPME	0.010	0.587	0.010	0.793	0.009	0.906
	**water + SynBO + 2-MeTHF**					
	25 wt% OS		50 wt% OS		75 wt% OS	
	w_{aq}	w_{os}	w_{aq}	w_{os}	w_{aq}	w_{os}
Water	0.658	0.192	0.679	0.127	0.684	0.076
2,6-DMP	0.006	0.069	0.002	0.032	0.001	0.013
Guaiacol	0.010	0.103	0.003	0.048	0.001	0.019
Furfural	0.014	0.060	0.006	0.031	0.002	0.013
Acetic acid	0.035	0.053	0.023	0.031	0.014	0.014
Acetol	0.081	0.072	0.064	0.046	0.050	0.022
d-Fructose	0.108	0.001	0.128	0.000	0.164	0.000
2-MeTHF	0.089	0.450	0.094	0.685	0.083	0.842

References

(1) Prat, D.; Wells, A.; Hayler, J.; Sneddon, H.; McElroy, C. R.; Abou-Shehada, S.; Dunn, P. J. CHEM21 selection guide of classical- and less classical-solvents. *Green Chem.* **2016**, *18*, 288–296.

(2) van Krevelen, D. W.; te Nijenhuis, K. *Properties of Polymers: Their Correlation with Chemical Structure; Their Numerical Estimation and Prediction from Additive Group Contributions*, 4th ed.; Elsevier: Amsterdam, 2009.

(3) Hansen, C. M. *Hansen Solubility Parameters: A User's Handbook*, 2nd ed.; CRC Press: Boca Raton, 2007.

(4) Jiang, G.; Lv, D.; Zhang, F.; Ning, A.; Gao, G.; Ren, Y.; Wang, Z. Selective extraction of monophenols from pyrolysis bio-oil based on a novel three-dimensional visualization model. *Separation and Purification Technology* **2021**, *272*, 118902.

(5) Niederquell, A.; Wyttenbach, N.; Kuentz, M. New Prediction Methods for Solubility Parameters Based on Molecular Sigma Profiles Using Pharmaceutical Materials. *International Journal of Pharmaceutics* **2018**, *546*, 137–144.

(6) Mathieu, D. Pencil and Paper Estimation of Hansen Solubility Parameters. *ACS Omega* **2018**, *3*, 17049–17056.

(7) Klamt, A. The COSMO and COSMO-RS Solvation Models. *WIREs Computational Molecular Science* **2018**, *8*, e1338.

(8) Santaella, M. A.; Buitrago-Suescún, O. Y.; Orjuela, A. Green Solvent Selection for Terpene Extraction from Hemp. *Chemical Engineering and Processing – Process Intensification* **2026**, *220*, 110657.

(9) Soukup-Carne, D.; López-Porfiri, P.; Bragagnolo, F. S.; Funari, C. S.; Fan, X.; González-Miquel, M.; Esteban, J. Extraction of 5-Hydroxymethylfurfural and Furfural in Aqueous

Biphasic Systems: A COSMO-RS Guided Approach to Greener Solvent Selection. *ACS Sustainable Chemistry & Engineering* **2024**,

(10) Wu, Y.; Li, W.; Vovers, J.; Lu, H. T.; Stevens, G. W.; Mumford, K. A. Investigation of Green Solvents for the Extraction of Phenol and Natural Alkaloids: Solvent and Extractant Selection. *Chemical Engineering Journal* **2022**, *443*, 136054.

(11) Tobiszewski, M.; Namieśnik, J.; Pena-Pereira, F. Environmental risk-based ranking of solvents using the combination of a multimedia model and multi-criteria decision analysis. *Green Chemistry* **2017**, *19*, 1034–1042.

(12) Zavadskas, E. K.; Turskis, Z.; Kildienė, S. State of art surveys of overviews on MCDM/MADM methods. *Technological and Economic Development of Economy* **2014**, *20*, 165–179.

(13) Arroyo-Avirama, A. F.; Carreño-Guzmán, S.; Lorenzo-Llanes, J.; Gajardo-Parra, N. F.; Santiago, R.; Held, C.; Palomar, J.; Canales, R. I. In Situ Product Recovery of -Ionone from a Fermentation Broth: Computational Solvent Selection and Process Design of Its Extraction and Purification. *ACS Sustainable Chemistry & Engineering* **2023**, *11*, 9065–9076.

(14) González-Miquel, M.; Díaz, I. Green solvent screening using modeling and simulation. *Current Opinion in Green and Sustainable Chemistry* **2021**, *29*, 100469.

(15) Männistö, M.; Pokki, J.-P.; Alopaeus, V. Quaternary and Ternary LLE Measurements for Solvent (2-Methyltetrahydrofuran and Cyclopentyl Methyl Ether) + Furfural + Acetic Acid + Water between 298 and 343 K. *The Journal of Chemical Thermodynamics* **2018**, *119*, 61–75.
